# Expanding Poly(lactic acid) (PLA) and Polyhydroxyalkanoates (PHAs) Applications: A Review on Modifications and Effects

**DOI:** 10.3390/polym13234271

**Published:** 2021-12-06

**Authors:** Ahmed Z. Naser, Ibrahim Deiab, Fantahun Defersha, Sheng Yang

**Affiliations:** School of Engineering, University of Guelph, Guelph, ON N1G 2W1, Canada; anaser@uoguelph.ca (A.Z.N.); ideiab@uoguelph.ca (I.D.); fdefersh@uoguelph.ca (F.D.)

**Keywords:** poly(lactic acid) (PLA), polyhydroxyalkanoates (PHAs), review, properties, plasticizers, polymer blends, fillers, polymer nanocomposites, degradation, sustainability, 3D printing

## Abstract

The high price of petroleum, overconsumption of plastic products, recent climate change regulations, the lack of landfill spaces in addition to the ever-growing population are considered the driving forces for introducing sustainable biodegradable solutions for greener environment. Due to the harmful impact of petroleum waste plastics on human health, environment and ecosystems, societies have been moving towards the adoption of biodegradable natural based polymers whose conversion and consumption are environmentally friendly. Therefore, biodegradable biobased polymers such as poly(lactic acid) (PLA) and polyhydroxyalkanoates (PHAs) have gained a significant amount of attention in recent years. Nonetheless, some of the vital limitations to the broader use of these biopolymers are that they are less flexible and have less impact resistance when compared to petroleum-based plastics (e.g., polypropylene (PP), high-density polyethylene (HDPE) and polystyrene (PS)). Recent advances have shown that with appropriate modification methods—plasticizers and fillers, polymer blends and nanocomposites, such limitations of both polymers can be overcome. This work is meant to widen the applicability of both polymers by reviewing the available materials on these methods and their impacts with a focus on the mechanical properties. This literature investigation leads to the conclusion that both PLA and PHAs show strong candidacy in expanding their utilizations to potentially substitute petroleum-based plastics in various applications, including but not limited to, food, active packaging, surgical implants, dental, drug delivery, biomedical as well as antistatic and flame retardants applications.

## 1. Introduction

Petroleum based polymers have been helpful in meeting mankind’s requirements in variety of ways. Based on their composition, petroleum-based polymers can be very durable and disposable. However, the current combustion of fossil fuel has led to an alarming global climate change as a result of the release of carbon dioxide and greenhouse emissions. Wastes made from petroleum-based plastics such as garbage bags, food packaging containers and utensils are adding more burden to the environment. Furthermore, petroleum-based chemicals and solvents are also playing a role in reducing the quality of air. Therefore, finding new methods to secure a sustainable world development is an urgent need. Renewable biomaterials are considered as potential alternatives for petroleum-based products [1]. Polymers made from natural resins, for example shellac, gutta percha and amber, have a long history dating back to Roman times [2]. The official industrial application of natural polymer started in 1940s when Ford Motor Co. began experimenting with soybeans to produce sustainable automobiles [3]. Today, economic and environmental concerns are driving the trend for more utilizations of biopolymers. The current challenge is to develop the required methods necessary to make the revolution of biopolymers that are biodegradable and have renewable sources of feedstocks [4,5,6,7,8,9]. The level of materials and chemicals produced from biobased feedstocks has been continuously grown from 12% in 2010, to 18% in 2020 and is expected to reach to around 25% in 2030. It is expected that out of the $1.5 trillion worldwide chemical industry, two thirds will ultimately be based on renewable resources. A recent roadmap developed by United States Department of Energy and Department of Agriculture has specified a goal of 10% of chemical building blocks developed from agricultural resources by 2020 with ambitious plans to achieve a 50% increase by 2050 [4]. The research in the field of bioplastics has led to the discovery and developments of various new biobased products such as polyurethane products from soy oil, PLA from corn and PHAs from microorganisms [4,10,11,12].

Recent government policies that are focused on footprint reduction and conservation of energy are considered the driving force towards the use of sustainable and renewable bio-based polymers. For instance, single use plastics that are made from hard to recycle materials are to be banned in Canada by the end of 2021; for the aim to reach to zero plastic wastes by 2030 [13]. Thus, societies have started to switch to green resources to meet the demands of continuously increasing population in a way that does not affect the functioning ecosystems [14]. Among the most studied bio-based polymers to potentially substitute petroleum-based plastics are PLA and PHAs. This is due to their physical properties, barrier properties and stretchability which make them suitable for various applications. However, they suffer from some limitations which need to be overcome if these bioplastics are to compete with petroleum-based plastics. The objective of this work is to review the available materials on the modification’s methods of these two bioplastics and present their impacts with a focus on the mechanical properties. Therefore, the use of plasticizers, as well as the preparation of polymer blends and nanocomposites along with their applications have been reviewed. The combination of all these modifications’ methods in addition to their applications for both biopolymers is rare. The main aim of this review article is to widen the applicability of both biopolymers so as they can eventually replace petroleum-based plastics in new potential applications and therefore reduce the amount of waste and pollution.

### 1.1. PLA and Its Properties

**Figure 1:**
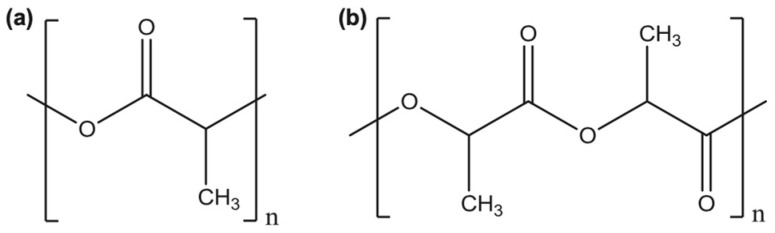
The chemical structure for: (**a**) poly(lactic acid) and (**b**) polylactide. Reprinted with permission from Elsevier, 2015 [15].

Figure 1 shows the chemical structure for poly(lactic acid) and polylactide [15]. PLA is an aliphatic linear poly(α-ester) or α-hydroxyalkanoic polyester that is acid-derived. PLA is produced through the ionic polymerization of lactide. Lactide is a cyclic compound that is produced when two molecules of lactic acid undergo dehydration–condensation. Fermentation of lactic acid from starch and other renewable resources by using different bacteria can also yield lactide. A direct way to obtain PLA is through polycondensation. Nonetheless, this process has two main drawbacks: firstly, the disposal of the solvent and secondly, this process results in low molecular weight polymers. Therefore, the most common technique used today for the production of L-lactide is the lactic acid’s two-stage polycondensation to yield an oligomer. This is then followed by depolymerization [15,16,17,18,19].

**Figure 2:**
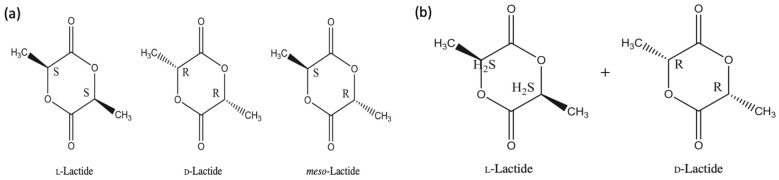
(**a**) Lactide’s three stereoisomeric forms and (**b**) rac-lactide. Reprinted with permission from Elsevier, 2015 [15].

Every molecule of lactic acid has one asymmetric carbon. Lactic acid has two optically active forms which are: L-lactide and D-lactide. Lactide has three isomeric forms as shown in Figure 2a. L-lactide consists of two molecules of L- lactic acid. Two molecules of D- lactic acid yield D-lactide. One molecule of L- lactic acid and another one of D- lactic acid give meso- lactide. L-lactide has a lower cost than D-lactide; this is because it occurs naturally [15,20]. Similar to L-lactide, meso-lactide is a cyclic diester that has two optically active atoms of carbon in the ring. It is considered as optically inactive because it has an optical R- and S-center. This form of lactide can be distinguished from the other forms by its melting temperature. Both: L-lactide and D-lactide have a melting temperature of 97 °C while the melting temperature for meso-lactide is 54 °C [15]. Yet, meso-lactide is not commercially available. L, D-lactide or rac-lactide is obtained by an equimolar mixture of L- and D-lactide as illustrated in Figure 2b. rac-lactide is produced through melting of equal quantities of L- and D-lactide. The melting temperature of meso- lactide is 129 °C. The most widely used isomeric forms of lactide are L-lactide and rac-lactide [15,20]. PLA can have many types such as: isotactic poly(L-lactide) (PLLA) and isotactic poly(D-lactide) (PDLA). PDLA is only available in small quantities and is very expensive. Other types are poly(meso-lactide) (mesoPLA), poly(rac-lactide) (PDLLA, racPLA), poly(L-lactide-*co*-D,L-lactide) (PLDLLA), poly(L-lactide-*co*-D-lactide) (PLDA), isotactic stereocomplex PLA (scPLA) and stereoblock PLA (sbPLA) as well as copolymers with other polymers [15,21].

**Figure 3:**
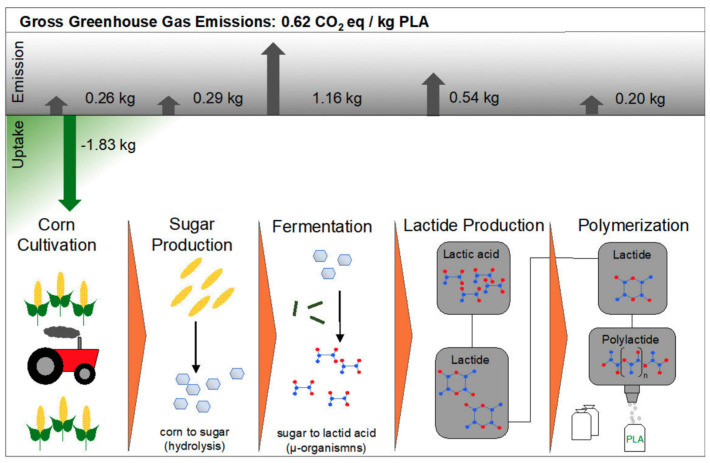
PLA’s production steps along with greenhouse uptake and emissions for 1 kg of PLA [23].

PLA’s properties are highly affected by the degree of crystallinity, molecular weight and the comonomer’s proportion. Glass transition temperature (T_g_), melting temperature (T_m_), Young’s modulus and tensile strength all increase at higher molecular weight, nonetheless, percentage elongation decreases. PLA is highly transparent, soluble in organic solvent and exhibits hydrophobic behavior. Different types of PLA show various mechanical properties as well as degradation rates [4,22]. For example, PLLA is a transparent and hard materials with glass transition temperature between 53–63 °C, melting temperature between 160–185 °C and crystallization temperature in the range of 100–120 °C. Due to its biocompatibility, natural renewable origin and its biodegradability, PLA has been gaining a lot of interest. Because it does not lead to a direct raise in the level of carbon dioxide, PLA can be considered as a low environmental impact thermoplastic [15,22]. Figure 3 shows the production steps of PLA along with greenhouse uptake and emissions for 1 kg of PLA [23]. The biodegradation of PLA is useful in terms of forming non-toxic products when PLA based products are composted after their life cycles [24]. Furthermore, PLA’s slow degradation rate can be beneficial for some applications to extend their shelf lives. Nonetheless, compared to poly(3-hydroxybutyrate) (P3HB or simply PHB) or Poly(ε-caprolactone) (PCL), the biodegradability of PLA is considerably low [15,25,26]. Although PLA exhibits low melt viscosity that is required for molds’ shaping, it suffers from some drawbacks. For instance, PLA exhibits low crystallization rate in long molding cycles and suffers from inferior gas properties. Moreover, PLA demonstrates poor mechanical properties (impact resistance and toughness) as well as thermal resistance when compared to other synthetic polymers. To overcome such limitations, PLA has been blended with other polymers. Furthermore, plasticizers and fillers have been incorporated with PLA. These methods in addition to preparation of PLA nanocomposites have been effective in making PLA more commercially viable. PLA is the mostly widely used biopolymer; as such, PLA is associated with various brand names for different applications as shown in Table 1 [15,27].

### 1.2. PHAs and Their Properties

**Figure 4:**
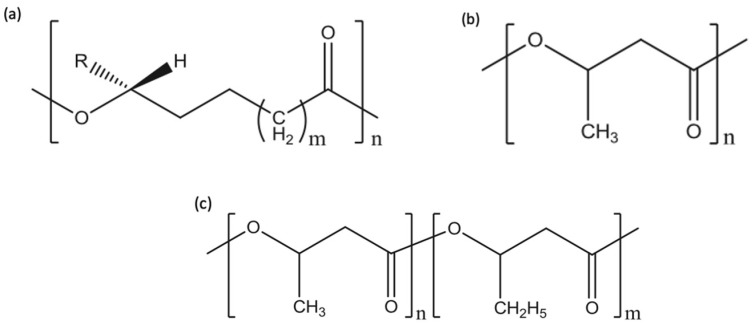
(**a**) The general chemical structure for PHAs, m ≥ 0, R = H, (un)substituted alkyl, (**b**) PHB’s chemical structure and (**c**) PHBV’s chemical structure. Reprinted with permission from Elsevier, 2015 [15].

PHAs are known as polyesters of 3-, 4-, 5- and 6- hydroxycarboxylic or hydoxyacids acids. The general chemical structure for PHAs is shown in Figure 4a. The side-alkyl chain’s length, the one additional methyl group at carbon atoms between the carboxyl group and hydroxyl group, the hydroxyl group’s position relative to the carboxyl group and the large variety of substituents in the side chains all play a role in differentiating between the different types of hydroxyalkanoic acids [30]. This family of biopolymer is produced by various bacteria as intercellular carbon as well as energy storage materials [31]. PHAs can be found as scattered granules inside the cells of bacteria and may take up to 90% of bacteria’s dry cell weight. Because they are produced in a natural way via soil bacteria, PHAs degrade when exposed to similar bacteria in compost, marine or soil. Biodegradation initiates when PHAs start to break down to hydroxy acid monomeric units via the different microorganisms on the surface of the biopolymer. The microorganisms then benefit from these hydroxy acid units by using them as sources of carbon for growth. This family of polymers can be also produced chemically [30,32,33,34,35,36]. The monomers for PHAs can range between a three carbon atoms compound (3-hydroxypropionate) to a compound with 14 carbon atoms (3-hydroxytet-radecanoate). Based on the number of carbon atoms, this family of biopolymers can be classified to short chain length PHAs (scl-PHA) and PHAs with medium chain length (mcl-PHA). scl-PHA consists of 3–5 carbon atoms, while mcl-PHA contains 6–14 carbon atoms. Due to PHAs’ compositional diversity, PHAs can exhibit different physical properties [37].

Today, many bacterial fermentations derived PHAs are commercially available, this include PHB, poly(3-hydroxybutyrate-*co*-3-hydroxyvalerate) (PHBVor PHBHV), poly(3-hydroxybutyrate-*co*-3-hy-droxyhexanoate) (PHBHH_x_) and poly(3-hydroxybutyrate-*co*-4-hydroxybutyrate) (P3HB4HB). The chemical structures of PHB and PHBV are shown in Figure 4b,c, respectively. Generally, scl-PHA such as PHB are brittle. As the length of monomer chain increases such as Poly(3-hydroxyoctanoate) (P3HO), the material exhibits more flexibility [38]. Because of their flexible properties, PHAs can ultimately substitute polyethylene (PE), PS and PP, which are the major polymers in today’s polymer market [39]. Using thermal manufacturing processes such as injection molding, PHAs can be processed well. PHAs can be used in many applications such as, garbage bags, food packaging, diapers, as well as medical equipment [31,40]. This family of biopolymers have been widely studied to investigate their use in biomedical applications. PHAs have been also used in surgical sutures, lubricating powders, controlled release, bone fracture fixation plates, tissue scaffolds, wound dressings and surgical implants. Table 2 shows some of the commercial PHAs along with their applications [15,41,42].

**Figure 5:**
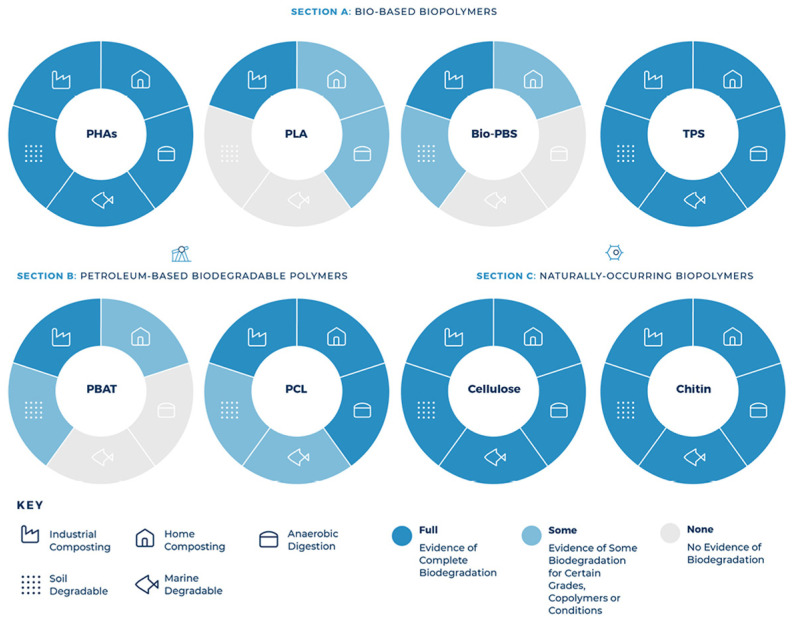
The end-of-life pathways for some plastics alternatives [51].

PHB, is the most simple and widely used member of the PHAs family [43]. PHB is synthesized and kept within the cells of different microorganisms as a source of energy [44]. PHB’s production is usually done in two steps. The first step is fermentation in which various microorganisms store PHB inside their cells after they metabolize the available sugar in the medium. The second step includes extraction and purification of the PHB accumulated inside the microorganisms. PHB is stereoregular structure homopolymer that exhibits high degree of crystallinity. PHB is a stiff and brittle polymer with low melt viscosity and limited processing window. Therefore, the utilization of PHB in various applications is narrow [45]. Yet, many techniques have been used to enhance the ductility of PHB such as: the use of plasticizers, additives, nucleating agents, copolymerization of 3-hydroxybutyrate with 3-hydroxyvalerate (HV) and modification of the processing conditions. PHB has been used in many applications such as: packaging, agriculture for the release of fertilizers and in biomedical devices to regulate the release of drugs. Moreover, it has been used with non-biodegradable polymers as a bio filler to accelerate degradation [15]. The insertion of HV units into PHB biopolymer’s backbone through fermentation results in PHBV or PHBHV, which is one of the most promising member in this family of biopolymer [46]. Compared to PHB, PHBV exhibits better toughness and flexibility as well as lower processing temperatures. Currently, PHBV with 15% HV content is commercially available. PHBV with higher HV content is very expensive to produce and therefore not commercially viable [47]. An increase in water permeability, glass transition and melting temperatures, as well as tensile strength is resulted from lowering the content of HV. Yet, percentage elongation at break and impact resistance decrease [48,49]. Due to its similar properties with PP, PHBV is considered as a promising green material. The percentage elongation of PHB and PHBV ranges from around 4 to 42% [15,50]. PHB and PHBV have been used in wound dressing, scaffolds, regeneration of tissue, blood vessels and food packaging [15]. Both PHB and PHBV exhibit some undesirable properties. For example, PHB demonstrates thermal instability, at the same time, both of PHB and PHBV have slow crystallization rates and flow properties. This makes it challenging to process these biopolymers. While processing PHBV, it exhibits a sticky behavior for a long period of time and might stick to itself while processing it into films. As shown in Figure 5, PLA and PHAs are biobased, biodegradable and ecologically friendly polymers with good strength and stiffness. They are intended to replace petroleum-based plastics in various applications. However, they suffer from high brittleness which hinders their utilization in many other potential applications [15].

## 2. PLA’s Modifications

PLA has been reported as one of the most commonly used biodegradable polymers. It has been successfully used in various applications such as food packaging and biomedical devices. Due to its several advantages, PLA is considered as a tempting substitute for petroleum-based nonbiodegradable polymers in such applications. Some of the advantages of PLA include biodegradability, recyclability, biocompatibility and renewable sources (e.g., corn, wheat and rice) [52]. Due to PLA’s nontoxicity and carcinogen-free interaction with human tissues, many biomedical industries have switched their eyes on PLA. Data from different implantation surgeries shows that there has been an absence of any kind of toxic products produced from the degradation of implanted PLA devices. In addition, such produced products did not interfere with the healing process of tissues. Nonetheless, PLA’s use and implementation in food packaging and biomedical applications has been limited due to some of its drawbacks. Some of the important drawbacks of PLA are related to its poor mechanical properties such as, its brittleness, low modulus of elasticity, low percentage elongation at break and low tensile strength [52].

Table 3 shows the mechanical properties along with physical and thermal properties of some PLA developed by NatureWorks LLC [53,54]. Depending on various parameters such as: polymer structure, material formulation (blends, plasticizers, composites, etc.), orientation, crystallinity and molecular weight, the mechanical properties of commercial PLA can be diverse, ranging from elastic soft to stiff, high-strength materials. PLA exhibits some similarity with polystyrene (PS) in which it is a brittle material with low elongation at break and impact strength. However, its Young’s modulus and tensile strength are comparable with polyethylene terephthalate (PET). A comparison between the mechanical properties of PLLA, PS and PET is shown in Table 4. Due to its poor toughness, the use of PLA in applications that requires plastic deformation at higher stress levels has been avoided. For instance, the implementation of PLA in bone surgery as screws and fracture fixation plates has been substantially narrow due to the lack of PLA’s high plastic deformation behavior under high stress level condition [55]. The low stiffness of PLA’s implant devices can hinder the healing process and cause excessive bone motion. This has opened the door to develop various modification techniques to improve the mechanical properties of PLA, specifically its toughness [56].

### 2.1. Plasticizers’ Effect

PLA is classified as a glassy polymer with a poor elongation at break that is around 10% only. For this reason, various biodegradable and non-biodegradable plasticizers have been used in order to increase its ductility, improve it processability and increase its thermal stability (glass transition temperature) [57]. Such enhancements in the properties of PLA can be achieved through controlling the plasticizers’ polarity, end groups and molecular weight. One of the effective monomers for plasticizing PLA is lactide. For example, PLA’s elongation at the break can increase up to 288% when 17.3 wt.% of lactide is added to PLA. Nonetheless, lactide suffers from losses and fast migration [58]. Therefore, and since high molecular weight plasticizers do not have a high potential to migrate, they remain the preferable choice.

Different studies in literature have investigated the use of poly(ethylene glycol) (PEG) with different molecular weights as plasticizers for PLA to improve its mechanical properties. In one study, Jacobsen and Fritz [59] studied the effect of PEG with molecular weight 1500 g/mol (PEG1500) on the mechanical properties of PLA. When PEG or glucose monoester were added, there was an increase in the elongation at the break with increasing the amount of plasticizer. This was not the case when partial fatty acid esters were used as a plasticizer. This variation is attributed to the fact that activation cells that led to crack formation were triggered by the finely distributed partial fatty acid ester. According to the study, PEG is the best plasticizer to be used for enhancing the elongation of PLA. For instance, the addition of 10 wt.%. of PEG to PLA can result in an enhancement of the percentage elongation of up to 180%. Results of the impact resistance suggest that high amount (10 wt.% concentration) of PEG can lead to a significant increase in the impact resistance to a point that no break was observed. Nonetheless, the addition of any of the small amounts of PEG, glucose monoester at any concentration or any concentration of partial fatty acid ester resulted in a drop in the impact resistance. This decrease in the impact resistance can be explained by disturbance produced by the plasticizer particles inside the PLA matrix. This disturbance has prevented the sliding of chains to absorb shock energy [59]. The elongation at break was observed to increase with higher concentrations of PEG with molecular weight 400 g/mol or oligomeric lactic acid; however, when 20 wt.% of either of the two plasticizers was added, the highest drops in the modulus of elasticity of 53% and 65% were reported, respectively. At 20 wt.% oligomeric lactic acid, a maximum elongation at break of 200% was reported [60]. In case of PEG with molecular weight of 10,000 g/mol, 20 wt.% of PEG was needed to result in a significant increase in PLA’s percentage elongation. Yet, the same change was achieved by incorporating 10 wt.% of PEG with low molecular weight (400 g/mol). Nonetheless, this improvement was at the expense of Young’s modulus and tensile strength [61]. For PEG with molecular weights between 200 g/mol and 1000 g/mol, the optimum elongation at break was reported at 20 wt.% [62]. When PEG higher than 20 wt.% was added to the PLA, the modulus of elasticity was found to decrease drastically. It was also found that PLA’s physicomechanical properties were not weakened when PEG with molecular weight of 200 g/mol was blended with PLA at a concentration of 10 wt.%. That was also the case when PEG with molecular weights 400 g/mol and 1000 g/mol were blended with PLA at concentrations of 20 wt.% and 30 wt.%, respectively. Due to the lack of cohesion between the separate phases, the blend exabits a brittle behavior when higher plasticizer content was added. Therefore, the efficiency of the plasticizer is linked to the molecular level miscibility, which is higher for PEG than for other plasticizers used in the same study (acetyl glycerol monolaurate (AGM), dibutyl sebacate (DBS) and poly(1,3-butanediol) (PBOH)). The results suggest that the most effective plasticized formulations that give the best mechanical properties are AGM, PBOH and DBS at concentrations of 20–30%, respectively [62].

Polypropylene glycol (PPG) was reported as an effective plasticizer for PLA. The facts that PPG exhibits low glass transition temperature, does not crystallize in addition to its miscibility with PLA, make PPG a tempting plasticizer to blend with PLA. Mechanical properties suggest that using 12.5 wt.% of lower molecular weight PPG demonstrates the best performance. This is because it gives the highest increase in the elongation at break with the minimum decrease in tensile strength [63].

Nijenhuis et al. [64] found that enhancement of PLA’s properties can be obtained using polymeric plasticizers. In their study they have successfully added high molecular weight poly(ethylene oxide) (PEO) to PLLA to enhance its elongation at break. The effect of high molecular weight PEO on the PLLA’s elongation at break was mostly prominent at high concentrations exceeding 10 wt.%. For instance, the elongation of PLLA reached up to 500% when 20 wt.% of PEO was added. Nonetheless, when PEO at a concentration of 20 wt.% was used, a reduction in the tensile strength from 58 MPa for the neat PLLA to 24 MPa was observed [64].

Labrecque et al. [65] investigated the use of Citrate esters obtained naturally from citric acid as a potential plasticizer for PLA. The tensile strength significantly dropped to around 50% when the plasticizers were used. The deterioration was higher at larger concentrations. At relatively lower concentrations such as 10 wt.%, there was no major change in the elongation at break; however, when higher concentrations that are more than 20 wt.% were added, a significant increase in the percentage elongation was noticed. When 30 wt.% of triethyl citrate was added, the highest elongation at break value (610%) was reported. Unfortunately, this was accompanied with a major loss in the tensile strength [65].

The feasibility of adding poly(ethylene-*co*-vinyl acetate) (EVA) to PLLA as a plasticizer was studied by Yoon et al. [66]. Results showed a slight increase in the elongation at break for the PLLA/EVA blend when EVA up to 70 wt.% was added. However, a significant enhancement in the elongation at break was reported at 90 wt.% EVA at which a maximum elongation of 209% was reported. On the other hand, both the tensile strength and modulus of elasticity decreased rapidly. This was followed by a more gradual drop as the concentration of EVA increased [66].

Ren et al. [67] have used triacetin and oligomeric poly(1,3-butylene glycol adipate) with low molecular weight in an attempt to plasticize PLA. Results suggest that the resulted blend had a positive impact on improving PLA’s elastic properties; however, that was accompanied by a reduction in the tensile strength. The blends were brittle at plasticizer’s concentrations less than 5 wt.% but exhibited a ductile behavior at concentrations higher than 5 wt.% [67].

In another study [68], conventional and reactive extrusion was used to blend PLA with limonene (LM) or myrcene (My) bio-based plasticizers. Results showed that both plasticizers were efficient in improving the impact strength and ultimate tensile strength of PLA. This was also accompanied with a reduction in T_g_. The incorporation of a free radical initiator throughout the extrusion of PLA/LM was beneficial for the mechanical properties. The probable formation of local crosslinked regions in the PLA matrix improved the matrix’s ultimate tensile strength, yield strength and crystallinity in comparison to the non-reactive PLA/LM blend. However, other properties were retained [68].

The utilization of ozonized soybean oil (OSBO) as a biobased plasticizer for PLA was also investigated [69]. Plasticized PLA samples were made by compounding. OSBO contents in the range of 0 to 15% was added to PLA and the impacts on mechanical and thermal properties were evaluated and studied. Results showed that after the ozonolysis reaction, formation of hydroxyl groups in OSBO as well as an increase in ester groups were observed. As the content of OSBO increased, the impact strength and percentage elongation at break also increased, yet the tensile strength decreased. PLA’s T_g_, T_m_ and crystallization temperatures continuously decreased as a function of OSBO content. PLA’s crystallinity was also improved due to the presence of OSBO. In summary, at low content, OSBO acted as a plasticizer for PLA; however, at 15% OSBO, there was a formation of fine oil droplets which acted as an impact absorber by energy dissipation [69].

Dominguez-Candela and co-authors [70] have reported a new biobased PLA plasticizer derived from Epoxidized Chia Seed Oil (ECO). PLA with various contents of ECO (0–10 wt.%) was prepared using melt extrusion. Results showed an improvement by 700% in the percentage elongation at break at 10 wt.% ECO. Up to 5 wt.% ECO, plasticized PLA was disintegrated under composting conditions with no delays. Results of the migration tests indicated a very low migration level (lower than 0.11 wt.%), which is of much interest to the packaging industry [70].

In another investigation [71], the use of dibutylmaleate (DBM) and dibutylfumarate (DBF) as biodegradable plasticizers to PLA was studied. Thermal and mechanical properties of plasticized PLA were investigated. Results showed that DBF had a more pronounced plasticization effect exhibiting lower glass transition temperature, yield strength, viscoelastic properties, modulus of elasticity and higher elongation at break. This was attributed to the end-to-end distance of the plasticizer’s molecules. The incorporation of 12 wt.% DBF to PLA led to an increase in the elongation at break from 1.3% for neat PLA to around 210.00% [71].

A summary of the various plasticizers reported in literature along with their effects on PLA’s mechanical properties at various concentrations is shown in Table 5.

### 2.2. Impact Modifiers’ Effect

Various impact modifiers can be incorporated into PLA to lower its brittleness while preserving its stiffness. In one study [72], 10 wt.% of Biomax Strong (BS) 100 impact modifier was added into PLA. Results showed an increase in its tensile properties and percentage elongation. Moreover, when 10 wt.% of BS was added, plasticized Cloisite 25A/PLA composites maintained their strength and rigidity while exhibited good ductility [72].

In another study [73], a substantial enhancement in the elongation at break and the notched impact strength of PLA was reported as a result of increasing the content of BS impact modifier up to 50 wt.%. Nonetheless, there was a reduction in the Young’s modulus and yield stress of PLA with increasing the amount of BS impact modifier. That was attributed to BS impact modifier’s toughening effect which reduced PLA’s crystallinity by improving PLA matrix’s plastic deformation [73].

The effect of adding Paraloid BPM-515 impact modifier into PLA/talc composites was also investigated. In one study [74], it was found that the toughness of the composite increased as a result of the successful incorporation of 1.8 wt.% of Paraloid BPM-515 impact modifier. This was attributed to the improved compatibility between the talc fillers and the PLA matrix after the addition of the impact modifier [74].

Diaz et al. [75] were able to incorporate Paraloid BPM-515 impact modifier into PLA. Results showed a rapid improvement in the impact strength and a slight increase in the elongation at break of PLA due to the addition of the impact modifier [75].

The synthetization of two transparent impact modifiers—poly(butadiene-*co*-methyl methacrylate-*co*-butyl methacry- late-*co*-butyl acrylate-*co*-hydroxyethyl methacrylate) (known as BMBH copolymer) and poly(butadiene-*co*-lactide-*co*-methyl methacrylate-*co*- butyl methacrylate) (known as BLMB copolymer) as PLA impact modifiers was reported by Choochottiros and Chin [76]. The results showed an improvement in the impact strength and toughness while maintaining the clarity of PLA [76].

Nonetheless, most of PLA’s impact modifiers available today are nonbiodegradable. Moreover, they are usually used at a concentration of 10 wt.% for various applications in the industry. Therefore, for applications where the biodegradation of PLA is vital, different studies suggested the use of biodegradable polymers (e.g., PCL, poly(butylenes succinate), poly(propylene carbonate), poly(butylenes adipate-*co*-terephthalate), poly(tetramethylene adipate-*co*-terephthalate) and poly(*p*-dioxanone) (PPD)) as biodegradable impact modifiers for PLA applications [72,77,78]. In their study, Odent et al. [79] found that the addition of poly(ε-caprolactone-*co*-δ-valerolactone) as a biodegradable impact modifier improved PLA’s toughness while maintaining its transparency [79]. Table 6 provides a summary of various impact modifiers that are specifically designed for PLA applications.

### 2.3. Belnding’s Effect

An alternative effective approach that results in new materials with required properties is polymer blending. This approach depends on modifying the available polymer rather than synthesizing entirely new polymers. The ability to blend various polymers and at the same time conserve their distinct properties in the final blend is a tempted and cheaper way for producing new polymers with desirable properties. Preparing blends usually involve the use of twin-screw extruders. To produce a blend with desirable properties, different factors must be taking into consideration. For example, the barrel temperature must be adjusted to be above the T_g_ of that of an amorphous polymer or above the T_m_ of a semi crystalline polymer. This is crucial to control the viscosity so as to result in an optimal dispersion. The lower limit for PLA blends should be around 180 °C. Thermal degradation of PLA is possible at high temperatures; therefore, polymers that are processed at relatively extreme processing temperatures, that is higher than 270 °C are not preferable candidates for PLA blends. The desired properties resulted from blending of one or more polymers do not always come without a cost. When dealing with miscible blends, one of the biggest challenges is to obtain a good interfacial adhesion among the blending phases. This can directly influence the morphology and, consequently, the mechanical and physical properties. Another issue arises if the added polymer and PLA are not very compatible. In this case, extra subsequent work is required to enhance the compatibility. In case of poor interfacial adhesion, PLA blend can suffer from embrittlement. Furthermore, a significant change in phases’ morphology can take place, based on product’s design as well as the processing conditions. Another issue can occur when blending PLA with non-biodegradable polymers as this can affect the composability of PLA [4,85,86]. Blending more than two biopolymers does not necessarily yield a biodegradable blend even if one of the blended polymers is biodegradable. The selection of polymer blending partners depends on the desired properties of the final blend. For example, mechanical properties, such as stiffness and toughness, whether the blend should be biodegradable, the rate of biodegradability, the desired chemical and physical properties, crystallinity and miscibility, all play a role in the selection of blending partner. Stiff polymers have higher crystallinity and are brittle while flexible polymers are more amorphous. Hence, when a tough flexible biopolymer is added to a brittle biopolymer, this will increase the impact resistance while at the same time reduce the strength and modulus. Optimized properties and performance are believed to be achieved by blending brittle biopolymers with flexible biopolymers. In biodegradable materials, the two most important points in producing functional biopolymer blends are, (1) the compatibility or miscibility of the blend and (2) the whole biodegradability of the blend and its composition. Polymer blending can be divided into three categories [86]:

Heterogeneous or immiscible polymer blends: In such blends, the polymers exist in separate phases and the respective glass transition temperatures are detected.

Compatible polymer blends: Such blends are immiscible and demonstrate uniform macroscopical physical properties. This can be attributed to the robust interactions between the polymers’ component.

Homogeneous or Miscible polymer blends: This type of blends are usually made from polymers that have similar chemical structures. This will lead to a single-phase structure polymer blend with only one glass transition temperature.

#### 2.3.1. PLA/PHAs Blends

PHAs are biodegradable linear polyesters that are obtained by various microorganisms. One of the most common and simplest form of PHAs family is PHB. Because PHAs are produced from renewable natural resources, blends of PHAs/PLA are expected to be completely biodegradable. The miscibility of PLA/PHB blends depends on the PLA’s molecular weight. Using a lower molecular weight PLA usually leads to a highly miscible PLA/PHB blend [87,88,89,90]. Different studies have investigated the mechanical properties of PHAs/PLA blends.

Blending PLLA with PHBV was investigated by Iannace et al. [91]. The blend was prepared by solution casting of chloroform at room temperature. For the blends with 20 wt.% and 40 wt.% PHBV, results showed a minor increase in the elongation at break. As more PHBV content was used, Young’s modulus and tensile strength both reduced. These results were supported by a drop in the crystallinity of the PLLA phase when more PHBV was incorporated [91].

In a similar work [92], the mechanical properties for PLLA/PHBV blends were reported. The study confirmed the trend of the elastic modulus reported from the previous study [91]; however, the tensile strength of PLLA/PHBV blends were lower. The reason behind that was that Iannace et al. [91], obtained dense PLLA films only, while a porous PLLA film was prepared in this study [92].

Yoon et al. [93] investigated the mechanical properties of PLLA/PHB blends after incorporating various types and amounts of compatibilizers. PLLA/PHB blends with a concentration of 50/50 wt.% were blended in 3 wt.% chloroform. Poly(vinyl acetate) (PVAc), PLLA-PEG-PLLA triblock copolymer and PEG PLLA diblock copolymer at 2 wt.% and 5 wt.% were used as compatibilizers. When a compatibilizer was used, all the blends reported improvements in the tensile toughness and percentage elongation for both compositions when compared to the PLLA/PHB blend without a compatibilizer. Nevertheless, when compared with an un-compatibilized PLLA/PHB blend, the modulus of elasticity was lower for all the blends at various amounts of compatibilizer. The values of tensile strength varied according to the type and composition of the compatibilizers. Tensile strength was reduced in both blends with 5 wt.% of diblock and triblock copolymers as well as the PVAc as compatibilizers with respect to the un-compatibilized PLLA/PHB blend. However, a maximum tensile strength of 69.8 Mpa was reported for the blend of 2 wt.% PLLA-PEG-PLLA triblock copolymer, this was followed by a tensile strength value of 65.5 Mpa that was reported for the 2 wt.% PEGPLLA diblock copolymer. PLLA/PHB blend with 2 wt.% of PLLA-PEG-PLLA triblock copolymer reported the best results in terms of percentage elongation, tensile strength and toughness. Moreover, the mechanical properties were better than those of the un-compatibilized PLLA/PHB blend, yet, the modulus of elasticity exhibited a minor reduction [93].

In another work, PLA/PHA blends were prepared by Takagi et al. [94] at various compositions. PLA was blended with PHA as well as with functionalized PHA with 30% epoxy group in its side chains (ePHA). For all compositions, PLA/PHA and PLA/ePHA blends exhibited lower tensile strengths than that of the neat PLA. On the other hand, as the composition of PHA or ePHA increased, Charpy impact strength for both blends increased as well and were higher than that for neat PLA. PLA/ePHA blends reported higher tensile strength and Charpy impact strength compared to the PLA/PHA blends. That was explained by the inserted epoxy side group of ePHA which enhanced the blend’s compatibility [94].

Noda et al. [95] were able to prepare PLA/PHA blends via melt mixing using a single-screw extruder. The study used Nodax™ which is poly(3-hydroxybutyrate)-*co*-(3-hydroxyalkanote) in the investigation. When 10 wt.% Nodax™ was added, the blend’s toughness improved dramatically. The tensile energy was 10 times more than that of the neat PLA. However, Nodax™’s positive effect was only noticeable up to around 20 wt.%. Further addition of Nodax™ lowered the blend’s toughness back to the value of neat PLA. That was explained by the fact that at Nodax™ content less than 20 wt.%, the copolymers dispersed in a fine way in the PLA matrix. The PHA portion of the blend stayed predominantly in a liquid-like amorphous state, therefore crystallization was hindered. The toughness and ductility of the blend were then resulted from the reduced crystallinity [95].

Schreck and Hillmyer [96] reported a similar study of PLLA/Nodax™ blend. A 75 rpm mixer at 190 °C was used for 15 min to compound the blends. The compositions of Nodax™ used in the study was from 0 to 25 wt.%. Similar to the trend reported by Noda et al. [95], enhancements in toughness were reported for the blends for up to 20 wt.% Nodax™. Neat PLLA’s impact strength was around 22 J/m whereas the highest impact strength value was 44 J/m which was reported for the blend with 15 wt.% Nodax™. In an attempt to enhance the binary blend properties, the study also investigated the impact of ternary blends of 81/14 wt.% PLLA/Nodax™ and 5 wt.% oligoNodax-b-poly(L-lactide) diblock copolymers as compatibilizers. There was no reported improvement in toughness with the incorporation of 5 wt.% oligoNodax-b-poly(L-lactide). This is attributed to the weak interfacial adhesion at the particle-matrix interface as a result of low entanglement of oligoNodax with Nodax™, which accordingly lowered the tendency to dissipate and deform impact loads [96].

In another study [43], a melt compound was used to come up with different PLA/PHB-based blends with different weight ratios (100/0, 75/25, 50/50, 25/75, 0/100). Results of the study showed that there was substantial improvement in the tensile properties of the blend in the case of PLA (75 wt.%) to PHB (25 wt.%) blends. This was attributed to the presence of PHB crystals, which acted as a filler and nucleating agent in the polymeric matrix of PLA [43].

Bartczak et al. [97] were able to improve PLA’s impact strength and drawability by adding 20 wt% of atactic PHB (a-PHB). Due to the partial miscibility of PLA and PHB, the melting and cold crystallization temperatures of PLA have slightly changed. Results suggest that as the concentration of a-PHB in the blend increased, PLA’s glass transition temperature was reduced. Using compression molding or extrusion technique, amorphous foils for food packaging were developed from the PLA/a-PHB blend. As the a-PHB content increased, the yield stress as well as the elastic modulus exhibited a slight drop, yet this was accompanied by an increase in the ultimate strain increased. This was explained by the thick aggregations of diffused crazes observed in PLA. It is believed that such crazes united to form deformation bands and macroscopic neck. Nonetheless, there was an increase in the tensile impact resistance of the thin film from 50 kJ/m^2^ in case of neat PLA to around 118 kJ/m^2^ for the PLA/a-PHB (80/20 wt.%) blends. The yield strength decreased with increasing the content of a-PHB [97].

Using melt blending technique, Nanda et al. [98] were able to successfully fabricate opaque PHBV/PLA blends for the aim of enhancing PHBV’s mechanical and thermal properties. As per the study, there was a 250% and 148% increase in the elongation at break values for virgin PLA and PHBV, respectively [98]. PHBHH_x_ which is an mcl-PHA demonstrates better mechanical and thermal properties than the scl-PHAs. Some studies have reported a substantial improvement in the mechanical properties of PLA/PHBHH_x_ blends after the incorporation of 20 wt.% of the later [95,99,100].

Another study [101] reported a substantial improvement in the toughness of PLA/PHBHH_x_ blends due to the presence of 10 wt.% PHBHH_x_, yet the blend reported to be incompatible [101].

In a similar investigation, Lim et al. [102] were able to fabricate PLA/PHBHH_x_ blends. As the amount of PHBHH_x_ increased, a drop in the PLA’s crystallization was noticed. The investigation suggested that the ductility and toughness of PLA/PHBHH_x_ blends for food packaging applications can be improved through the incorporation small quantities of PHBHH_x_ to PLA. This is because for small quantities of PHBHH_x_, the tendency of aggregation was found to be insignificant [102].

In another study [103], melt blending was used to come up with a transparent multifunctional PLA, ATBC, cellulose nanocrystals (CNCs), modified CNCs and PHB flexible film for food packaging applications. The developed film demonstrated improved crystallinity, better stretchability, outstanding oxygen barrier properties and enhanced percentage elongation at break. Furthermore, the degradation was improved due to the incorporation of both the plasticizer and CNCs [103].

Moreover, different studies in literature have investigated the impact of different types of plasticizers and their quantity on the resulting blends’ mechanical and thermal properties as well as degradation rate [104,105,106,107,108].

PLA/PHB blends (75/25 wt.%) with the incorporation of Lapol 108 as a plasticizer at two different concentrations (5 wt.% and 7 wt.% per 100 parts of the blends) were produced by Abdelwahab et al. [108]. There was no sign of phase separation in the produced PLA/PHB blend. The blends showed a fine distribution of the main ingredients. Additionally, the miscibility of PLA and PHB with plasticizer was examined using a Differential Scanning Calorimetry (DSC). The DSC curves showed a single glass transition temperature value that exhibited a drop when the plasticizer’s quantity increased. Yet, there was no significant variations in the blend’s melting temperature and thermal stability for a certain quantity of the plasticizer. However, the PLA/PHB blend’s elongation at break was enhanced due to the incorporation of the plasticizer [108].

Sofiane et al. [109] investigated the printability of PLA/PHA blend from physical and structural aspects. The study found that samples printed at higher temperatures and experienced high cooling rates were reported to be more ductile than those printed at low temperatures. This is attributed to the lower degree of crystallinity at high cooling rates. The study has also reported a low amount of porosity (less than 6%) in 3D-printed PLA/PHA blends via Fused Deposition Modeling (FDM). Furthermore, there was a positive impact of the printing temperatures on the tensile performance, density and porosity content. At low printing temperatures, the drop of tensile properties was more pronounced for the percentage elongation than for modulus of elasticity and tensile strength. According to the study, 3D-printed PLA/PHA blends are promising candidates for medical and pharmacological applications [109].

Recently, Olejnik and co-authors [110] blended PLA with PHB at various mixing mass ratios with the aid of an extruder. Results of the investigation showed that there was a drop in the T_g_ due to the incorporation of PHB to PLA. Results of the mechanical analysis also showed a drop of the ultimate tensile strength and tensile strength at break as a function of PHB content; however, low PHB content has led to material enhancement. Percentage elongation at break was found to raise in an exponential way as a function of the PHB’s content [110].

Table 7 shows the impact of different PLA/PHAs blends at various concentrations on the mechanical properties.

#### 2.3.2. PLA/PCL Blends

Due to its rubbery characteristics as well as its high elongation at break (roughly 600%), Polycaprolactone (PCL) is considered as a good candidate for toughening PLA [77]. PCL is also a degradable polyester, meaning that blending it with PLA can result in a completely degradable material. Many studies in literature have reported that PLA/PCL blends can result in enhanced elongation at break; however, this is usually accompanied with a reduction in modulus of elasticity and tensile strength.

Hiljanen-Vainio et al. [111] showed that blending 20 wt.% of PCL with PLLA resulted in a lower Young’s modulus, tensile strength and shear strength, However, the elongation at break increased from 1.6% for neat PLLA to 9.6%. On the other hand, blending of the elastic poly(ε-caprolactone/L-lactide) (PCL/L-LA) copolymer with PLLA substantially increased the elongation at break to more than 100% compared to both, neat PLLA and the binary blend. Yield deformation was observed for PLA with 5, 10 and 20 wt.% of PCL/L-LA copolymer. A tough rubber-like behavior was reported when the blend contained 30 wt.% of PCL/L-LA copolymer. Initially, the impact strength of PLLA was very poor, however, when 20 wt.% of PCL/L-LA copolymer was added, a quadruple enhancement in the impact strength was obtained [111].

The tensile properties for PLA/PCL blend films were studied by Tsuji and Ikada [112]. The blend films were prepared with a solution casting method using methylene chloride as a solvent. Adding 15 wt.% PCL to PLA resulted in increasing the elongation at break; however, the calculated standard deviation obtained was quite high (250% ± 200%) [112].

The elongation at break for PLA/PCL blend was investigated by Wang et al. [77]. Results suggested that the elongation at break for reactive blends of PLA/PCL using triphenyl phosphite as a catalyst increased substantially when compared to neat PLA at certain compositions (PLA/PCL = 80/20 or 20/80). Therefore, the study indicates that reactive blending is a promising technique to enhance the toughness and elongation of PLA. The elongation increased to 127% compared to 28% for the nonreactive binary blend [77].

Maglio et al. [113] reported an enhancement in both the percentage elongation and the notched Charpy impact strength in PLLA/PCL 70/30 wt.% blends compatibilized with PLLA-PCL-PLLA triblock copolymer [113].

PLA/PCL blends were examined by Broz et al. [78]. Only for a PCL content higher than 60 wt.%, a significant increase in the elongation was observed, nonetheless, this was accompanied with a drastic drop in Young’s modulus and tensile strength [78].

The addition of diblock copolymer of PLLA-PCL to PLLA/PCL blends was studied by Tsuji et al. [114]. At X_PLLA_ (X_PLLA_ = weight of PLLA/(weight of PLLA and PCL)) of 0.5–0.8, blends’ tensile strengths and modulus of elasticities were enhanced due to the addition of the copolymer. Moreover, the elongation at break was also improved for all values of XP_LLA_. These findings indicate that PLLA-CL was miscible with PLLA and PCL, and that the dissolved PLLA-CL in PLLA-rich and PCL-rich phases improved the compatibility between phases [114].

In another study [115], dicumyl peroxide (DCP) was used as a cross-linker in PLA/PCL reactive blend. DCP was used to enhance the elongation at break of the blends. The study reported that the optimum blend ratio of the PLA/PCL blend was 70/30. The elongation at break reached a peak value when low DCP concentrations (around 0.2 phr) was used. In addition, at low DCP content, tensile testing showed yield point and ductile behavior. For the optimum composition, there was a substantial increase in the impact strength [115].

Blends of PLA and a copolymer of caprolactone (CL) as well as trimethylene carbonate (TMC) has been investigated by Grijpma et al. [116]. When 20 wt.% copolymer was incorporated, the notched Izod impact strength increased from 40 J/m to a peak value of 520 J/m. Nonetheless, the same concentration of rubber phase resulted in no enhancement in the notched Izod impact strength in case of homopolymer poly(TMC) and PLA blends [116].

Blends of PLA homopolymer with poly(trimethylene carbonate) [poly (TMC)] copolymers were also studied [117]. In an unnotched impact test, there was no breakage of the blend samples with 21 wt.% of the block of poly(TMC) in PLA. The study also examined the effect of diblock copolymers of L-lactide and CL blended with PLA on the mechanical properties. When 20 wt.% of diblock copolymer was used, there was an increase from 5 to 50 kJ/m^2^ in the blend’s unnotched impact strength [117].

Surfactant has also the potential to improve the elongation at break of PLA when added at low quantity. However, such an improvement is accompanied by a drop in both the tensile strength and Young’s modulus [118].

According to a study reported by Maglio et al. [113], when a small amount (round 4 wt.%) of PLA-PCL-PLA triblock copolymer was added to PLA/PCL blend with a concentration of 70/30 wt.%, there was an enhancement in the dispersion of PCL. Moreover, an improvement in the resulted blend’s ductility was observed. The percentage elongation increased to 53% for the ternary blend from 2% for a PLA/PCL (70:30) blend. This was attributed to the dispersion of PCL domains which after the incorporation of 4 wt.% triblock copolymers were observed to decrease from 10 to 4 μm [113].

PLA/PCL blends with different PCL molecular weights were prepared by Hasook et al. [119]. Out of all the blends, the tensile strength was the highest when PCL (Mw = 40,000 g/mol) was used [119].

The potential to use PLA/PCL blends in Fused Filament Fabrication (FFF) was examined in another work [120]. Using a twin-screw extruder, binary blends of PLA/PCL were prepared at various ratios (20/80 wt.% to 80/20 wt.%). Results of the study showed that the blends were immiscible; however, they showed sign of adhesion between the phases. Tensile properties were compared to those of injection molded blends, and both tensile properties were similar. Blends’ ductility was strongly driven by the behavior of its majority phase. 3D-printed blends were reported to have low porosity [120].

By using ROP, a series of linear and star shaped PCL with different arm numbers were successfully synthesized with the initiators having various number of hydroxyl functional groups [121]. After that, a micro compounder at a constant blending ration was used to melt PCL with PLA. Constant 1,4-phenylene diisocyanate (PDI) (1% weight) was also added as a commercial compatibilizer. Results of the study showed that star shaped PCL enhanced PLA’s mechanical properties. An increase in the percentage elongation was reported with the addition of star polymers. The percentage elongation increased from 4% to 9%. The three-armed star shaped PCL led to a substantial drop in modulus because of its high molecular chain mobility in comparison to linear, four- and six-armed PCLs. Images of the Scanning electron microscopy (SEM) showed that the immiscibility of the two biodegradable polymers were improved and therefore mechanical improvements were obtained [121].

For the aim of developing a degradable polymer blend for drug delivery applications, Ebrahimifar and Taherimehr [122] tested PCL, PLA, Polyvinylcyclohexane carbonate (PVCHC), in addition to the mixed polymeric matrix of PCL/PVCHC and PLA/PVCHC were tested as carriers for hydrophilic drugs acetaminophen and clindamycin. The highest release efficiency for PCL/PVCHC acetaminophen, PCL-acetaminophen, PLA acetaminophen, PLA/PVCHC clindamycin, PLA clindamycin and PCL clindamycin was found to be 29%, 38%, 39%, 40%, 95% and 96%, respectively [122].

Yang and co-authors [123] reported PLA/PCL blends at various concentrations. This was done using a novel extrusion device, eccentric rotor extruder. The addition of 20 wt.% PCL led to a substantial improvement in the percentage elongation at break to around 476.7%, which is more than 57 times that of the neat PLA. This was accompanied with a drop in tensile strength (20% drop). Due to the enhanced crystallinity of PLA as well as the compatibility of PLA/PCL blends, thermal stability was also improved [123].

Table 8 shows the effect of different PLA/PCL blends at various concentrations on the mechanical properties.

#### 2.3.3. Blends of PLA with Other Biodegradable/Renewable Resource-Based Polymers

Different studies have investigated blending PLA with various biodegradable/renewable resource-based polymers such as PPD [124], poly(propylene carbonate) (PPC) [125], poly(tetramethylene adipate-*co*-terephthalate) (PTAT) [126], poly(butylene adipate-*co*-terephthalate) (PBAT) [127], poly(ethylene/butylene succinate) (Bionolle) [128], poly(butylene succinate) (PBS) [129,130], poly(butylene succinate co-L-lactate) (PBSL) [129] and poly(butylene succinate-*co*-butylene adipate) (PBSA) [131].

In a study done by Pezzin et al. [124], PPD, a biodegradable polyester, was blended with PLA. The study showed that the PLLA/PPD blends exhibited higher modulus of elasticity and elongation at break. When only 20 wt.% of PPD was added to the PLLA phase (20/80 wt.% PLLA/PPD), the modulus of elasticity and elongation at break were roughly 1600 Mpa and 55% respectively, whereas these values were around 1400 Mpa and 15% for neat PLLA. On the other hand, the tensile strength of the blend was lower than that of the neat PLLA. Mechanical testing of these blends showed that they were tough and more flexible. Furthermore, the blends showed neck formation during elongation. This enhancement in the mechanical properties was attributed to the plasticizing effect of PPD. However, the mechanical properties of the other blends at compositions of 50/50 wt.% and 20/80 wt.%/(PLLA/PPD) were not improved, as compared to neat PLLA. Although the modulus of elasticity at both of these compositions were higher than that of neat PPD, the other values of stress at break, elongation at break, tensile strength and toughness were lower [124].

Blends of PLA and PPC (an amorphous degradable polymer) were prepared at various compositions by Ma et al. [125]. It was observed that for all types of blends, increasing PPC content resulted in a decrease in both Young’s modulus and tensile strength. Nonetheless, compared to neat PLA, increasing amounts of PPC resulted in an improvement in the tensile toughness. This increase in toughness was clear in concentrations higher than 40 wt.% PPC. This is due to the reason that when PLA was blended with PPC at a concentration less than 30 wt.%, PLA was the continuous matrix phase; however, for PPC concentrations higher than 40 wt.%, PPC was the continuous phase. Hence, the continuous PPC phase advocates the matrix yielding, therefore more energy was required to break the polymers [125].

PTAT is another biodegradable polyester that was blended with PLLA. Liu et al. [126] prepared PLLA/PTAT blends at different compositions by solution casting from chloroform. The tensile strength and percentage elongation for pure PLLA were 28 Mpa and 19%, respectively. Blend of PLLA/PTAT at a concentration of 75/25 wt.% exhibited a percentage elongation of 97% and a tensile strength of 25 Mpa, whereas the same were reduced to 34% and 7 Mpa, respectively for PLLA/PTAT blend with a concentration of 50/50 wt.%. The reason behind this could be related to the blend’s higher amount of phase separation as well as its low miscibility. For the 25/75 wt.% PLLA/PTAT blend, the elongation at break reported was around 285%, which is almost 15 times higher than that of neat PLLA. At the same time, the tensile strength was around 11 Mpa, that is slightly better than what was reported for PLLA/PTAT blend with concentration of 50/50 wt.%. These results suggest that PTAT was able to provide more ductility to the blend [126].

Melt blending of PLA with PBAT was studied by Jiang et al. [127]. PBAT is a biodegradable, flexible, aliphatic-aromatic polyester, with a percentage elongation of 700%. When PBAT was added to PLA at a concentration of 5–20 wt.%, the Young’s modulus and tensile strength of the blends decreased. For example, at 20% PBAT content, Young’s modulus decreased from 3.4 Gpa for neat PLA to 2.6 Gpa. Similarly, there was a reduction in the tensile strength from 63 Mpa for the pure PLA to 47 Mpa. These results are anticipated due to the fact that PBAT has a lower tensile strength and Young’s modulus when compared to PLA. As the content of PBAT increased from 5 to 20 wt.%, an enhancement in the Izod impact strength was observed. Maximum toughening was reported for 20 wt.% PBAT. This was also the case for the elongation at break, as the higher content of PBAT was used, higher elongation at break values were observed. This effect was noticeable even at very low PBAT content. For instance, with the incorporation of only 5 wt.% PBAT, the percentage elongation observed was more than 200%. As more content of PBAT was incorporated, the failure mode switched from brittle fracture for the neat PLA to a ductile fracture of the blend. This conclusion was supported by the SEM micrographs of the fractured surfaces. The SEM showed that as the content of PBAT increased, more and longer fibrils from the surfaces were spotted [127].

Using a single-screw extruder, different contents of Bionolle were blended with PLA [128]. Bionolle is an aliphatic biodegradable thermoplastic polyester. The percentage elongation for neat PLA was reported to be 2%, while the maximum percentage elongation for the blend was reported to be 8.2% with 40 wt.% Bionolle. On the other hand, as the amount of Bionolle increased, the Young’s modulus and tensile strength decreased. That was anticipated as Bionolle’s Young’s modulus and tensile strength are lower than those of PLA [128].

Shibata et al. [129] investigated the effects of blending PLLA with PBS and PBSL. Melt mixing followed by injection molding were used to blend PLLA with PBS or PBSL. PBSL can be referred to as a relatively new type of PBS. PBSL is a biodegradable polyester. Both the Young’s modulus as well as the tensile strength decreased as more concentrations of PBSL or PBS was added with the exception of blend of PLLA with a concentration of 1 wt.% and 5 wt.% of PBS. These blends have exhibited an increase in the Young’s modulus and tensile strength in comparison to neat PLLA. Field emission scanning electron microscopy micrographs were used to understand these results. The study attributed the results to the production of finely dispersed blends. Compared to pure PLLA, PBSL and PBS, all the blends exhibited significantly higher percentage elongation over the whole composition range. Overall, lower tensile strength and Young’s modulus but higher percentage elongation were observed for PLLA/PBSL in comparison to the PLLA/PBS blends with similar concentration [129].

In another work and for the purpose of enhancing PLLA’s mechanical properties, PLLA was blended with PBS [130]. The percentage elongation increased from 6.90% to 320.60% after the incorporation of 25 wt.% PBS.

A Blend of PLLA/PBSA was produced by Chen and Yoon [131]. Results suggested that the brittleness of PLLA was greatly improved at a composition of PLLA/PBSA 75/25 wt.% [131].

Table 9 shows the impact of different PLA blends with various degradable or partial degradable polymers at various concentrations on the mechanical properties.

#### 2.3.4. Features of Various PLA Blends

Blending PLA with PHB was found to improve the impact properties, percentage elongation at break [108,133,134], biodegradation rate [105,134] and barrier properties [107,135]. Enhanced ductility was also reported in case of blending with PBS [136,137] and PCL [138]. Improved barrier properties were also observed in case of blending with PHBV [139], PBS [140] and PBAT [141]. Table 10 shows the advantages of selected studies on PLA blends along with their applications.

### 2.4. Composites’/Nanocomposites’ Effect

Different studies have suggested the addition of different types of reinforcing fillers such as carbon nanotubes, talc and montmorillonite (MMT) into PLA to enhance its mechanical properties [145,146].

Adding MMT was reported to enhance the modulus of elasticity and flexural modulus of PLA based nanocomposites. Moreover, the molecular mobility of PLA chains can be restricted by the intercalated MMT particles [145,146,147].

In another investigation [147], the modulus of elasticity increased by 43% after the incorporation of 7.5 wt.% of MMT particles into PLA nanocomposites. The reason behind such enhancement in the stiffness (modulus of elasticity and flexural modulus) of PLA nanocomposites is the effective intercalation of MMT stacked layers in the PLA matrix which resulted in a bigger interfacial area that interacted with the matrix of PLA. Therefore, PLA rigidity resulted from the enhancement in interaction effect between the PLA matrix and MMT particles. In areas of higher interacted interfacial, the applied stress can be effectively transferred from the PLA matrix to the MMT particles. This can subsequently enhance the stiffness of PLA nanocomposites [147].

Balakrishnan et al. [145] investigated the effect of the number of MMT particles on the mechanical properties of PLA nanocomposite. Results showed that the addition of MMT particles into PLA has substantially enhanced the flexural modulus and modulus of elasticity by 18% and 10%, respectively. Results also showed that there was a gradual reduction in the flexural strength and tensile strength of the PLA composites by 25% and 10%, respectively. This was attributed to the increased number of MMT particles in the PLA matrix. This suggests that with the addition of more MMT particles, the particles reduced the interfacial adhesion effect between the MMT particles and PLA matrix because they agglomerated together. The agglomerated MMT particles acted as a stress concentration point in the matrix. Therefore, when they were exposed to an applied stress, they failed to evenly transfer that stress throughout the PLA matrix. Furthermore, the orientation and dispersion of MMT particles in the PLA matrix influence both the flexural strength and tensile strength due to the various orientations of applied stress between flexural bending and tensile straining. The same study showed that the impact strength of the PLA nanocomposite was lowered by 13% when the amount of MMT increased to 4 phr. However, there was a substantial enhancement in the impact strength of PLA/LLDPE nanocomposites by 53% and 21%, respectively, when 2 and 4 phr MMT particles were added. Results also showed that impact strength was induced when 10 wt.% of LLDPE was added to the MMT/PLA nanocomposites as compared to PLA nanocomposites. Therefore, it was found that a better orientation and dispersion of MMT particles in the PLA matrix was accomplished by the presence of LLDPE. This made the polymer matrix capable of absorbing more energy when subjected to rapid loading [145].

The effect of talc on the mechanical properties of PLA was investigated by Harris and Lee [148]. Both PLA’s flexural modulus and strength were substantially enhanced by 25% as a result of the addition of 2 wt.% talc. This was explained by the fact that talc particles acted as a nucleating agent which induced PLA’s crystallinity and thus enhanced the toughness of PLA. As a result of talc particles’ structure and orientation inside the PLA matrix, the applied stress can be effectively transferred to the PLA matrix from the talc particles. Therefore, the presence of talc particles can offer a reinforcement effect on the toughness and rigidity of the PLA matrix [148].

In another study [149], there was a substantial improvement in both the flexural modulus and strength of neat PLA with the addition of talc. An interesting observation was that when the content of talc increased from 0 to 2.0 wt.%, the flexural modulus and flexural strength of PLA increased rapidly. Such improvement is attributed to the substitution of the PLA matrix with highly rigid talc filler. Therefore, when subjected to external loading, talc filler could efficiently limit the mobility and extendibility of the PLA matrix. SEM analysis of the nanocomposite showed that a good interfacial adhesion effect between the PLA matrix the talc filler existed. Due to this reinforcing and toughening effect, the applied load was transferred evenly throughout the whole polymer matrix. Increasing the talc content to more than 2% wt.% resulted in a slight increase in the flexural modulus and flexural strength. The reason behind this decrease in effectiveness is due to the presence of thicker talc particles which in turn resulted from the insufficient delamination of talc particles. At higher talc filler content, the brittle behavior of the PLA matrix was dominant because the applied load was unable to be efficiently transferred from the polymer matrix to the talc filers. The reason behind that was the weak interfacial adhesion effect between the PLA matrix and the thicker talc particles. Results from the study have also suggested that at higher content of talc particles (more than 2 wt.%) there was a reduction in the orientation degree of talc particles. It was also found that the orientation direction of talc layers was not parallel to the injection direction. Thus, this has led to a debonding effect of talc particles and the PLA interface. The result was that many microcracks were presented along the direction of fracture [149].

An investigation of the effect of increasing talc and kaolin content on the properties of PLA composites was done by Ouchiar et al. [150]. The reported neat PLA’s modulus of elasticity was 2.4 GPa. However, with the addition of 5 wt.% talc content, the modulus of elasticity improved slightly to around 2.6 GPa. The addition of 5 wt.% of kaolin had a similar effect. When the talc and kaolin content increased from 5 to 30 wt.%, the results showed a gradual improvement in the modulus of elasticity of PLA composites. Nonetheless, PLA/talc composite reported a higher modulus of elasticity than kaolin/PLA composites. This is because when compared to kaolin-added PLA composites, an earlier crystallization was demonstrated by PLA/talc composites. This highlights the nucleation effect of talc and its feasibility in inducing the rigidity of PLA composites [150].

In an investigation done by Zhou et al. [151], the effect of using various contents of carbon nanotubes (CNTs) with carboxyl groups (CNTs-COOH) on the mechanical properties of PLA nanocomposites was studied. Results suggest that an increase in Izod impact strength, tensile strength and percentage elongation of PLA nanocomposites could be observed up to CNTs-COOH content of 0.5 wt.%. This demonstrates that enhancements in the impact strength and tensile strength of PLA is feasible with the addition of only a small amount of CNTs. This can be explained by the high stiffness of CNTs with high surface area and aspect ratio which could further enhance the PLA matrix’s toughness by efficiently interlocking in the PLA matrix. Due to this interlocking effect, the applied stress can be effectively transferred from the carbon nanotubes particles to the PLA matrix causing strengthening of the PLA nanocomposites. Another factor that aids in strengthening the PLA matrix is the strong chemical bonds between the PLA matrix and CNT-COOH particles which restricted PLA macromolecular chains’ mobility. Nonetheless, a further increase in the CNTs-COOH content above 0.5 wt.% reduced PLA nanocomposites’ Izod impact strength and tensile strength [151].

Similar results were also reported for another study [152]. The study showed that when more than 3 wt.% content of CNTs was added, a gradual decrease in the tensile strength of PLA nanocomposites was observed. This reduction in tensile strength can be explained by the increased content of carbon nanotubes in the PLA matrix which agglomerated together into larger carbon nanotube aggregates [151,152]. The presence of such CNTs aggregated in the PLA matrix and worked as point of stress concentration that weakened the applied load transfer throughout the PLA matrix. This has resulted in a reduction in the interfacial adhesion between the PLA matrix and CNTs [152].

Silva and co-authors [153] reported PLA/PHBV blends reinforced with carbon nanotubes. The incorporation of CNTs contributed to the electromagnetic and electrical properties of polymeric nanocomposites. The production of PLA/PHBV blend (80/20 wt.%) and PLA/PHBV blend based nanocomposites with 0.5 and 1.0 wt% of CNTs was reported. The incorporation of CNTs lowered the Izod impact strength, yet flexural properties remained not affected. The incorporation of 1.0 wt% CNTs yielded better electrical properties. Moreover, the nanocomposites demonstrated excellent result as electromagnetic interference shielding material [153].

Both of kenaf fibers and Multi-Walled Carbon Nanotubes (MWCNTs) were used by Chen et al. [154] to reinforce the PLA matrix. Increasing the content of kenaf fibers with the epoxy groups (KF-OX) up to 30 wt.% resulted in a gradual improvement in the MWCNT/PLA nanocomposites’ tensile strength prior to and after annealing. The chemical reaction between the PLA matrix and the KF-OX fibers was the main cause behind such improvement in tensile strength. When subjected to annealing, the tensile strength of the PLA nanocomposite became 84% higher than pristine PLA. This can be attributed to the good compatibility of KF-OX and the PLA matrix as well as to the creation of crystalline structure at the interfaces between the KF-OX fibers and PLA matrix which substantially enhanced the tensile strength of PLA nanocomposites. As a result of the outstanding interfacial adhesion effect between the PLA matrix and KF-OX fibers, the presence of transcrystallinity in the PLA matrix was able to deliver a resistance effect against applied external loading [155]. An improvement in the tensile strength was therefore observed due to the superb interfacial adhesion effect between the PLA matrix and KF-OX fibers which allowed the applied straining stress to be transmitted more efficiently to the PLA matrix from the KF-OX fibers. An improvement of KF-OX/MWCNT/PLA nanocomposites’ tensile strength was also noticed due to the recrystallization of PLA nanocomposites by the annealing process. Nonetheless, when the KF-OX content increased to above 40 wt.%, there was a drastic decrease in the PLA nanocomposites’ tensile strength. This can be explained by the possible obstruction of the recrystallization of PLA chains and weakening of the PLA nanocomposites’ stiffness caused by the extremely entangled KF-OX fibers in the PLA matrix [154].

**Figure 6:**
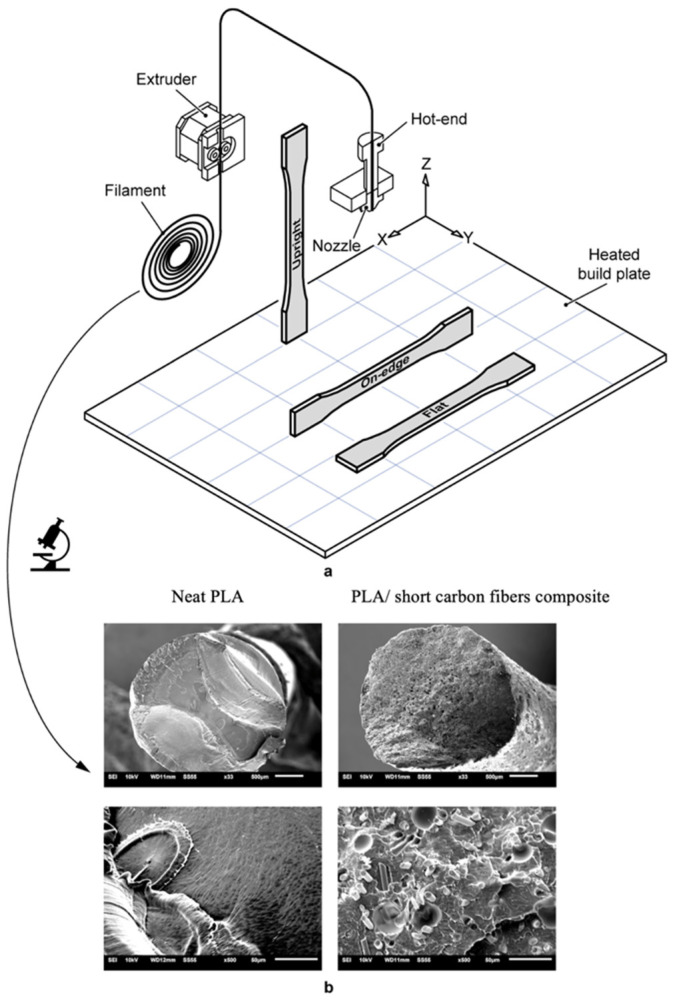
(**a**) A schematic showing the different printing orientations and (**b**) cross sectional SEM images of printing filaments of neat PLA and PLA/short carbon fibers composite [156].

The effect of carbon fiber on the mechanical properties of PLA based composites was also investigated, In one study [156], short carbon fibers were mixed with PLA using FFF technology. The mechanical properties of neat PLA as well as 3D-printed PLA/carbon fibers composites were studied at different printing orientations, namely “upright, on-edge and flat”, as shown in Figure 6. Results showed that the incorporation of carbon fibers enhanced the mechanical properties of the produced composites in comparison to the pure PLA. In comparison to the neat PLA, flat PLA/carbon fiber composite samples reported an average increase of 179.9%, 47.1%, 230.95% and 89.75% for tensile stiffness, tensile strength, flexural stiffness and flexural strength, respectively. Overall, the dimensional accuracy was not affected by the addition of short carbon fibers as reinforcements. Furthermore, enhanced surface roughness was reported in case of flat and on-edge PLA/carbon fiber composite samples. Results suggested that the prepared composite is a promising candidate for applications demanding dimensional stability and higher stiffness [156].

The effect of Cloisite 30B nanoclay, kenaf fiber and hexagonal boron nitrile (h-BN) fillers on PLA composites’ mechanical properties was also studied [157]. The study showed that there was a slight increase in the modulus of elasticity of the PLA composite before and after annealing treatment when 5 pph kenaf fiber, Cloisite 30B nanoclay and h-BN were added. When compared to both, Cloisite 30B nanoclay or h-BN, the addition of kenaf fiber resulted in a smaller increase in the Young’s modulus. This was attributed to the suitable compatibility between h-BN fillers and Cloisite 30B nanoclay with the PLA matrix. On the other hand, there was a weaker interfacial adhesion effect between the PLA matrix and kenaf fiber. The reason behind that was the absence of polar interaction between the PLA chains and kenaf fiber which ultimately resulted in less rigidity of the PLA composites [157].

A study on the mechanical properties of PLA/PCL and an organoclay nanocomposite was conducted by Hasook et al. [119]. The incorporation of organoclay resulted in an increase in the modulus of elasticity; however, the elongation at break and the strength decreased. With the addition of PCL to the PLA matrix, the modulus of elasticity decreased, whereas there was an increase in the percentage elongation and tensile strength of PLA/organoclay nanocomposites. Out of all the PLA/clay nanocomposite blends, the tensile strength was the highest when PCL (Mw = 40,000 g/mol) was used [119].

In another investigation [130], PLLA was blended with PBS and organoclay. PLLA/PBS at concentration of 75/25 wt.% with treated organoclay, TFC, as well as untreated organoclay, Cloisite 25A, were examined. When various amount of Cloisite 25A and TFC were added to the PLLA/PBS composite, the Young’s modulus was higher in comparison to that of PLLA/PBS blend. This shows that both of the Cloisite 25A and TFC exhibited a reinforcing effect due to their platelet structure and high aspect ratio. As the content of TFC clay increased, the Young’s modulus of the PLLA/PBS/TFC showed a pronounced effect in comparison to that of PLLA/PBS/Cloisite 25A. When Cloisite 25A was added, the PLLA/PBS composite’s percentage elongation decreased drastically. On the other hand, the elongation at break of the PLLA/PBS composite increased with the TFC content. Results showed that the blends with Cloisite 25A exhibited brittle fracture without necking, while composite blends with TFC, demonstrated higher necking. This suggests an increase in the interfacial interaction due to chemical bonds between the epoxy functional groups of the treated organoclay and PLLA/PBS blend which acted as a compatibilizer. Yield strength for the PLLA/PBS was around 44.70 MPa while the yield strength was maximum at a concentration of 10 wt.% of Cloisite 25A and TFC [130].

An investigation was carried out by Chen and Yoon [131] to compare the impact of incorporating treated and untreated organoclay, Cloisite 25A, on the PLLA/PBSA composite’s mechanical properties. The composition of PLLA/PBSA was set at 75/25 wt.%. This is because at this blend composition, the brittleness of PLLA was significantly improved. The treated organoclay was produced by reacting (glycidoxypropyl)trimethoxy silane (GPS) with Cloisite 25A to yield functionalized organoclay (TFC). Melt compounding of PLLA and PBSA with the organoclays at 180 °C resulted in the PLLA/PBSA/clay composites. Throughout the entire range of clay compositions, Young’s modulus of the PLLA/PBSA/Cloisite 25A/TFC composites was higher than that of the binary blend of PLLA/PBSA. That was anticipated, as the clay was used to reinforce the composite. On the other hand, the composite’s percentage elongation, both with organoclay Cloisite 25A and TFC, was significantly less than that of the of PLLA/PBSA blend. An interesting observation is that the composite with treated clay, TFC, exhibited higher modulus of elasticity and percentage elongation than that of untreated clay, C25. The reduction in agglomeration observed in PLLA/PBSA/TFC composite explains the higher elongation at break and Young’s modulus of PLLA/PBSA with TFC compared to those of PLLA/PBSA with C25 composite. As a result, this has contributed to more exfoliation and enhanced interaction between the functional groups of PLLA/PBSA and the epoxy group of TFC [131].

**Figure 7:**
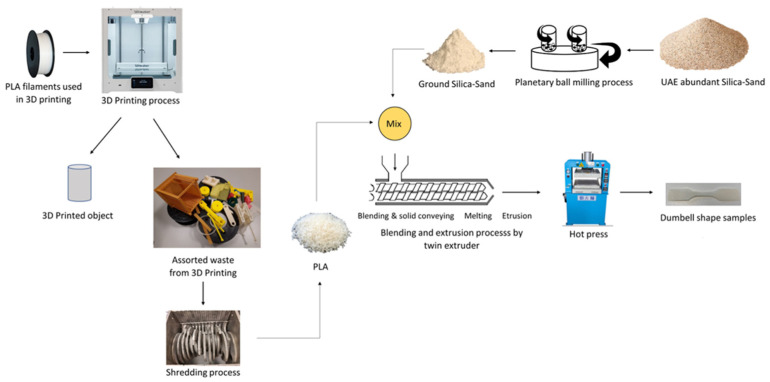
Preparation and fabrication of 3D-printed PLA wastes/SiO_2_ composites as reported by Ahmed et al. [158].

The effect of silica (SiO_2_) on the mechanical properties of PLA nanocomposites was investigated by Ahmed et al. [158]. They have used twin extruders to prepare composites of 3D-printed PLA wastes/SiO_2_ at various concentrations (95/5, 90/10 and 85/15 wt.%). This was followed by an analysis of the mechanical properties. Figure 7 shows a complete overview of the composites’ preparation and fabrication. Results showed that increasing the SiO_2_ composition up to 10 wt.% resulted in increasing the tensile strength, yield stress, Young’s modulus, ductility and toughness. A further increase of the SiO_2_ composition resulted in a drop in these properties. The produced composites can promote the effective recycling of PLA wastes from 3D printing applications [158].

The effect of magnesium oxide particles (nano-Mg) on the PLA nanocomposites’ mechanical properties was examined [159]. The authors reported a gradual increase in the Young’s modulus and tensile strength of the PLA nanocomposites as a result of increasing nano-MgO’s content up to 2 wt.%. Such increase was attributed to the nano-MgO particles’ smaller size which offered a higher interfacial area of magnesium oxide nanoparticles by inducing the volume ratio of these particles in the PLA matrix. This high surface interaction between the PLA matrix and the magnesium oxide filler, promoted the transfer of applied stress to nano magnesium oxide filler from the PLA matrix and eventually led to enhancement in the PLA’s mechanical properties. On the other hand, upon adding more than to 2 wt.% content of nano-MgO (up to 4 wt.% was used), the PLA nanocomposites’ Young’s modulus and tensile strength decreased gradually. This can be explained by the fact that higher content of nano-MgO tend to self-agglomerated into larger agglomerated particles which in turn has weakened the interfacial adhesion effect between the PLA matrix and the agglomerated nano magnesium oxide filler. As a result, the agglomerated nano magnesium oxide fillers were phase-separated from the PLA matrix and worked as a point of stress concentration in the PLA matrix. Thus, the effect of reinforcement of nano magnesium oxide fillers inside the PLA matrix was reduced [159].

Different studies in literature have analyzed the effect of natural fibers such as flax and kenaf fibers on PLA’s mechanical properties [160,161,162]. Their low cost, high specific strength, good toughness, biodegradability, renewability and low density have made natural fibers appealing substitutes to conventional reinforcing fillers in PLA composites [161]. Nonetheless, various studies have reported that PLA’s mechanical properties were substantially weakened as a result of the low compatibility of hydrophobic PLA with hydrophilic natural fibers [162,163]. This has hindered the use of PLA composites and nanocomposites in many applications.

Foruzanmehr et al. [161] found that the elongation at break and tensile strength were substantially improved after the addition oxidized flax into the PLA matrix. The reason behind that was the enhanced interfacial adhesion between the PLA matrix and fibers which has efficiently transferred the stress between the fibers and the PLA matrix [161].

In another investigation [164], film stacking and hot press compression molding were used to fabricate flax fiber braided yarn plain woven fabric reinforced PLA bio-composites. The impact of fiber weight fraction on the fracture and tensile properties was studied. Results showed that tensile strength and modulus were increased by around 60% and 62%, respectively for 35 wt.% braided fabric in comparison to neat PLA. This is attributed to the high value of plane-strain fracture toughness of braided fabric in comparison to other natural fibers. The interweaving yarns of the braided fabric exhibited high resistance, hence, more energy was required to initiate a crack propagation in comparison to other typical types of reinforcements [164].

The impact of coupling agent 3-glycidoxypropyl trimethoxy silane on the PLA/kenaf fiber composites’ mechanical properties was studied by Lee et al. [162]. Results showed a significant enhancement in the interaction between the PLA matrix and kenaf fibers as a result of treating kenaf fibers with 3-glycidoxypropyl trimethoxy silane [162].

The incorporation of wood flour with a surface treatment with different coupling agents, *γ*-glycidoxypropyltrimethoxy silane (epoxy silane), vinyltrimethoxysilane (vinyl silane), *γ*-methacryloxypropyltrimethoxysilane (allyl ester silane) and *γ*-aminopropyl triethoxysilane (amino silane) into the PLA matrix was also investigated [165]. There was a significant improvement in the elongation at break, tensile strength and impact strength of PLA/wood flour composites due to the incorporation of allyl ester silane, epoxy silane and amino silane. This was attributed to the enhanced interfacial interaction between the wood fibers and the PLA matrix as a result of the addition of silane coupling [165].

**Figure 8:**
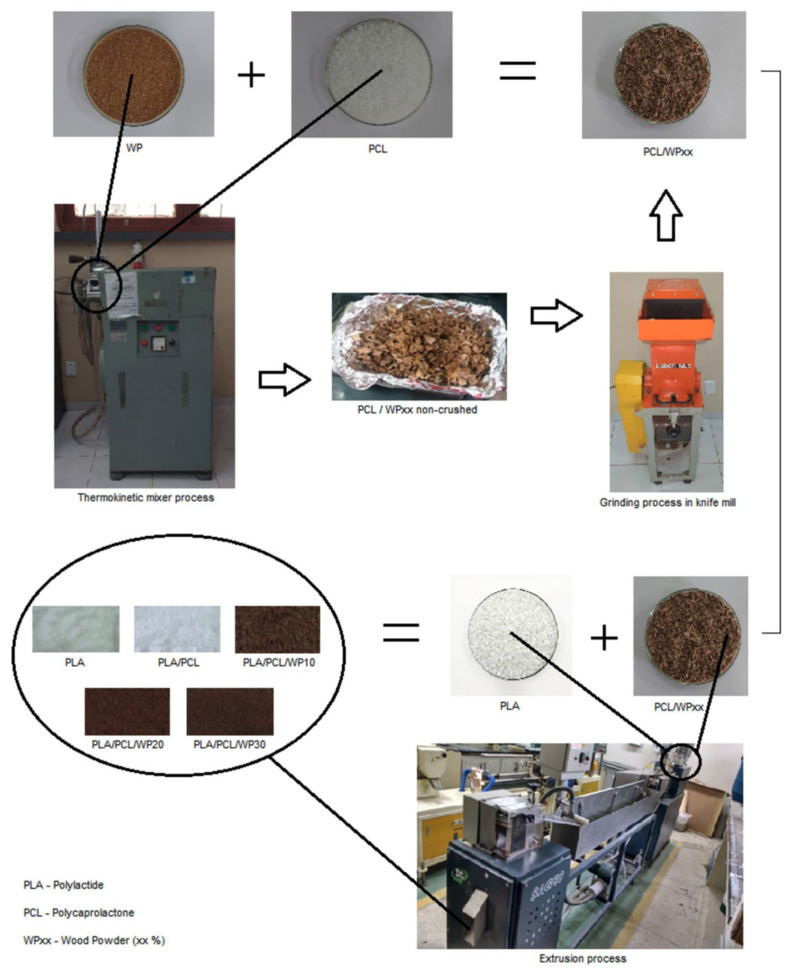
Extrusion process of PLA/PCL/wood powder composites. Reprinted with permission from Springer Nature, 2021 [166].

**Figure 9:**
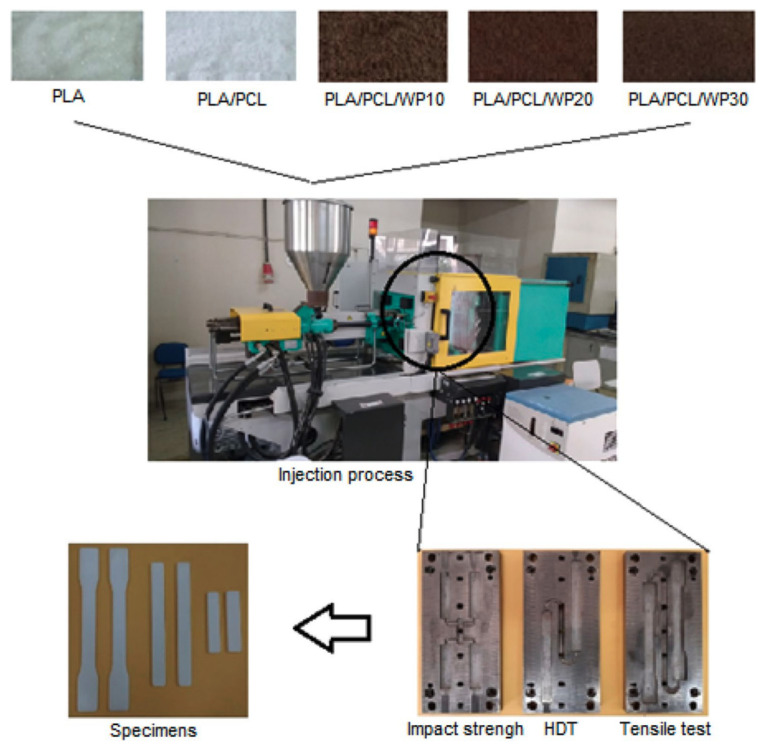
Injection molding process of PLA/PCL/wood powder composites. Reprinted with permission from Springer Nature, 2021 [166].

PLA/PCL composites using wood powder were prepared by Silva et al. [166] to examine their feasibility to produce disposable cups. Initially, the composites were prepared in a co-rotational twin screw extruder as shown in Figure 8. After that, extruded granules were molded via injection as illustrated in Figure 9. The impact strengths of the bio blend and composites were higher than that of neat PLA. Percentage elongation at break, shore D hardness as well as heat deflection temperature were roughly the same as neat PLA. On the other hand, losses were reported in tensile strength and Young’s modulus. The study concluded that such results are significant in promoting sustainability and recyclability [166].

In another investigation [167], PLA/lignin composite filaments were produced by mixing PLA with organosolv lignin at various ratios. Lignin was replaced with PLA up to 20 wt.%. For the aim of enhancing the mechanical properties of the campsites, two plasticizers, namely, PEG 2000 and Struktol (TR451) were added in different concentrations. Results showed that at 2 wt.% PEG, the tensile strength and percentage elongation at break were improved by 19% and 35%, respectively. On the other hand, TR451 was capable of improving the percentage elongation at break by 24% [167].

Paul et al. [168] reported the development of PLA/microcrystalline cellulose (MCC) bio composites via melt extrusion and compression molding. Triethylcitrate (TEC) was used as a plasticizer as well as to enhance the dispersion of the microcrystalline cellulose in the PLA matrix. Results showed improvements in crystallinity and ductility. Results of the mechanical and migration properties suggested that PLA/MCC bio composites with 10 wt.% TEC is the most suitable combination for ecofriendly food packaging applications [168].

Rasheed and co-authors [169] have studied the impact of CNCs (natural fiber) from bamboo fiber on the properties of PLA/PBS nanocomposites prepared by melt mixing followed by hot pressing. To improve PLA’s properties, they have added 20 wt.% PBS as well as cellulose nanocrystals at different concentrations (0.5, 0.75, 1, 1.5 wt.%). Results showed that the prepared biodegradable PLA/PBS blend had a homogeneous morphology. The nanocomposite showed rod-like cellulose nanocrystals particles embedded in the polymer matrix. Tensile strength, Young’s modulus and thermal stability all improved up to 1 wt.% due to the uniform distribution of the cellulose nanocrystals in the nanocomposites; however, percentage elongation at break reduced. According to the study, the developed nanocomposites can be completely degradable in soil, making it a feasible green candidate to conventional packaging materials [169].

Biodegradable nanocomposites prepared from PLA, PHB and CNCs were reported by Frone et al. [170] They have prepared the nanocomposites using a single step reactive blending with DCP as a cross-linking agent. The prepared nanocomposites were then processed using extrusion, compression molding and 3D printing. This was followed by an examination of the thermal, mechanical and morphological properties of these nanocomposites. The addition of DCP resulted in enhanced interfacial adhesion, improved dispersion of the CNCs in the nanocomposites as well as increased crystallinity. DCP and CNCs exhibited nucleating activity and favored PLA’s crystallization, increasing its crystallinity from 16% in PLA/PHB to 38% in DCP crosslinked blend and to 43% in crosslinked PLA/PHB/NC nanocomposite. In comparison to compression molded films, nanocomposites filaments produced by 3D printing and extrusion demonstrated higher storage modulus and onset degradation temperature. The study concluded that PLA/PHB blends and nanocomposites with improved interfacial adhesion, enhanced mechanical properties and thermal stability can be produced following the right choice of processing approach and using DCP and CNCs for properties balance. If processed correctly, such nanocomposites have high potential in meeting the high standards of industrial engineering applications [170].

Alam et al. [171] reported the mechanical properties of 3D-printed novel nanocomposite scaffolds. The scaffolds consisted of a blend of PLA and PCL reinforced with halloysite nanotubes (HNTs). Melt blending was used to develop the nanoengineered filaments while FFF was used to fabricate the nanocomposite scaffolds. The study reported a uniform dispersion of the HNTs inside the blend’s matrix. According to the study, the loss in mechanical properties as a result of the incorporation of PCL to PLA was fully recovered by the incorporation of HNTs. Degradation rate, in terms of weight loss, was dropped from 4.6% for neat PLA to 1.3% for PLA/PCL blend. However, that was gradually increased to 4.4% after the addition of 7 wt.% HNTs. Results showed that the mechanical properties, biodegradation rate as well as biological characteristics of the 3D-printed micro architected PLA/PCL/HNT composite scaffolds can be tuned by a suitable combination of PCL and HNTs contents inside the PLA matrix [171].

**Figure 10:**
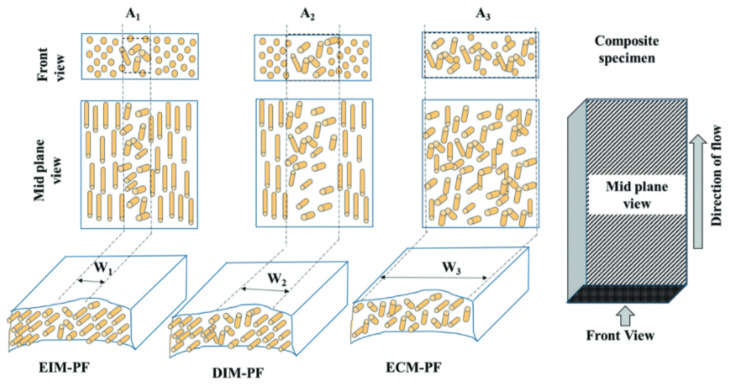
A schematic of the distribution of PFs in the various composites, note, “A” is core area. Reprinted with permission from Taylor & Francis, 2021 [172].

Recently, Komal et al. [172] were able to fabricate pineapple fibers (PFs)/PLA bio composites using direct injection molding (DIM) without compounding, with compounding using extrusion followed by injection molding (EIM) as well as with compounding using extrusion followed by compression molding (ECM). Figure 10 shows a schematic of the distribution of PFs in each of these composites. Results showed that the mechanical response, crystallinity and viscoelastic response of the EIM composites substantially dominated the composites fabricated by the other two approaches. The study has also reported a severe attrition of fibers during ECM. Nonetheless, T_g_, T_m_ and crystallization temperature were found to be independent of the fabrication approach [172].

In another investigation [173], solution blending was used to fabricate chitosan/PLA composites doped with graphene oxide (GO). GO was added into a PLA solution before blending it with chitosan. Thermal and mechanical properties in addition to the water barriers of various compositions of the chitosan/PLA-GO composites (90/10/2, 70/30/2 and 50/50/2 wt.%) were analyzed. Results suggested enhanced miscibility of chitosan and PLA, improved thermal stability as well as increased tensile strength and modulus due to the addition of GO. Moreover, chitosan/PLA-GO composites reported excellent water barrier properties. The highest decrement in water absorption was for chitosan/PLA-GO (70/30/2 wt.%) composite. The study concluded that the prepared composites with GO have high potential to be used in biomedical applications such as drug delivery. Furthermore, the developed composites can be also utilized in food packaging applications [173].

Table 11 shows the impact of various PLA composites and nanocomposites at various concentrations on the mechanical properties along with their applications.

## 3. PHAs’ Modifications

PHB shows similar Young’s modulus and tensile strength in comparison to PP. Nonetheless, it suffers from a drastically low percentage elongation (5–10%) [174,175]. HV’s molar ratio affects the mechanical properties of PHBV [176]. Mostly, enhancements in flexibility and toughness can be noticed as a result of increasing the HV fraction, this is accompanied with a gradual decrease in the tensile strength [177]. PHBV with an HV molar ratio of 30 to 60 mol%, exhibits a high degree of softness [175]. The Young modulus of PHAs ranges from the stiffer scl-PHA (3.5 × 10^3^ MPa) to the very ductile mcl-PHA (0.008 MPa) [178]. PHAs’ tensile strength ranges from 8.8 to 10^4^ MPa [178]. Table 12 shows the mechanical properties of PHB and PHBV as well as of some other commercial polymers. The use of PHB in many applications today is hindered due to its poor mechanical properties, mostly on account of its high fragility [179,180,181]. PHBV exhibits better mechanical properties such as flexibility, toughness, manufacturability and impact resistance than PHB [182]. Despite some of the improvements it offers over PHB, PHBV exhibits low impact resistance, high fragility and poor thermal stability compared to petroleum-based polymers [183]. Moreover, the high production cost of PHAs with respect to synthetic plastics has hindered their wide in many applications including packaging. Therefore, blending PHAs with other synthetic plastics and nanofillers has been found to tailor PHAs’ properties and overcome such problem by introducing new materials with excellent mechanical and thermal characteristics, better barrier properties and biodegradability [184]

### 3.1. Blending’s Effect

Due to their nontoxicity, biodegradability and hydrophobicity, PHAs have been widely used in many applications worldwide. Recently, and due to the raised awareness of the environmental concerns, the production of PHA has increased significantly. PHAs are proven to be a good competitor for food packaging applications. Nonetheless, the high production cost of PHAs is the main obstacle for expanding their productions to the commercial scale. Blending PHAs with other polymers has been reported to be a good option to increase their flexibility.

Moreover, PHAs based blends exhibit good degradation rate. In one study [186], the degradation of PHB, PCL and PCL/PHB 70/30 wt.% blend was investigated. Results showed that both neat PCL and PHB samples were degraded with strong erosion of the amorphous zones. After 20 days of incubation, the PCL/PHB 70/30 blend showed that spheres of PCL were bordering with spherulites of PHB demonstrating complete degradation. At various degradation times, the crystallinity content of homopolymers and blend were analyzed. Whereas there was no change in the PCL’s crystallinity, the crystallinities of PHB and the blend’s PHB-phase have increased [186]. Using solution blending, high molecular weight PHB/PCL and PHB/low-molecular-weight chemically modified PCLs (mPCL) were prepared [187]. The crystallization, morphology as well as the enzymatic degradation of the blends after exposing them to Aspergillus flavus were studied. Throughout the entire composition range, high-molecular-weight PHB/PCL blends were found to be immiscible. Results showed a drop in the PHB nucleation density and a fractionated PCL crystallization. PHB/mPCL blends were partially miscible; two phases were formed, but the PHB-rich phase demonstrated clear signs of miscibility. Biodegradation results showed that the blends were degraded more than the homopolymers. The study claimed that the dispersion of the components and their crystallinity can affect the improved degradation rate of the blends. The PHB/mPCL blends exhibited a drop in the degradation rate due to the increased miscibility between the components [187]. Due to their good mechanical and thermal properties, PP and PE have been commonly used in the packaging industries. Nonetheless, they are resistant to microbial degradation. To overcome this limitation, PP and PE have been blended with PHAs. For instance, in one study [188], the biodegradability of various films made out of PE, PHBV as well as PE/PHBV blends was evaluated using a respirometry test after 180 days. Results suggested that the degradation rate was proportional to the quantity of PHBV in contact with PE [188]. In another study [189], melt blending was used to investigate the application of the PHBV copolymer as a biodegradable additive in PP. The study reported a successful production of PHBV/PP blends. The degradation rate of the produced blend was studied in the field as well as in controlled laboratory conditions. Results of the SEM showed biofilm formation due to microbial activity on the surface of the treated films. The degradation of PHBV/PP blends was found to be due to an oxo-biodegradation process. Microorganisms’ attachment to PHBV/PP film turns it into material with a higher degree of crystallinity as a result of polymeric chain scission caused by the oxidation process [189].

Blending PHAs with other polymers such as PP [189,190], PE [188,191], poly(ethylene terephthalate-*co*-1,4-cyclohexanedimethanol terephthalate) (PETG) [192], poly(butylene succinate) (PBS) [144,193] and PLA [43,87,88,101,104,105,106,107,194] have been reported to enhance the mechanical properties of PHAs.

Binary blends of PHB and PCL were produced by Garcia et al. [195]. Thermal and mechanical properties of the blends were studied. Moreover, the miscibility and blends morphology were investigated in terms of the blend composition. Binary PHB–PCL blends were developed using melt compounding in a twin screw co-rotating extruder and injection molded. Results claimed that PCL acted as an impact modifier. Therefore, increasing PCL content led to an increase in the blend’s flexibility and ductility. Moreover, there was a significant increase in the percentage elongation at break and the energy absorption in impact conditions. Furthermore, when 25 wt.% of PCL was blended with PHB, the resulted blend showed the peak value for the flexural strength and flexural modulus. A drastic drop in these values was reported when further PCL was added. On the other hand, increasing the content of PCL led to a drop in both the tensile strength and Young’s modulus. The study has also reported a clear evidence of the immiscibility of the blend. The same was also reported in another study [196]. Furthermore, an increase in crystallinity of both PHB and PCL was reported for PHB/PCL blends containing 25 wt.% PCL. The study has also reported an increase in the degradation onset of about 30 °C [195].

For the purpose of widening the application of multi-scale instrumental analyses to include biodegradable polymers, plasticized PHA containing 65% PHA, 30% PBS and 5% crosslinking agent were investigated with respect to blending with PCL. The same was also studied after the incorporation of compatibilizers, such as crosslinkers and graft polymers [197]. Results showed that when PHA was blended with more than 30% PCL, there was an increase in the percentage elongation at break as well as the tensile strength in the quasi-static tensile test. On the other hand, impact tensile properties were less enhanced by the addition of PCL. This might be attributed to the molecular mobility suppression as a result of blending. Graft polymers led to a minor decrease in the percentage elongation at break in the quasi-static tensile test while a significant drop was observed in case of crosslinkers. However, with respect to the impact tensile test, both of the graft polymers and crosslinkers led to an increase in the percentage elongation at break and tensile strength [197].

Recently in another study [198], natural medium chain length PHAs (poly(3-hydroxyoctanoate-*co*-3-hydroxydecanoa (P(3HO-3HD))) was blended with PCL at two concentrations, namely, 75/25 wt.% and 95/5 wt.%. The blends were intended to combine the outstanding ability of PHAs to support the growth and proliferation of mammalian cells with PCL’s good processability. The blends were intended to be transformed into a new biomimetic Nerve Guidance Conduit (NGC). The fabricated blends demonstrated superb neuroregenerative properties and a good bio resorption rate. The blends are to be used in the manufacturing of hollow NGCs to support nerve regeneration in 10 mm sciatic nerve gap in rats. Compatibility of the blend with large-scale manufacturing of NGCs was illustrated via the production of porous tubular devices with two wall thickness values. Results showed that the devices exhibited a good porosity/permeability relationship, and therefore permitting excellent nerve regeneration ability whilst maintaining low biodegradation rate and enough stiffness to protect the nerve throughout the whole regenerative process. Results showed that when the content of PCL exceeded 30 wt.%, PCL started to dominate the blends’ mechanical properties yielding to substantially stiffer materials than neat P(3HO-3HD) [198].

PBS is a linear, aliphatic, crystalline polyester with excellent mechanical properties and biodegradability. Due to their weak interfacial adhesion, poor compatibility as well as their large particle size, Qiu et al. [199] reported difficulties in fabricating PHB/PBS blends. Yet, in another investigation, Qiu et al. [200] were able to use the solvent casting method to fabricate PHBV and PBS blends (80/20 wt.% and 20/80 wt.%). A drop in the PHBV’s crystallization rate was observed as the content of PBS increased. The immiscibility of PHBV with PBS was demonstrated through the lack of change in the glass transition temperature as well as the biphasic melt of the blend [200]. In order to overcome the problem of immiscibility, Ma et al. [193] fabricated PHB/PBS and PHBV/PBS blends using pure PHB, PHBV. Situ compatibilization method with DCP which is a free-radical grafting initiator was used. Results showed a significant enhancement in the elongation at break of the PHBV/PBS blends due to the better interfacial adhesion between the PHBV and PBS phases. Furthermore, the deformation, dilatation, as well as the fibrillation of the PBS particles in the polymeric matrix of PHBV led to an improvement in the tensile strength [193].

A ternary blend of entirely biodegradable polymers, namely PLA, PHBV and PBS was fabricated by Zhang et al. [144] via melt compounding. Various blends of PLA/PHBV/PB (60/30/10 wt.% and 60/10/30 wt.%) as well as PHBV/PLA/PBS (60/30/10 wt.% and 60/10/30 wt.%) were produced and examined. The blends’ mechanical properties, thermal properties, thermal resistance, morphology as well as miscibility were studied. Results of the Dynamic Mechanical Analysis (DMA) suggested PHBV and PLA exhibited some limited miscibility with each other, yet PBS was found to be immiscible with PHBV or PLA. A minor phase-separated structure was reported from SEM for all the blends composition except for that of the PHBV/PLA/PBS (60/30/10 wt.%) blend. The same blend was also found to demonstrate a typical core-shell morphology with outstanding stiffness–toughness balance. An enhancement in the PLA’s crystallization, flexibility and toughness was observed in the resulting ternary complex [144].

Using castor oil cake (CC) as a filler, Burlein and Rocha [191], were able to fabricate PHB/LDPE blends by melt mixing. There was a substantial improvement in the LDPE’s modulus of elasticity accompanied by a decrease in the impact resistance and other tensile properties with the incorporation of PHB or CC. This can be explained by the unsatisfactory dispersion of the CC in the LDPE as well as the weak interfacial adhesion between the components of the mixture [191].

Because of its outstanding water and moisture barrier properties, PETG has been widely used in the packaging applications. For the aim of enhancing PHBV’s processability, a twin-screw extruder was used to mix PHB and PETG [192]. When compared to neat PHB, the extruded and injection molded blends were found to exhibit a substantial enhancement in the flexural modulus. This can be attributed to the good dispersion of PETG in the PHB. Blends containing 20 wt.% and 30 wt.% of PETG were found to exhibit an impact resistance that is comparable to the value of that of PHB. Overall, the incorporation of PETG to PHB was proven efficient in enhancing the processability and modulus of elasticity without significant changes in the impact resistance. The biodegradability of PHB was also intact [192].

In another study [201], a newly developed poly(3-hydroxybutyrate-*co*-3-hydroxyvalerate-*co*-3-hydroxy-hexanoate) (P(3HB-*co*-3HV-*co*-3HHx)) fabricated by mixed microbial culture using biomass derived from fruit pulp was mixed with commercial PHBV at concentrations from 10 wt.%. to 50 wt.%. Neat PHAs in addition to the produced PHBV/P(3HB-*co*-3HV-*co*-3HHx) blends were subsequently thermo compressed to yield films that were characterized based of their optical characteristics, morphology, mechanical, barrier and thermal properties. This was followed by a detailed analysis to assess their potential in food packaging applications. Results showed good optical properties and interpolymer miscibility. There was no significant impact on the thermal stability of the blend. Moreover, permeability to limonene vapor, water and oxygen gas was reduced in the blends. Furthermore, the blend exhibited more flexibility than the neat rigid PHBV due to the plasticizing effects introduced by the terpolymer. The Young’s modulus and tensile strength of the terpolymer were lower than those of PHBV. This might be attributed to the interference with the crystallization process, the higher the 3HHx fraction in the P(3HB-*co*-3HV-*co*-3HHx), the higher the increase in flexibility. Results have also showed an increase in the percentage elongation at break with increasing the terpolymer content. Therefore, the mechanical response changed from a rigid but fragile to a more ductile behavior after blending PHBV with P(3HB-*co*-3HV-*co*-3HHx) [201].

### 3.2. Composites’/Nanocomposites’ Effect

Nanocomposites are hybrid material containing polymer matrix reinforced with particle, fiber and clay, with at least one component in nanometer scale. Nano clays or nanofillers are usually added to alter the mechanical, thermal and barrier properties of the resulting materials. Furthermore, they are also incorporated to modify the crystallization behavior, rate of degradation as well as the morphology. PHA-based nanocomposites have been fabricated using Cloisite 25A [202], carbon nanotubes (CNTs) [203,204], organ modified montmorillonite (OMMT) [205,206,207], multi Na-montmorillonite Cloisite Na (Na-MMT), a methyl tallow bis-hydroxyethyl quaternary ammonium-modified MMT Cloisite 30B [208,209,210], cellulose nanowhiskers (CNWs) [211,212,213], SiO_2_ nanofibers [214], carbon nanofibers (CFs) [215], halloysite nanotube (HNT) [209] and CNCs [103,105,106,107,216].

Carbon nanotubes can be defined as cylindrical nanostructures in which graphene layers are arranged as stacked cones, cups, or plates. They consist of concentric cylinders of graphite layers [215]. Due to their effectiveness in enhancing the hardness, electrical conductivity as well as the thermal stability of polymer-based composites, CNTs have been incorporated into various PHAs based nanocomposites.

Solution processing was used to develop PHBV/Multiwalled Carbon Nanotubes (MWNTs) nanocomposites by Lai et al. [204]. Results from the investigation suggested that there was an improvement in the nanocomposite’s thermal stability due to the homogeneous dispersal of MWNTs inside the PHBV matrix [204].

In another study [203], the crystallization behavior of PHBV after the addition of MWNTs was investigated by Shan et al. The incorporation of the multiwalled carbon nanotubes was found to substantially increase PHBV’s crystallinity and crystallite sizes [203].

In their examination, Liao and Wu [217] used melt blending to develop PHB/MWNTs nanocomposite. In order to enhance the compatibility as well as the dispersion of the MWCNTs within the PHB matrix, the authors used acrylic acid grafted poly(3-hydroxybutyrate) (PHB-*g*-AA) and multihydroxyl functionalized MWNTs (MWNTs-OH) as alternatives. The PHB-*g*-AA/MWNTs-OH blend exhibited a significant improvement in the mechanical and thermal properties of the PHB. It is believed that such an improvement is as a result of the formation of ester carbonyl groups through the reaction between carboxylic acid groups of PHB-*g*-AA and hydroxyl groups of MWNTs-OH. Due to the incorporation of 1 wt.% MWNTs-OH, there was an increase of 15.1 MPa and 75 °C in both, the tensile strength and the initial decomposition temperature, respectively. The study concluded that a 1 wt.% MWNTs-OH was the optimal amount. A further addition of MWNTs-OH led to separation of the organic and inorganic phases and a reduction in the compatibility of PHB-*g*-AA and MWNTs-OH [217].

Using solvent casting, Sanchez-Garcia et al. [215] were able to successfully develop PHBV/carbon nanofibers nanocomposites. The study showed that a substantial increase in the thermal, mechanical as well as the barrier properties was obtained due to the addition of the carbon nanofibers. Moreover, an increase in the conductivity of the resulted nanocomposite was also reported [215].

In another investigation [218], carbon nanofibers were chemically modified by *n*-octanol, silane coupling agent (KH-550) as well as nitric acid (HNO_3_) and then then added to poly-3-hydroxybutyrate-*co*-4-hydroxybutyrate (P3HB-*co*-4HB). The study reported a significant increase in the crystallinity and the glass transition temperature of the developed nanocomposites due to the addition of small diameters and uniform thickness carbon nanofibers treated with HNO_3_ [218].

In a similar work, Gumel et al. [219] reported a substantial increase in the lattice strain (17%), crystallite size (66%) and micromolecular elastic strain (46%) after the incorporation of carbon nanofibers (10% *w*/*w*) to mcl-PHAs [219].

In another investigation [220], melt blending was used to prepare nanocomposites containing a PHA biopolyester and graphene nanoplatelets (GNPs) or hybrid nanocomposites consisting of a PHA biopolyester, GNPs and carbon nanofibers. Results showed that the fabricated nanocomposites demonstrated good mechanical properties and improved thermal stability. The electrical conductivity has also increased significantly with the best performance obtained at 15 wt.% of the hybrid filler, which was around six times higher than that of the of the pure GNP nanocomposites at the same loading. Hybrid nanocomposites’ electromagnetic interference shielding performance was reported to be around 50% better than that of the pure GNP reinforced nanocomposites. Both types of nanocomposites exhibited a significant increase in the thermal conductivity, yet the hybrid nanocomposites reported better performance. Young’s modulus and tensile strength were also higher for the hybrid nanocomposites. As a result, the reported nanocomposites can be considered as promising candidates to substitute petroleum-based polymers in thermal and electrical applications [220].

The effect of molecular weight, H_x_ content of the PHBH_x_ as well as the type of SiO_2_ particles on the poly(3-hydroxybutyrate-*co*-3-hydroxyhexanoate) (PHBH_x_)/SiO_2_-based nanocomposites’ mechanical properties was investigated by Xie et al. [214]. Two different molecular weight (903,000 g/mol and 633,000 g/mol) and Hx content (6.9 and 7.2 mol%) were used in this study. Furthermore, two types (nominally spheres and fibers) of SiO_2_ were also examined. There was a 34% and 30% increase in both, the toughness and the modulus of elasticity of the developed nanocomposites due to the incorporation of 1 wt.% SiO_2_ fibers to the high molecular weight PHBH_x_ (7.2 mol% H_x_). On the other hand, the developed nanocomposites reported a slight improvement in thermal stability. In case of nominally spheres SiO_2_, the same increase of modulus of elasticity was reported for the high molecular weight PHBHx, nonetheless, the increase in toughness was limited to only 11%. When more SiO_2_ fibers (3 wt.%) were incorporated into the PHBH_x_ matrix, the elongation at break as well as the toughness decreased, yet the modulus of elasticity increased. The authors concluded that in order to enhance the stiffness and the toughness of the PHBH_x_ nanocomposites, a high molecular weight of the polymer matrix, a good dispersal of the SiO_2_ nanofillers and a weak interfacial adhesion are essential [214].

Starch has been also added to PHAs for the purpose of reducing their cost. Although other inexpensive fillers such as ground minerals can be used, yet the advantage of starch is that it is already in a fine powder form and it is completely biodegradable. Furthermore, starch can work as a reinforcing filler and therefore can improve the strength and modulus of the nanocomposite. Another advantage of starch is that it can impact the overall degradation rate of PHA/starch blends because starch biodegrades in a very short time [221,222,223,224,225].

Both the mechanical properties and the biodegradation rate of starch-PHBV composites were studied by Ramsay et al. [225]. As the starch content increased, percentage elongation at break and tensile strength exhibited a significant drop. This might be attributed to the weak adhesion between phases. SEM investigation showed a separation of starch granules from the PHA matrix. Yet and due to the rigidity of the starch granules, Young’s modulus of the PHA/starch blend demonstrated an increase with increasing the content of the starch [225]. Other studies in literature have also reported similar results [221,222,223,224].

For the purpose of improving the adhesion between starch and PHAs, two main methods were used [226]. Firstly, the incorporation of coupling agent. For example, in one study [222], a substantial increase in both of the strength and percentage elongation at break were reported for coated starch with polyethylene oxide (PEO)/PHBV composites. Yet, the values for these mechanical properties were less than that of neat PHBV. The study concluded that PEO can serve as a binding age because it has a favorable interaction with both starch and PHBV [222]. The second method consists of modifying the starch and/or PHA chemically. For instance, in one investigation [226], a free-radical former (2% bis[tert-butylperoxyisopropyl] benzene) was added to PHBV/starch 80/20 wt.% and 70/20 wt.%. The study reported that due to this addition there was an increase in the impact resistance from 1.8 kJ/m^2^ for neat PHBV to 2.10 kJ/m^2^ for PHBV/starch 70/20 wt.%. It is believed that some starch–PHBV graft copolymer was produced via free radical combination reactions and worked as an interfacial binding agent [226].

Another study [227] reported enhanced percentage elongation at break and tensile strength for blends of PHB/starch copolymerized with diisocyanate and propylene glycol. Nonetheless, the values were less than those of neat PHB [227].

Compared to untreated starch/PHBV composite, composites of starch-g-poly (glycidyl methacrylate) (>7% PGMA) and PHBV exhibited substantially higher tensile and flexural strengths [228]. However, there was no significant increase in both of the Young’s modulus and percentage elongation at break. All samples were immersed in water for 28 days. The gains in weight for PHBV–starch bars with 25% starch were about 4–5% compared with 0.9% for PHBV alone and 40–50% for starch. After soaking, percentage elongation at break increased, Young’s modulus decreased and the tensile strength remained unaffected [228].

Liao and Wu [229] claimed that the tensile strength for composites of starch (50%) with acrylic acid-grafted PHB exhibited 7 MPa increase when compared to the tensile strength value of the unmodified starch/PHB [229].

Blends of starch acetates with PHBV [230,231] were found to be brittle and incompatible. On the other hand, for starch valerate contents lower than 20%, blends of starch valerate and PHBV were believed to be compatible [232].

For the aim of reducing the cost and enhancing the properties of PHB, Godbole et al. [233] investigated PHB’s compatibility with starch. All the blends were reported as crystalline. For the blend of PHB/starch, 30/70 wt.%, a substantial improvement in the tensile strength was found in comparison to neat PHB. Due to the low cost of starch, the study claimed that blending PHB with a maximum content of 30 wt.% starch can significantly reduce the cost of PHB while maintaining its physical properties. The study concluded that the developed blend can be used in the food packaging applications such as a coating material on paper or cardboard [233].

In another study [234], casting was used to blend polyhydroxybutyrate-hydroxyvalerate (PHB-HV) with maize starch at various starch contents. Results showed that the tensile strength, modulus of elasticity and percentage elongation at break decreased with increasing the starch content. Results has also suggested that PHB-HV and starch are immiscible [234].

PHB was blended with two types of maize starch, Starch 1 (containing 70% amylose) and Starch 2 (containing 72% amylopectin) [235]. The blends, PHB/starch (70/30 wt.%) were produced via melt compounding. Results of the study showed that starch granules acted as a filler as well as a nucleating agent leading to a very substantial drop in the size of the PHB spherulites. Substantial enhancement in mechanical, rheological and thermal properties were reported. The study showed that the improvements were greater for PHB/starch1 than those of PHB/starch2. This might be due to the improved hydrogen bonding between PHB and Starch 1 with high-amylose content [235].

PHAs and thermoplastic starch were used to come up with novel flexible materials [236]. The starch was initially plasticized with high glycerol content followed by blending with PHBV and PBAT. The investigators claimed that the starch phase was miscible with PHBV and PBAT phases independently. Although the produced material had 70% biobased content, it exhibited excellent mechanical properties that are ideal for flexible packaging [236].

Using coextrusion, glycerol-plasticized starch films laminated with PHBV were developed by Martin et al. [237]. The results showed a gradual decrease of the peel strength as the content of glycerol in the plasticized starch increased. The study has also concluded that thermoplastic starch foams and films laminated with a thin layer of PHA seem to be appropriate for applications involving short-term contact with water. This is was due to the lack of major swelling of the thermoplastic starch films extrusion laminated with polyesters after soaking in water for a few days [237].

Creating a rough interface during the coextrusion process was found to be effective in improving the peel strengths of polyesters on thermoplastic starch. PHBV’s adhesion to thermoplastic starch using only water as a plasticizer was greater than when glycerol was incorporated [238].

The use of thermoplastic starch/PHA laminates and foams has been also reported in literature [239]. In such structures, PHA makes up a small component, between 5–20% or less, while the majority of the structure consists of thermoplastic starch. The PHA works as a water-resistant outer coating. Simultaneously, the PHA supports the foam expansion process. Studies on coating starch-based foams and films with PHBV have been also reported [239].

In one investigation [240], foams of extruded starch/PHBV with 5–20% PHBV were developed and their properties were reported. Results suggested that the addition of PHBV has significantly improved the expansion of the foam. The majority of PHBV existed as separate elongated inclusions with a length of approximately 1–5 mm within the starch matrix. On the other hand, PHBV was found to enrich the surfaces of the foam. This might be attributed to the lower surface energy of PHBV compared to starch. Therefore, the starch/PHBV foams exhibited significantly greater water resistance than starch foams and friability was reduced [240].

Many studies in literature have reported the biodegradation of starch–PHA blends in various environments [224,225,226,241,242,243,244,245,246,247,248,249,250]. A summary of a selected number of these studies is shown in Table 13. In these studies, the biodegradation of PHA was investigated in different environments such as compost, soil, activated sludge under anaerobic and aerobic conditions as well as marine environments. In all of these environments, blends of starch–PHA were found to be biodegradable over a period of weeks to months. Factors such as moisture content, crystallinity and molecular weight of polymers, temperature, presence of starch/PHA degraders, presence of plasticizer, sample thickness and microbial activity were found to affect the biodegradation rate. The biodegradation rates of PHA/starch blends were found to be higher in activated sludge and compost. This might be due to the high temperature as well as the availability of high numbers of PHA depolymerase-producing microorganisms.

In one study, when compared to PHA degraders, the starch-degrading microorganisms were about 10 times more abundant. Therefore, the starch portion of the PHBV/starch composite degraded far before the PHBV did [250].

In most of the investigations in literature, the rate of biodegradation of PHA-based/starch composites increased with increasing the content of starch. This might be attributed to the creation of more surface area for microbial attack after the removal of the more rapidly degraded starch. Exposing starch/PHBV and PHBV to aqueous environments has led to slow but significant rates of hydrolysis. For long-term applications such as consumer durables, this might be a key factor for PHA blends [244,247]. Despite the fact that biodegradation of starch–PHBV blends is a good option, recycling of the blends back into monomeric hydroxyacids is considered today as a more attractive choice [251,252]. This can be easily done through enzymatic depolymerization. New PHAs can then be biosynthesized from the hydroxyacids and glucose from depolymerized starch. Hence, this might be a tempting option than mineralization back to water and carbon dioxide, especially when the prices of agricultural products continue to increase. Moreover, recycling of PHAs is considered much easier than that of petroleum-based polymers such as polyethylene. This bodes well for the future of PHAs-based/starch composites.

Patel and Narayan [253] have successfully reviewed the sustainability of PHAs and starch blends. The study found that the carbon dioxide emission and energy use resulted from the PHAs’ production is almost the same or higher than those associated with petrochemical polymers. As/if the PHAs production becomes efficient, this is expected to change. The study has also highlighted that the carbon dioxide emissions as well as the energy use are substantially lower for starch, thermoplastic starch as well as starch blends than those of polystyrene or polyethylene [253].

Various studies discussing the modification of PHBV’s mechanical properties by the addition of nano clays have been reported. PHB and PHBV based nanocomposites were fabricated by adding Montmorillonites (MMTs) and Layered Double Hydroxides (LDHs) by solution casting [202,254,255,256,257,258] or by melt intercalation [184,205,208,259,260,261] to enhance PHAs’ mechanical properties.

Lim et al. was the first to fabricate PHB/MMT nanocomposites through solution casting [202]. In 2003, Maiti and Parkash [262] reported what is believed to be the first fabrication of PHB/Organo-Modified Montmorillonite (OMMT) nanocomposites. A higher storage modulus, that is around 40% higher than that of neat PHB was reported by the developed nanocomposite. Moreover, it has demonstrated an intercalated morphology while maintaining the biodegradability of PHB [262].

Melt extrusion was used by Maiti et al. [205] to prepare PHB-based nanocomposites reinforced using 2 wt.% organo-modified fluoromica or up to 3.6 wt.% MMT. Results showed that the nanocomposites’ storage modulus increased and better reinforcing was achieved in case of fluoromica than with MMT. This was proven by the higher amount of polymer degradation in the presence of MMT [205].

The influence of the incorporation of two nanoparticles, namely organomodified montmorillonite Cloisite^®^ 30B and a tubular like clay, halloysite (HNT), on the PHBV nanocomposites’ morphology, thermal as well as mechanical properties was evaluated by Carli et al. [209]. PHBV/Cloisite^®^ 30B demonstrated a structure that is partially exfoliated along with a few tactoids. On the other hand, a substantial enhancement in the modulus of elasticity and higher melting temperature were exhibited by the PHBV/HNT nanocomposites. Nonetheless, both of the impact strength and percentage elongation at break were reduced [209].

In another study [194], the incorporation of nanoclay Cloisite^®^ 30B resulted in no major effect on the PLA/PHBV/clay nanocomposites’ tensile strength and percentage elongation; however, the tensile modulus increased [194].

Parulekar et al. [259] used modified MMT with neopentyl(diallyl)oxytri (dioctyl)pyro-phosphato titanate to come up with PHB nanocomposites. Epoxidised natural rubber was used as an impact modifier and nanocomposites were prepared by extrusion followed by injection molding. Results showed that nanocomposites containing 5 wt% titanate-modified clay showed an improvement of around 400% in impact properties and a reduction of 40% in storage modulus when compared with unreinforced PHB [259].

The effectiveness of two commercial MMTs namely NA-MMT (Cloisite^®^ Na+) and the organo-montmorillonite, methyl tallow bis-hydroxyethyl quaternary ammonium-modified MMT (Cloisite^®^ 30B-M) as reinforcements to PHB matrix was investigated by Botana et al. [208]. The study showed that Young’s modulus of the nanocomposites increased. Nonetheless, there was no significant increase in the tensile strength as the exfoliation/intercalation ratio was not high enough. According to Pavlidou and Papaspyrides [263], the exfoliation/intercalation ratio is the main factor that determine the enhancement in nanocomposites’ mechanical properties. Intercalation can ensure that the Young’s modulus has increased; however, it is generally the exfoliation/intercalation ratio which determines the effect of the nano-additive on the tensile strength [263]. Cloisite^®^ 30B showed better particle exfoliation/intercalation, indicating better compatibility with the PHB matrix in that case [208].

Melt intercalation was used to prepare PHBV/Cloisite^®^ 30B nanocomposites [264]. Both X-Ray Powder Diffraction (XRD) and Transmission Electron Microscopy (TEM) analyses confirmed that the intercalated nanostructures were obtained. Clay addition (up to 3 wt.%) has successfully altered the mechanical properties. For instance, the modulus of elasticity increased significantly from 481 to 795 MPa as a result of the strong hydrogen bonding between PHBV and Cloisite^®^ 30B. On the other hand, tensile strength barely increased and there was a drop in the elongation at break from 8.5 to 5.6% [264].

In another investigation conducted by Chen and his colleagues [207], solution intercalation with 3 wt% filler content was used to come up with PHBV/OMMT nanocomposites. Results showed that there was a significant drop in tensile properties when higher filler loading (10 wt.%) was used due to clay aggregation. On the other hand, the addition of small quantities of OMMT was found to accelerate the overall rate of PHBV’s crystallization in a pronounced way [207].

Zhang et al. [265] prepared poly(3-hydroxybu-tyrate-*co*-3-hydroxyhexanoate) or PHB-*co*-PHH (Nodax^®^™) with up to 15% Cloisite^®^ 20A and Cloisite^®^ 25A. Results of the study showed that there was an increase in the elastic modulus, nonetheless, at higher clay loadings, Young’s modulus and tensile strength did not improve [265].

In a study conducted by Bruzaud and Bourmaud [254], Cloisite^®^ 15A was successfully used to come up with PHBV/organoclay nanocomposites using solution intercalation. Cloisite^®^ 15A content of 1, 2.5 and 5 (wt.%) was used in this investigation. Results showed an increase in the Young’s modulus, tensile stress and hardness with the increase of clay loading. This is attributed to the addition of stiff clay nanofillers into the PHBV matrix. Young’s modulus, tensile stress and hardness increased from 633 MPa, 5.9 MPa and 46 MPa for neat PHBV to 1677 MPa, 28.9 MPa and 88 MPa for the nanocomposite containing 5 wt.% Cloisite content, respectively. Furthermore, with the addition of only 2.5 wt.% clay loading, the Young’s modulus and hardness were enhanced by 66.9% and 67.4% respectively as compared with the neat PHBV. On the other hand, the elongation at break decreased from 3.3 to 1.4% with increasing clay loading. This indicates that the addition of clay led to an alteration of the plastic deformation of the matrix. All the nanocomposites exhibited greater thermal stability than neat PHBV [254].

The use of LDHs as a reinforcement to prepare PHA-based nanocomposites has been reported by various studies in literature [255,266,267]. The effect of these fillers on the mechanical properties of PHA has been reported by Dagnon et al. [267]. The study showed that the addition of stearic acid- modified Zn-AlNO3 LDH in PHBV (1–7 wt.%) resulted in more than a 10% improvement in the Young’s modulus; however, that was accompanied with a decrease in the elongation at break. Moreover, when up to 3 wt.% LDH was added, the strength increased, yet the strength decreased when further nanofiller was added, probably due aggregation [267].

Hsu et al. [255] were able to prepare PHB/Modified Layered Double Hydroxide (PMLDH) nanocomposites with 2 wt.% and 5 wt.%. Results showed that both the PMLDH content as well as the cooling rates affect the behavior of PHB and PHB/PMLDH composites. With the addition of 2 wt.% of PMLDH, the crystallization rate of the composite increased and the activation energy decreased. On the other hand, the crystallinity of PHB decreased and its activation energy increased when more PMLDH was added to the PHB. This is because the addition of more PMLDH limited the transport ability of the polymer chains [255].

Whilst there are several studies about the addition of inorganic nanofillers to reinforce the PHA matrix, there have been few reports about the use of organic nanofillers such as nanocellulose in PHAs.

Hydrolyzed tunicin cellulose whiskers were used to reinforce medium chain length PHAs [268]. Results showed a significant enhancement in the mechanical properties due to the formation of a transcrystalline network between the whiskers and the semi-crystalline matrix [268].

Using both, solution casting with N, N-dimethylformamide (DMF) as well as extrusion blending and injection molding of PHBV with freeze-dried nano whiskers, Jiang et al. [213] were able to prepare cellulose nano whisker/PHBV nanocomposites. The study reported that a homogeneous dispersion of the whiskers was attained and the cellulose nano whisker/PHBV nanocomposites exhibited enhanced tensile strength and Young’s modulus in case of solvent cast composites. However, the tensile strength reduced during freeze drying due to the agglomeration of whiskers [213].

A number of studies have reported the dynamic mechanical analysis of PHB and PHBV [269,270,271]. The main objective of such studies was to characterize viscoelastic properties as a function of temperature. It has been generally observed that the storage modulus for PHAs decreases with temperature. It has been also reported that PHB has a storage modulus in the range of 2500–3500 MPa at 20 °C while it is somewhat higher for PHBV [205,270,271]. Various studies in literature have reported the increase of both the storage modulus and the glass transition temperature due to the addition of nanofillers [205,213,269]. The reinforcement influence of nano clay additives reported to be become more prominent above the glass transition temperature, when the materials become soft. This is attributed to the polymer chains’ restricted movement [272].

Recently, melt extrusion was used to incorporate different amounts of Coffee Silverskin (CS), an agricultural residue, into a PHBV matrix plasticized by ATBC [273]. In order to examine the feasibility of the produced PHBV/CS-based bio composites to fabricate molded products, morphological, mechanical and thermal properties were examined. Results showed that as the content of CS increased, stiffness, heat deflection temperature and crystallinity all improved. Using injection molding, coffee capsules have been fabricated using the optimized formulation. At a temperature of 100 °C, the overall migration was below the limit (10 mg/dm^2^) required for plastic materials at food contact. The study concluded that CS can be efficiently used to prepare PHBV/CS based bio composites [273].

In another investigation [274], wood flour/PHA composites without additives were prepared using Fused Deposit Molding (FDM) 3D printing system based on micro screw extrusion. The study reported increased melting and crystallization temperatures of the composites. 3D-printed composites were free from warpage. This was attributed to the forming process under pressure and the wood flour/PHA blend. Flexural and tensile strengths of the composites were around 77.30 MPa and 38.70 MPa, respectively. Young’s modulus of the blend increased substantially with increasing the wood flour content. The study concluded that FDM has a great potential in the fabrication of 3D-printed bio-based composites [274].

**Figure 11:**
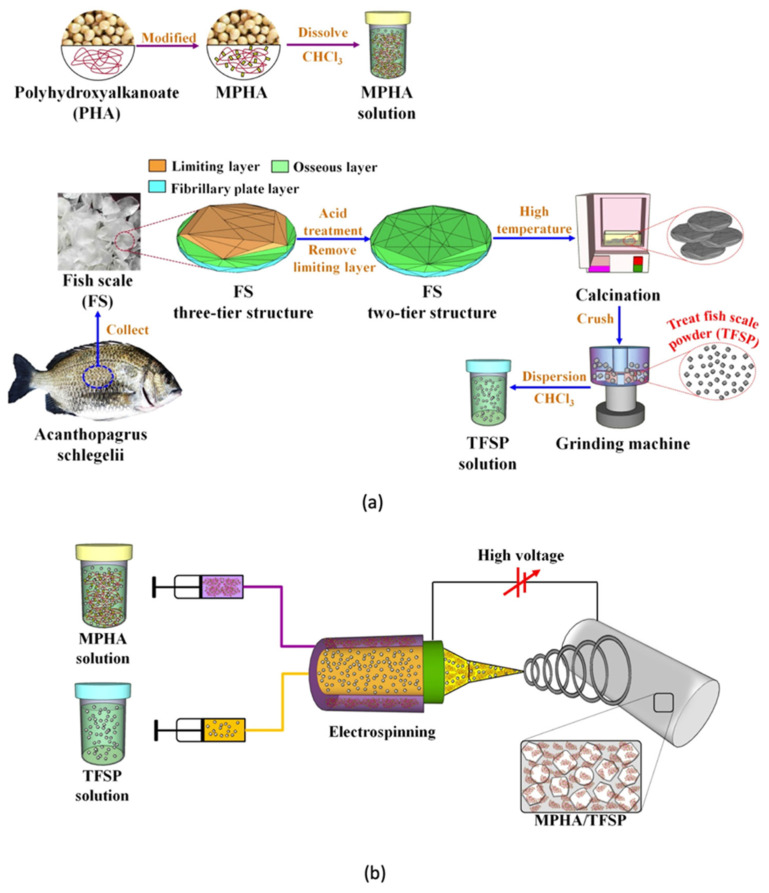
(**a**) The preparation process for the electrospinning modified PHA (MPHA) solution in addition to the treated fish scale powder (TFSP) and (**b**) The fabrication of electrospun MPHA/TFSP nanofiber. Reprinted with permission from American Chemical Society, 2021 [275].

Wu et al. [275] reported a biodegradable composite nanofiber consisting of PHA or modified PHA (MPHA) and treated fish scale powder (TFSP). Using grinding, the powder was prepared after the treatment of FSP with water, acid and heat (450 °C) to produce TFSP. After that, electrospinning (biaxial feed method) was used to produce composite nanofibers of TFSP/PHA and TFSP/MPHA. Figure 11 shows the preparation of the electrospinning solution as well as the fabrication process for the composite nanofibers. Results showed that the serum calcium to phosphorus ratio (Ca/P) of the TFSP was similar to that of the human bone. Moreover, MPHA/TFSP nanofibers exhibited more uniformity and were more strongly bonded in the matrix in comparison to PHA/TFSP composite. Increasing the content of TFSP led to improvement in the tensile strength at failure of the MPHA/TFSP composites. Percentage elongation at break decreased as the content of TFSP increased. The water contact angle reduced with increasing TFSP content in MPHA/TFSP and PHA/TFSP nanofiber membranes. The study reported that TFSP improved the hydrophilic effect of the PHA/TFSP and MPHA/TFSP nanofiber membranes providing a more suitable environment for cell growth [275].

PHBV based nanocomposites for bone filling and infection treatment were reported by Neto et al. [276]. The nanocomposites were fabricated from PHBV, nano diamond (nD) and nanohydroxyapatite (nHA) loaded with vancomycin (VC). They have prepared the nanocomposites using either a spray dryer or a rotary evaporator. SEM analysis showed a good distribution of the nHA particles. The nanoparticles exhibited a nucleating agent effect increasing the crystallinity of PHBV from 57.1% to 73.3%. The nanocomposites prepared by a spray dryer exhibited stronger interface as well as higher Tg than those prepared by the rotary evaporator. Furthermore, due to the addition of the nanoparticles, there was an increase by 34% of the flexural elastic modulus matching that reported for the human bone. After 22 days, the nanocomposites prepared by spray dryer and rotary evaporator reported VC release of 0.42 ± 0.05 mg and 1.38 ± 0.30 mg, respectively. These findings suggest that the developed nanocomposites can be promising candidates for bone defect filling [276].

Shahi and co-authors [277] used the polymer replication method to prepare porous ceramic nanocomposite scaffolds consisting different weight fractions of nano *β*-tricalcium phosphate (nano-*β*-TCP) (with a particle size of around 50–70 nm) coated with PHB for 30 and 60 s. Results showed that the fabricated scaffold with 50 wt.% nano-*β*-TCP and a coating time of 30 s reflected desirable properties in bone tissue engineering. After examining the bioactivity of this scaffold, bone-like apatite layers were found to be well formed on the nanocomposite scaffold. This nanocomposite scaffold is believed to have a good potential for applications in bone tissue engineering [277].

Recently, Jo et al. [216] have successfully improved the mechanical properties of PHA based composites through the addition of surface-modified CNCs via melt-extrusion. To obtain hydrophobically treated CNCs, double silanization using tetraethyl orthosilicate (TEOS) and methyltrimethoxysilane (MTMS) was conducted. The addition of double silanized CNCs acted as a nucleating agent and improved the elongation at break up to 301% with a minor drop in the tensile modulus. The produced composites are likely appropriate for future utilization in commercial applications demanding high ductility [216].

Table 14 shows the impact of different PHAs blends and nanocomposites at different concentrations on the mechanical properties.

### 3.3. Features of Various PHAs Blends and Nanocomposites

Blending PHB with PCL [195] as well as blending PHBV with PBS [193] and PLA and PBS [144] have been found to enhance the ductility. Improvement in the tensile strength for PHB and the PHBV’s tensile strength in the quasi-static tensile test have been found to enhance due to blending with PCL [197] and PBS [193], respectively. Complete degradable blends were obtained via mixing PHB with PCL [186] as well as PHBV with PLA and PBS [144]. Blending PHB with PETG [192] results in a significant improvement in the flexural modulus.

The incorporation of MWCNTs into PHBV improves the thermal stability and crystallinity of PHBV [203,204]. Blending PHBV with CNTs and CNF [215] has been proven to enhance the barrier and mechanical properties of PHBV. Improvement in the crystallinity of P3HB-*co*-4HB [218] and mcl-PHAs [219] were observed after blending with CNFs. A significant enhancement in the mechanical and thermal properties was reported as a result of blending PHB with MMT [205]. Table 15 shows the advantages and applications of selected studies on PHAs blends and nanocomposites.

## 4. Trends of PLA and PHAs Applications

### 4.1. PLA Foams, 3D-printed Scaffolds and Flame Retardancy

**Figure 12:**
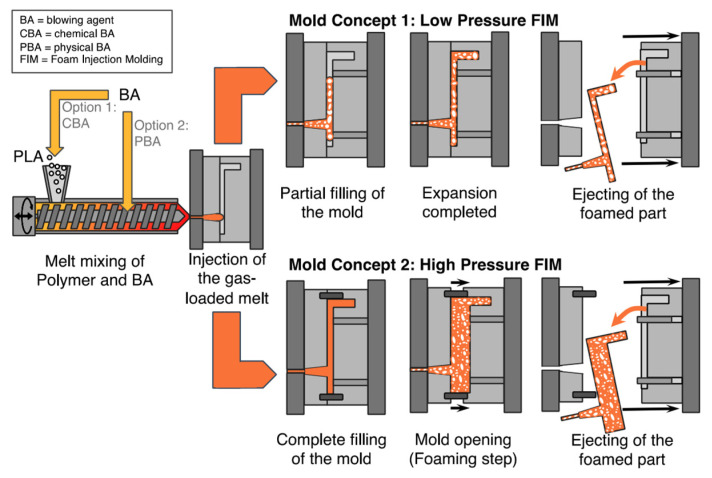
The concept of foam injection molding [23].

**Figure 13:**
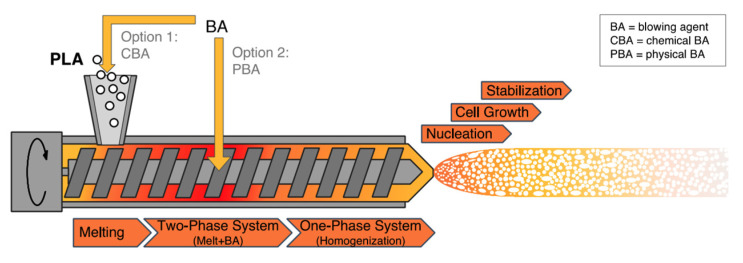
The concept of foam extrusion [23].

**Figure 14:**
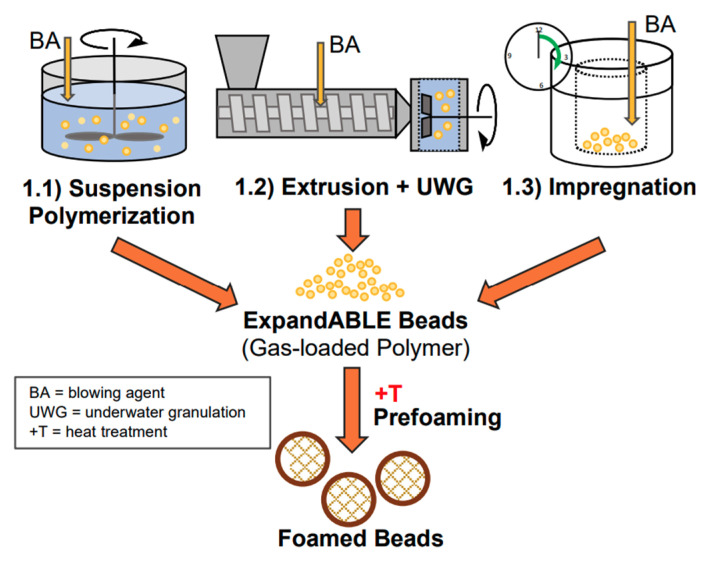
The various methods for the production of expandable bead foams [23].

One of the ideal materials for various packaging applications is PLA foam. PLA foams are becoming increasingly desired as renewable biopolymer alternatives to petroleum-based polymer foams. This is due to their light weight and good cushioning properties. Polymers foams are produced using different blowing agents, which can be categorized as chemical and physical. The production of foam cells under the impact of pressure/temperature release is done via chemical blowing agents. On the other hand, physical foaming of polymers and composites, results in the production of cellular structures with cell sizes smaller than 10 µm and cell densities greater than 10^9^ cells/cm^3^, known as microcellular foams [278,279]. PLA foams can be produced via batch processing, foam injection molding, bead foaming as well as foam extrusion. Foam PLA/natural (lignocellulosic) fiber-reinforced composites are produced mainly using foam injection molding and extrusion. Figure 12, Figure 13 and Figure 14, show the concept of foam injection molding, foam extrusion and bead foaming, respectively. Such foams exhibit thermal and mechanical properties comparable to those of the currently used petroleum-based foams [23,280]. An improvement of the morphology of the foam cell via modification of polymer melt viscosity is believed to be feasible through the addition of lignocellulosic fibers. For example, the incorporation of lignocellulosic fibers can lower the cell size and increase foam cell density [279,281,282,283,284,285,286]. Moreover, PLA/natural (lignocellulosic) fiber-reinforced composites were found to enhance specific tensile and flexural moduli. Furthermore, changes in PLA crystallinity prompted by the incorporation of fibers can potentially alter the foam cell characteristics of PLA composites [287].

In one study [287], microcellular injection molding process was used to produce foamed flax fiber reinforced PLA composites at three various flax concentrations (1, 10 and 20 wt.%). Neat PLA was reported to have an average cell size of 8.4 µm. On the other hand, the cell sizes of PLA/flax composites dropped by around 11%, 47% and 67% at 1, 10 and 20 wt.% flax fiber, respectively. The study has also reported an increase in the specific tensile modulus by around 3%, 10% and 22% for the foamed composites at 1, 10 and 20 wt.% fiber loadings, respectively. This was attributed to the flax fibers’ higher modulus in comparison to that of PLA as well as to the restraining impact of fillers on the movement of polymer chain yielding to enhanced stiffness [287].

An investigation of the impact of the addition of willow fiber at 20 wt.% and 30 wt.% concentrations on the mechanical properties of foamed PLA composites was done by Zafar et al. [288]. Neat PLA was reported to have an average cell size of about 33.7 µm. However, the cell sizes of PLA/flax composites dropped to about 20.6 µm at 20 wt.% and 18.1 µm at 30 wt.% willow fiber, respectively. The study reported a slight increase in the flexural modulus and the maximum value was corresponding to that of PLA/30 wt.% willow fiber composite. The specific notched impact strength of PLA/flax composites increased by about 16% at 20% and 45% at 30 wt.% willow fiber, respectively. Moreover, the degree of crystallinity has also increased with the addition of willow fiber [288].

In addition to their use in packaging applications, foamed PLA products have been also utilized in tissue engineering and drug release. PLA is often used in bone or cartilage tissue engineering in the form of 3D-printed scaffolds. One of the most widely used technologies for PLA is additive manufacturing. Generally, a precise control of the 3D printer is essential for the quality of 3D-printed products. A wide range of 3D printers such as Ultimakers and Robo can be used to 3D print PLA filaments [289]. The strength of PLA’s printed parts depends mainly on the direction of printing. Therefore, the following points should be given special considerations during the 3D printing. Force application’s direction shall not be perpendicular to the printing layer and when printing complex parts, the outer shell thickness, printing pattern, density and the interconnecting parts must be given great considerations as they can lead to premature brittleness. Another important point is to make sure that the platform holds the 3D-printed part firmly so as to avoid the printed spot from being distorted or pulled out. Therefore, it is recommended to use a painter’s tape to hold the platform firmly in position [290]. Using a painter’s tape will also make it easy to take off the PLA’s 3D-printed part as the printed object will stick to the surface of the painter’s tape. This will avoid damaging the object when the 3D printing is finished. Another benefit of using a painter’s tape is that it can help in avoiding warpage, particularly for semicrystalline PLA which can undertake substantial irregularity in shrinkage when molten PLA’s layers are laid continuously. Moreover, heating the platform can also create a sticking effect. Nevertheless, the platform’s temperature shall be kept within the limits that will not cause polymer softening or degradation. The recommended platform and printing temperatures for PLA are 60 °C and 210 °C, respectively. Exposing the PLA filament to high temperatures and moisture can lead to degradation, depolymerization and/or chain scissioning. Therefore, it is recommended to keep the PLA filament stored in a securely sealed condition at a relative humidity less than 10%. Unsealing of the PLA filament is recommended just before the start of printing.

**Figure 15:**
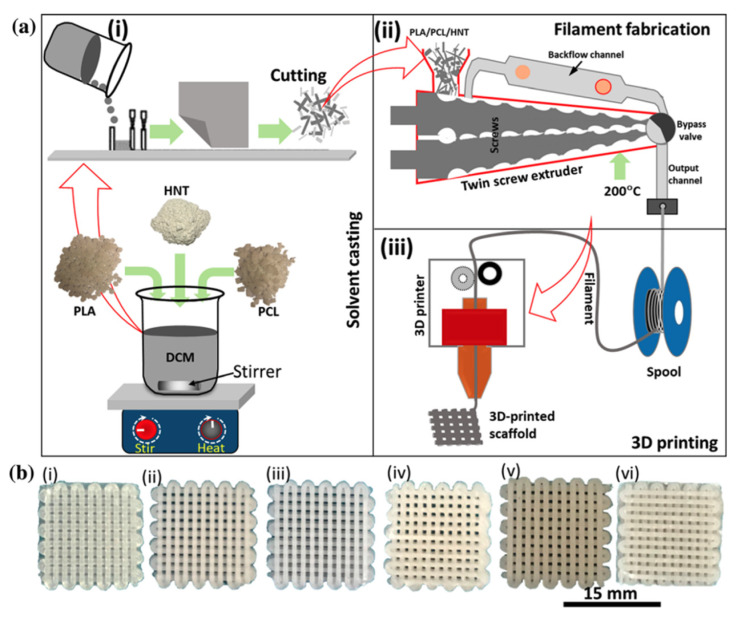
(**a**) Schematic of filament fabrication and 3D printing of scaffolds and (**b**) optical images of various scaffolds ((**i**) for neat PLA, (**ii**) PLA/PCL (50/50 wt.%), (**iii**) PLA/PCL/HNTs (50/50/1 wt.%), (**iv**) PLA/PCL/HNTs (50/50/3 wt.%), (**v**) PLA/PCL/HNTs (50/50/5 wt.%) and (**vi**) PLA/PCL/HNTs (50/50/7 wt.%)) [171].

Different studies in literature have reported producing medical implants at a more affordable cost using 3D printing of PLA. Conventional manufacturing techniques such as casting or forging are time consuming and most of the time fail to meet the patients’ needs. Three-dimensional printing of scaffolds is one of the mostly suggested medical applications for PLA. For these 3D-printed scaffolds to be able to offer an interconnected network for cell growth as well as transportation of nutrients and waste generated from metabolism, they must meet certain mechanical properties, structural features and durability. Furthermore, these scaffolds are biocompatible with controlled rate of degradation Therefore, in the long term, there should be no problem for these scaffolds to adhere and match with the tissues [291]. According to Kikuchi et al. [292], to meet the functional requirements of scaffolds, bioactive ceramics such as beta-tricalcium phosphate, hydroxyapatite and calcium phosphate are incorporated with PLA [292]. Niaza et al. [293] investigated 3D printing of porous scaffolds of compounded hydroxyapatite and PLA at an average particle size of 90 nm and 1 μm. FFF technique with a nozzle temperature of 220 °C was used. Results suggested that the modulus of elasticity for PLA with micro-sized hydroxyapatite and nano-sized hydroxyapatite were 2.8 and 4.0 GPa respectively. Knowing that the Young’s modulus for the trabecular bone is in the range of 3–5 GPa, it is then very likely to use the 3D-printed PLA- nanosized hydroxypatite composite bone scaffolding as an alternative to original bone as implants. Moreover, the formation of a high-porosity structure due to the sintering between the layers during 3D printing makes PLA-hydroxyapatite composite a good feasible substitute to original bone [293]. Generally, high porosity is linked to a weaker structure, which in this case weaker PLA-hydroxyapatite composite, nonetheless, such a condition is safe with the addition of nano-sized hydroxyapatite. Various porosities of 3D-printed PLA scaffold structures were studies and compared by Gregor et al. [294]. The investigators were able to 3D print scaffolds with various geometrical structures using FDM. Two types of scaffolds of the defined shape and engineered inner structure that provides regular and sufficient porosity have been successfully printed. The designed 3D-printed scaffolds were subjected to osteosarcoma cells proliferation experiment and mechanical testing. Results suggest that the proliferation of both types of 3D-printed scaffolds with porosity values of 30% and 50% was satisfying with good mechanical durability [294]. Figure 15 shows a schematic of production of filament as well as 3D-printed scaffolds using FFF. According to Alam et al. [171], the process involves (i) solvent casting followed by (ii) filaments fabrication via extrusion and finally (iii) 3D printing of scaffolds.

Various applications (e.g., construction, automobile and electronics) requires high criteria for dripping combustions and flammability, which cannot be satisfied by neat PLA. Therefore, there have been some attempts to enhance PLA’s flame retardancy for compact and foamed forms of PLA. In one study [295], an enhancement in compact PLA’s flame retardancy was achieved by a synergistic mixture of ammonium polyphosphate (APP) with expandable graphite (EG), an eco-friendly flame retardant. With 15% of this intumescent flame retardant (APP/EG = 3:1), there was an increase in the Limiting Oxygen Index (LOI) from 22 to 36.5. The UL-94-V-0 classification was also reached [295]. In another study [296], the same burning behavior was reported using 30% of a mixture (3:2) of a novel hyperbranched polyamine charring agent (HPCA) together with APP. Tang et al. [297] reported good flame retardancy, anti-dripping effects and high LOI values with synergistic combinations of expanded graphite and aluminum hypophosphite [297]. Other studies were done to enhance foamed PLA’s flame retardancy with a phosphorous containing flame retardant, as well as graphene [298] or starch [299] as a charring agent. UL-94-V-0 classification was reported. Moreover, LOI was significantly increased and anti-dripping effects were observed.

Vadas and coworkers were able to incorporate a bio-based flame retardant into a PLA extrusion foam [300]. A combination of APP as an intumescent flame retardant and flame retardant treated cellulose (surface treatment with boric acid and diammonium phosphate) as a bio-based charring agent was used to lower PLA foams’ flammability. A multifunctional epoxy-based chain extender was utilized and, even at elevated additive loadings, a substantial expansion with void fractions higher than 90% was attainable with carbon dioxide as a blowing agent. With an additive content lower than 20%, superb flame retardancy (LOI of 31.5% and UL-94 V-0) was reported. Moreover, compared to the compact materials, the flame retardant synergism was less noticeable in the expanded foams. This is attributed to the enlarged contact surface as well as the flame retardant’s lower volume concentration [298,299].

In another investigation [301], a novel flame retardant and toughened bio-based (PLA)/glycidyl methacrylate-grafted natural rubber (GNR) composite was reported. The interfacial compatibility between PLA/GNR matrix and the charring ability of the PLA/GNR/SiAHP composites as well as the modified aluminum hypophosphite by silane (SiAHP) was enhanced to a certain extent due to the surface modification of AHP. The flame retardancy and toughness of the PLA/GNR/SiAHP composites were slightly greater than those of PLA/GNR/AHP composites. UL-94 V-0 rating and LOI of 26.50% were reported. The promising flame retardancy of the PLA/GNR/SiAHP composites was suggested to be due to the synergistic effect including condensed and gaseous phase flame-retardant mechanisms. High-performance flame-retardant PLA/GNR/SiAHP composites have great potential applications as alternatives to petroleum-based polymers in the building and automotive interior sectors [301].

**Figure 16:**
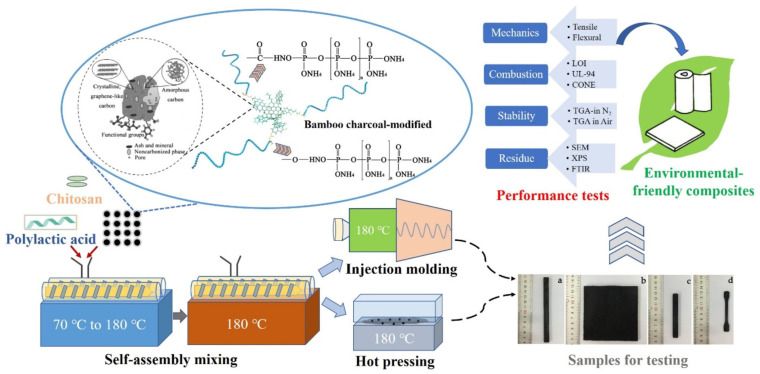
Composite preparation and testing as reported by Li et al. [302].

Li et al. [302] developed a cooperative flame-retardant system based on natural intumescent-grafted bamboo charcoal (BC) and chitosan (CS) for PLA. Figure 16 shows a schematic diagram of the composite preparation and testing. The composite demonstrated minimal decline in strength properties and enhanced flame retardancy. CS as an adhesion promoter enhanced the interfacial compatibility between PLA and graft modified bamboo charcoal resulting in improving the tensile properties by 8.42% and 11.11%, respectively for the Young’s modulus and tensile strength. The study found that CS endorses the reorganization of the internal crystal structure. At 3 wt.% CS and 30 wt.% graft-modified bamboo charcoal, the composite’s crystallinity was reported to be 43 times that of neat PLA. Flammability tests (UL-94 V-0 rating and LOI of 33.6 vol.%) showed a substantial enhancement in flame retardancy. The reported composite is claimed to meet the requirements for strong, biodegradable and non-toxic PLA packaging products [302].

### 4.2. PHAs in Active Food Packaging

**Figure 17:**
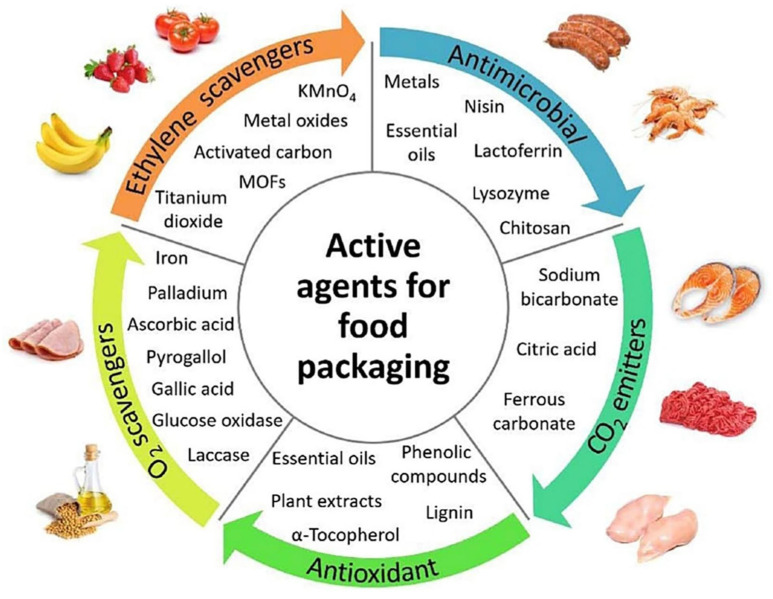
Bioactive agents for smart food packaging. Reprinted with permission from Elsevier, 2020 [303].

**Figure 18:**
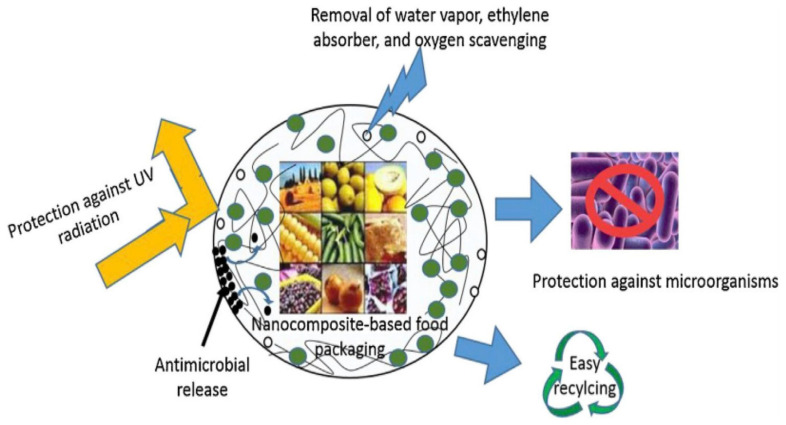
Active food packaging based on bio nanocomposites. Reprinted with permission from Elsevier, 2020 [303].

Technologies that are developed to enhance shelf life, sensory properties and keeps the packaged food safe from mechanical damage as well as microbial contamination are referred to as active food packaging. Bioactive agents (Figure 17) have been used to create edible films with induced desirable functionalities. Figure 18 shows a schematic of active food packaging based on bio nanocomposites with outstanding preservation capability against pathogens and UV irradiation [303]. In one study [304], poly(3-hydroxyalkanoate-*co*-3-hydroxyalkanoate) (PHAE) (an mcl-PHA) was coated with zosteric acid (nontoxic, antifouling agent) to come up with a PHA-based active food packaging. Exposing the coated PHAE to sewage sludge showed no signs of microbial growth [304]. In another work [305], Azotobacter chroococcum 23 was used to synthesize PHB in order to develop a PHA-based active food packaging. Antimicrobial agents, namely chemically synthesized benzoic acid and natural Silbiol were added to the PHB films and PHB-coated paper surface. The PHB films and PHB-coated paper surface exhibited no major antimicrobial activity against Gram-negative and Gram-positive bacterial strains [305]. For the purpose of preventing the antimicrobial agents such as benzoic acid from migrating into the food from the food packaging, Kwiecien et al. [306] produced an active food packaging system based on preservative-oligo (3-HB). Different concentrations of the antimicrobial agent vanillin (4-hydroxy-3-methoxybenzaldehyde) was added to the PHB solution [307]. The antimicrobial potential of the developed films was tested against a variety of bacterial strains and fungi. Both thermal and mechanical properties of the produced PHB films with and without vanillin were investigated and analyzed. The study concluded that in order to demonstrate antimicrobial activity, the minimum concentration of vanillin required is ≥50 μg/g PHB for fungi and ≥80 μg/g PHB for bacteria. Furthermore, the elongation at break for the PHB-vanillin exhibited a small increase when compared to that of PHB film. Nonetheless, both of the modulus of elasticity and tensile strength decreased. The rate of migration of vanillin at 37 °C into 50% ethanol and distilled water were 71.736 mg/mL and 65.54 mg/mL, respectively. This might be attributed to the higher temperature and faster migration of vanillin into 50% ethanol than distilled water [307].

## 5. Conclusions and Future Insights

PLA is a biodegradable polymer which has a tremendous advantage in overcoming the pollution of plastics following their disposal. PLA can be also produced from agricultural sources which makes it an attractive option over petroleum-based polymers. Due to their nontoxicity, biodegradability and hydrophobicity, PHAs have been widely used in many applications worldwide. Nonetheless, the poor toughness of PLA hinders the widespread use of this polymer in many other applications. PLA-based blends and nanocomposites have been found efficient in enhancing the mechanical properties of PLA. Recently and due to the raised awareness of the environmental concerns, the production of PHAs has been observed to increase significantly. However, the high production cost of PHAs is the main obstacle for expanding their productions to the commercial scale. Blending PHAs with other polymers as well as PHAs based nanocomposites have been reported to be a good option to increase their flexibility and mechanical properties while enhancing/maintaining its biodegradability. The increasing number of additives played a significant role in the development of PLA’s and PHAs’ physical properties to a high level of performance. Yet, the market for bio-based plastics’ additives still lacks solutions for significant properties. For example, investigating the migration behavior of the nano clays, nanoparticles and nanofillers incorporated with PHAs is crucial prior to the use of PHA-based nanocomposites in food packaging. Finally, modified PLA and PHAs have high potential to be used in applications such as drug delivery, tissue engineering, food packaging and bone scaffolds. Furthermore, they have demonstrated good feasibility in structural, automobile, personal care and electronic applications. The wide range of promising properties that these two bio-based polymers offer after modification have paved the way to justify their utilizations today as green biodegradable substitutes to petroleum-based plastics.

## Figures and Tables

**Table 1:** Some of the α-Hydroxycarboxylic acid derived polyesters along with their manufacturers and applications. Adapted with permission from Elsevier, 2015 [15,28,29].

Bioplastic	Company	Country	Commercial Name	Applications/Notes
PLA	NatureWorks LLC	USA	-Ingeo™ 8000 series, 8052D. -Ingeo™ 7000 series, 7001D TDS and 7032D TDS. -Ingeo™ 6000 series, 6060D TDS, 6201D TDS, 6202D TDS, 6204D TDS, 6251D TDS, 6252D TDS,6302D TDS, 6400D TDS, 6751D TDS, 6752D TDS. -Ingeo™ 4000 series, 4032D TDS, 4043D TDS, 4060D TDS. -Ingeo™ 3000 series, 3001D SDS, 3052D SDS, 3251D SDS, 3801X SDS. -Ingeo™ 2000 series, 2003D TDS. -Ingeo™ 3D850.	-Foams. -Bottles (7001D TDS and 7032D TDS). -Nonwovens (6060D TDS, 6202D TDS, 6251D TDS, 6252D TDS, 6302D TDS, 6751D TDS, 6752D TDS). -Apparel (6201D TDS, 6204D TDS). -Home textiles (knitted and woven) (6201D TDS, 6202D TDS, 6204D TDS, 6400D TDS). -Cards, folded cartons and films (4032D TDS, 4043D TDS, 4060D TDS). -3D printing (3D850, 4043D). -Durable goods (3001D SDS, 3052D SDS, 3251D SDS, 3801X SDS). -Service war (2003D TDS, 3001D TDS, 3052D TDS, 3251D TDS). -Food packaging (2003D TDS).
PLA, PDLA	Total Corbion PLA	The Netherlands	-Luminy^®^ PLA (L175, L130, L105, LX575, LX530, LX175, LX975, LX930, D120, D070)	-High heat PLA for demanding applications (L175, L130, L105, LX575, LX530). -Standard PLA for general purpose applications (LX175). -Low heat PLA for usage in seal layers (LX975, LX930). -PDLA utilized to produce full stereocomplex compounds or used as a nucleating agent (D120, D070).
PLLA	Purac	The Netherlands	-Purasorb^®^ (PL 18, PL 24, PL 32, PL 38, PL 49, PL 65, PL 10).	-Medical equipment.
PLLA, scPLA	Tejin	Japan	-Biofront^®^ (HL L201 (PLLA), J20 (scPLA), J201 (scPLA)).	-Eyeglass frames, sheets, films, fibers, injection molding, medical care, automobiles, electronics, construction and packages.
Amorphous PLA	Toyobo	Japan	-Vyloecol series (BE-400, BE-600, BE-910, HYD-306, BE-450, BE-410, HYD-006).	-Adhesive, paint, printing ink. -BE-400 in the form of pellet, used as agent for different coating and is a general-purpose resin. -BE-600 in the form of sheet, used as anchor coating for printing ink and vapor deposition films.
PDLA	Purac	The Netherlands	-Purasorb^®^ -PD 24 -PD 38 -Purapol^®^	-Medical equipment (Purasorb^®^). -Nucleating agents for PLLA (Purapol^®^).
PDLLA	Evonik	Germany	-R 202 H -R 203 H -R 202 S -R 203 S -R 205 S -R 207 S	-Medical equipment (R 207 S) and drug delivery.
PLDLLA	Purac	The Netherlands	-Purasorb^®^ (PLDL 8038, PLDL 8058, PLDL 7028, PLDL 7038, PLDL 7060).	-Medical equipment.
PLDA	Purac	The Netherlands	-Purasorb^®^ (PLD 9620, PLD 9655).	-Medical equipment.
PLA (Nature- Works)/ copolyester blend	FKuR Kunststoff GmbH	Germany	-Bio-flex^®^ (Bio-flex^®^ A4100 CL, Bio-flex^®^ F 1110, Bio-flex^®^ F 1130, Bio-flex^®^ F 1137, Bio-flex^®^ F 2110, Bio-flex^®^ F 2201 CL, Bio-flex^®^ F 6510, Bio-flex^®^ F 6513, Bio-flex^®^ F 6611, Bio-flex^®^ S 5630, Bio-flex^®^ S 6540, Bio-flex^®^ S 9533).	-Flower wrapping, blown film extrusion and packaging (Bio-flex^®^ A4100 CL). -Waste bag, air pillow and carrier bag (Bio-flex^®^ F 1130). -Shopping bags (Bio-flex^®^ F 1137). -Waste bag, netting and deep freeze packaging (Bio-flex^®^ F 2110). -Film (Bio-flex^®^ F 2201 CL). -Multi-layer films (Bio-flex^®^ A4100 CL and Bio-flex^®^ F 2201 CL). -Straws, mugs and ball pen (Bio-flex^®^ F 6510). -Thermoforming (Bio-flex^®^ F 6611). -Injection molding (Bio-flex^®^ F 6513). -Thermoformed inlay (Bio-flex^®^ S 5630). -Cosmetic jars (Bio-flex^®^ S 6540 and Bio-flex^®^ S 9533).
PLA/polyether *co*polymer	Toray Industries	Japan	-Ecodear^®^ (V554R10, V554X51, V554X52, V751X52, V751X53, V911X51).	-Bags, films, fibers, packaging, personal care, accessories, office supplies and electronics.

Abbreviations: PLA, poly(lactic acid); PDLA, poly (D-lactic acid); PLLA, poly(L-lactide); scPLA; sterecomplex PLA; PDLLA, poly (D, L-lactide); PLDLLA, poly(L-lactide-*co*-D,L-lactide); PLDA, poly(L-lactide-*co*-D-lactide).

**Table 2:** Some of the commercial PHAs along with their manufacturers and applications. Adapted with permission from Elsevier, 2015 [15].

Bioplastic	Company	Country	Commercial Name	Applications/Notes
PHB	Mitsubishi Gas Chemical Company Inc.	Japan	-Biogreen^®^	-Cast films and natural latex gloves.
PHB	PHB Industrial S/A	Brazil	-Biocycle™ (B1000, B18BC-1, B189C-1, B189D-1)	-Medical devices, films and disposables).
PHB and PHBV	Biomer Inc.	Germany	-Biomer^®^300 (P300E, P300F)	-Extrusion (P300E) -Extrusion and food contact (P300F).
PHBV and PHBV/PLA	Tianan Biologic, Ningbo	China	-Enmat™ (Y1000, Y1010, Y1000P, Y3000, Y3000P, F9000P).	-Thermoforming, nonwovens and fiber, injection molding, extrusion and water treatment.
P4HB	Tepha, Inc.	USA	-TephaFLEX^®^	-Surgical absorbable films and sutures.
PHBHH_x_	Kaneka Co.	Japan	-Kaneka PHBH -Aonilex^®^	-Foams, fibers, interior automotive materials, electrical equipment, sheets and injection molding. -Containers, bottles, interior automotive materials and electrical equipment.
PHBHH_x_	Danimer Scientific	USA	-Nodax™	-Coating, laminates, non-woven Fibers and packaging.
P3HB4HB	Tianjin Green Bio- Science Co./DSM	China/The netherlands	-GreenBio^®^	-Films for wrapping, laminating film, fresh film, heat shrinkable film, garbage bags, food packaging, shopping and gift bags.
Several PHAs	CJ CheilJedang Corporation	South Korea	-CJ PHA^®^	-Rigid packaging, 3D printing, paper coating, agriculture and flexible packaging.
Several PHAs	Alterra Holdings	USA	-TerraBio^®^	-Paper coating, packaging, utensils, straws and disposals.

Abbreviations: PHB, polyhydroxybutyrate; PHBV, poly(3-hydroxybutyrate-*co*-3-hydroxyvalerate);PLA, poly(lactic acid); P4HB, poly(4-hydroxybutyrate); PHBHH_x_, poly(3-hydroxybutyrate-*co*-3-hydroxyhexanoate); P3HB4HB, poly(3-hydroxybutyrate-*co*-4-hydroxybutyrate).

**Table 3:** Mechanical, thermal and physical properties of some PLA produced by NatureWorks LLC [53,54].

Properties/Applications	Ingeo™ 2003D	Ingeo™ 3052D	Ingeo™ 3801X	ASTM Method
Specific Gravity	1.24	1.24	1.25	D792
Melt Flow Rate, g/10 min (210 °C, 2.16 Kg)	6	14	8	D1238
Relative viscosity	NP	3.3	3.1	-
Clarity	Transparent	Transparent	Opaque	-
Tensile strength at break, psi (MPa)	7700 (53)	NP	NP	D882
Tensile yield strength, psi (MPa)	8700 (60)	9000 (62)	3750 (25.9)	D882
Tensile modulus, Kpsi (GPa)	500 (3.5)	NP	432 (2.98)	D882
Flexural Strength, psi (MPa)	NP	15,700 (108)	6400 (44)	D790
Flexural Modulus, psi (MPa)	NP	515,000 (3600)	413,000 (2850)	D790
Tensile elongation, %	6.0	3.5	8.1	D882
Notched Izod impact, ft-lb/in (J/m)	0.3 (16)	0.3 (16)	2.7 (144)	D256
Heat distortion temperature (°C)	55	55	65 (at 66 psi) 140 (at 16.5 psi)	E2092
Melt temperature (°C)	210	200	188	-
Crystallinity melt temperature (°C)	NP	145–160	155–170	D3418
Glass transition temperature (°C)	NP	55–60	45	D3418
Applications	-Designed for fresh food packaging and food service ware applications such as: dairy containers, food service ware, transparent food containers, hinged ware and cold drink cups.	-Designed for injection molding applications that require clarity with heat deflection temperatures lower than 49 °C. -Applications include: cutlery, cups, plates and saucers as well as outdoor novelties.	-Designed for non-food contact injection molding applications that require opaque molded parts with heat deflection temperatures between 65 °C and 140 °C.	-

NP: Not provided.

**Table 4:** Mechanical properties of poly(L-lactic acid); poly(styrene) and poly(ethylene terephthalate) [54,56].

Polymer	Tensile Strength (MPa)	Tensile Modulus (GPa)	Percentage Elongation	Notched Izod (J/m)
PLLA	59	3.8	4–7	26
PS	45	3.2	3	21
PET	57	2.8–4.1	300	59

Abbreviations: PLLA, poly(L-lactic acid); PS, poly(styrene); PET, poly(ethylene terephthalate).

**Table 5:** Various plasticizers and their effects on the different mechanical properties of PLA along with their applications.

Plasticizer	Plasticizer’s Concentration (wt.%)	PLA’s Type and Reference	Tensile Strength (MPa)	Young’s Modulus (MPa)	Percentage Elongation	Charpy Impact, (MJ/mm^2^)	Application	Comments
Lactide	- 25.5% - 19.2% - 17.3% - 1.3%	PLA, in the form of films [58].	- 16.8 - 29.2 - 15.8 - 51.7	- 232 - 658 - 820 - 1993	- 546% - 536% - 288% - 3.00%	-	General Packaging.	Degradation increased with increasing the content of plasticizer.
PEG	- 0% - PEG 1500 (2.5%) - PEG 1500 (5%) - PEG 1500 (10%)	PLA (92% L-lactide and 8% meso-lactide) [59].	- 58 - 50 - 44 - 28	- 3800 - 3200 - 2500 - 1200	- 3% - 4% - 7% - 40%	- 32 ^b^ - 29 - 31 - 80	Applications demanding higher impact resistance and flexibility.	-
	- 0% - m-PEG (10%) - m-PEG (20%) - PEG 400 (10%) - PEG 400 (20%)	PLA (92% L-lactide and 8% meso-lactide) [60].	-	- 2050 - 1571 - 1124 - 1488 - 976	- 9% - 18% - 142% - 26% - 160%	-	-	Biocompatible plasticizers.
	- 0% - PEG 400 (5%) - PEG 400 (10%) - PEG 400 (12.5%) - PEG 400 (15%) - PEG 400 (20%) - PEG 1500 (5%) - PEG 1500 (10%) - PEG 1500 (12.5%) - PEG 1500 (15%) - PEG 1500 (20%) - PEG 10,000 (5%) - PEG 10,000 (10%) - PEG 10,000 (15%) - PEG 10,000 (20%)	PLA [61].	- 66.0 - 41.6 - 32.5 - 18.7 - 19.1 - 15.6 - 52.3 - 46.6 - 18.5 - 23.6 - 21.8 - 53.9 - 48.5 - 42.3 - 22.1	- 3300 - 2500 - 1200 - 500 - 600 - 500 - 2900 - 2800 - 700 - 800 - 600 - 2800 - 2800 - 2500 - 700	- 1.8% - 1.6% - 140% - 115% - 88% - 71% - 3.5% - 5.0% - 194% - 216% - 235% - 2.4% - 2.8% - 3.5% - 130%	-	Medical, personal care and food packaging applications.	-
	- 0% - PEG 200 (10%) - PEG 400 (10%) - PEG 400 (20%) - PEG 1000 (10%) - PEG 1000 (20%) - PEG 1000 (30%)	PLA (92% L-lactide and 8% D-lactide) [62].	- 64.0 - 30.0 - 39.0 - 16.0 - 39.6 - 21.6 - 4.70	- 2840 - 1700 - 1920 - 630 - 1970 - 290 - 420	- 3.0% - 2.0% - 2.40% - 21.2% - 2.7% - 200% - 1.50%	-	Food packaging Applications.	The plasticizers used are food packaging approved.
	- 0% -PEG 600 (5.0%) - PEG 600 (7.50%) - PEG 600 (10.0%) - PEG 600 (12.50%)	PLA [63].	- 25.5 - 19.3 - 17.5 - 18.5 - 19.7	-	- 64% - 67.0% - 360% - 427% - 622%	-	-	PPGs increased the ability of PLA to plastically deform in a more efficient way than PEG.
Glucose monoesters	- 0% - 2.5% - 5% - 10%	PLA (92% L-lactide and 8% meso-lactide) [59].	- 58 - 52 - 47 - 39	- 3800 - 3200 - 3000 - 2550	- 3% - 5% - 6% - 12%	- 32 ^b^ - 23 ^b^ - 24 ^b^ - 18 ^b^	Applications requiring higher impact resistance and flexibility.	-
Partial fatty acid esters	- 0% - 2.5% - 5% - 10%		- 58 - 52 - 48 - 44	- 3800 - 3450 - 3100 - 3000	- 3% - 14% - 7% - 8%	- 32 ^b^ - 25 ^b^ - 28 ^b^ - 22 ^b^		
Oligomeric lactic acid	- 0% - 10% - 20%	PLA (92% L-lactide and 8% meso-lactide) [60].	-	- 2050 - 1256 - 744	- 9% - 32% - 200%	-	-	Biocompatible Plasticizers.
ATBC	- 0% - 5% - 10% - 12.5% - 15% - 20%	PLA [61].	- 66.0 - 53.4 - 50.1 - 17.7 - 21.3 - 23.1	- 3300 - 3200 - 2900 - 100 - 100 - 100	- 1.8% - 5.1% - 7.0% - 218% - 299% - 298%	-	Medical, personal care and food packaging applications.	ATBC is derived from naturally occurring citric acid. It is also non-toxic and has been approved for use in personal careand medical applications.
PBOH	- 0% - 10% - 20% - 30%	PLA (92% L-lactide and 8% D-lactide) [62].	- 64.0 - 56.3 - 30.2 - 25.2	- 2840 - 2350 - 350 - 300	- 3.0% - 3.00% - 302.5% - 390%	-	Food packaging applications.	The plasticizers used are food packaging approved.
AGM	- 0% - 10% - 20% - 30%		- 64.0 - 52.1 - 27.1 - 17.9	- 2840 - 2240 - 35.0 - 107.0	- 3.0% - 32.0% - 335.0% - 320.0%	-		
DBS	- 0% - 10% - 20% - 30%		- 64.0 - 39.2 - 23.1 - 18.3	- 2840 - 2000 - 430.0 - 370.0	- 3.0% - 2.30% - 269.0% - 333.0%	-		
PPG	- 0% - PPG 425 (5.0%) - PPG 425(7.5%) - PPG 425 (10.0%) - PPG 425 (12.5%) - PPG 1000 (5.0%) - PPG 1000 (7.5%) - PPG 1000 (10.0%) - PPG 1000 (12.50%)	PLA [63].	- 25.5 - 20.7 - 17.7 - 21.0 - 21.0 - 22.2 - 22.6 - 22.8 - 21.6	-	- 64% - 19.0% - 107% - 524% - 702% - 44% - 329% - 473% - 496%	-	-	PPGs increased the ability of the used PLA to plastically deform in a more efficient way than PEG.
PEO	- 0% - 5% - 10% - 15% - 20%	PLLA [64].	- 58 - 54.5 - 54 - 35 - 24	-	- 7% - 7% - 11% - 50% - >500%	-	Nerve guides, barriers to tissue adhesion and orbital floor reconstruction.	The initial degradation of PLLA/PEO was more rapid than the neat PLLA and degradation rate increased with increasing the PEO content.
Triethyl citrate ^c^	- 0% - 10% - 20% - 30%	PLA, in the form of films [65].	- 51.7 - 28.1 - 12.6 - 7.2	-	- 7% - 21.3% - 382% - 610%	-	-	Citrates with low molecular weight has increased the rate of eczematic degradation while the degradation rate has decreased when high molecular weight citrates were used.
Tributyl citrate ^c^	- 0% - 10% - 20%		- 51.7 - 22.4 - 7.1	-	- 7% - 6.2% - 350%	-	-	
Acetyl triethyl citrate ^c^	- 0% - 10% - 20% - 30%		- 51.7 - 34.5 - 9.6 - 7.6	-	- 7% - 10% - 320% - 228%	-	-	
Acetyl tributyl citrate ^c^	- 0% - 10% - 20%		- 51.7 - 17.7 - 9.2	-	- 7% - 2.3% - 420%	-	-	
EVA	- 0% - 10% - 30% - 50% - 70% - 90%	PLLA [66].	- 55.89 - 45.11 - 32.36 - 16.67 - 16.67 - 13.73	- 2853.73 - 1804.42 - 1314.09 - 1274.86 - 1284.67 - 627.62	- 4.5% - 4.7% - 6.9% - 10.2% - 9.0% - 208.9%	-	-	-
Limonene	- 0% - 20% - 20% with 1% L101 as a free radical initiator.	PLA [68].	- 60.60 - 15.80 - 17.20	- 2300 - 1000 - 1200	- 7.40% - 117.50% - 120.20%	- 2.70 - 5.50 - 5.80	Transparent packaging applications.	Biobased plasticizers
Myrcene	- 20% - 20% with 1% L101.		- 18.70 - 24.80	- 1900 - 1700	- 62.70% - 45.00%	- 12.50 - 4.90	Opaque packaging applications.	
Ozonized soybean oil	- 0% - 5% - 10% - 15%	PLA [69].	- 54.00 - 46.00 - 41.00 - 34.00	- 1500 - 1520 - 1400 - 1450	- 5.50% - 6.50% - 10.50% - 8.50%	- 2.00 - 2.10 - 2.20 - 2.50	Applications requiring flexibility and toughness.	Biobased plasticizer
ECO	- 0% - 2.50% - 5.00% - 7.50% - 10.00%	PLA [70].	- 44.00 - 42.00 - 39.00 - 36.00 - 34.00	- 3120 - 3050 - 3070 - 2950 - 2930	- 8.50% - 17.00% - 33.50% - 58.00% - 64.00%	-	Packaging applications.	Biobased plasticizer
DBM	- 0% - 7.00% - 12.00%	PLA [71].	- 19.00 - 45.00 - 15.00	- 1672 - 2245 - 1533	- 1.30% - 2.80% - 3.00%	-	Green alternatives for the production of PLAbased flexible films.	Biodegradable plasticizers.
DBF	- 7.00% - 12.00%		- 30.00 - 10.00	- 584 - 279	- 111.90% - 210.00%	-		

^a^ NP: not provided. ^b^ All the impact samples used in this study were un-notched. ^c^ Molecular weights for Triethyl citrate, Tributyl citrate, Acetyl triethyl citrate and Acetyl tributyl citrate are 276 g/mol, 360 g/mol, 318 g/mol and 402 g/mol, respectively. Note: Studies in which no exact values for the mechanical properties were given, the best estimations were provided. Abbreviations: PLA, poly(lactic acid); PEG, poly(ethylene-glycol); m-PEG, PEG monolaurate; ATBC, acetyl tri-n-butyl citrate; PBOH, poly(1,3-butanediol); AGM, acetyl glycerol monolaurate; DBS, dibutyl sebacate; PPG, poly(propylene glycol); PEO, poly(ethylene oxide); PLLA, poly(L-lactic acid); EVA, poly(ethylene-*co*-vinyl acetate); L101, luperox 101; ECO, epoxidized chia seed oil; DBM, dibutylmaleate; DBF, dibutylfumarate.

**Table 6:** Various PLA’s impact modifiers along with their features and applications.

Impact Modifier and Reference/s	Company	Application	Features	Comments
-Sukano^®^ PLA im S550 [80].	-Sukano Co.	-Transparent applications such as packaging.	-Highly cost effective. -At a 4% concentration, the impact resistance of PLA can be enhanced by a factor of 10.	-Compostable and can be used with FDA approved, biodegradable PLA.
-OnCap™BIO Impact T [80,81].	-PolyOne.	-Transparent applications such as packaging.	-Improves the impact resistance of PLA while maintaining its transparency. -Improves tear resistance.	-Designed to improve the applicability of biodegradable and bio-derived polymers. -If used at prescribed loadings, it does not limit the biodegradability or food contact use of the PLA compound.
-Biomax^®^ Strong 100 and 120 [80,82,83].	-DuPont Co.	-Packaging including food packaging and industrial applications.	-Enhance PLA’s toughness and impact strength with minimal effect on transparency. -At a 2% concentration, the impact resistance of PLA can be substantially enhanced.	-Biomax^®^ Strong 100 is designed for non-food applications, while Biomax^®^ Strong 120 is designed for food packaging applications.
-Paraloid™ BPM-500, 515 and 520 [80,84].	-Dow Chem. Co.	-Packaging, electronics, medical, injection molding and automobile applications.	-Improve the mechanical properties of PLA while maintaining its transparency. -Improvement in flexibility, slitting and cutting. -The addition of only 3% concertation can lead to an improvement in the impact Properties of PLA. -Paraloid™ BPM-520 significantly enhances the toughness of PLA and PLA blends with minimal effect on stiffness and heat distortion temperature. Moreover, it features excellent room temperature impact performance. It also provides excellent surface finish and exceptional combinations of color ability and impact strength in opaque PLA and PLA blends applications.	-Paraloid™ BPM-515 is more efficient and FDA approved for up to 5% in food contact resins.
-Biostrength™ 130, 150, 280 and 200 [80].	-Arkema.	-Packaging, injection molding, transparent and opaque applications.	-Biostrength™ 130 and 200 are intended to improve PLA’s toughness while maintaining its transparency. -Biostrength™ 150 is used in opaque and durable injection molding applications. -Biostrength™ 280 is utilized in applications that require high transparency and toughness.	-

Abbreviations: PLA, poly (lactic acid); FDA, Food and Drug Administration.

**Table 7:** The effect of various PLA/PHAs blends at different concentrations on the mechanical properties along with their applications.

Blend	Concentration (wt.%)	Tensile Strength (MPa)	Young’s Modulus (MPa)	Percentage Elongation	Charpy Break Energy/ (Notched Izod Break Energy)	Toughness	Application and/or Reference
PLLA/PHBV	- 100/0 - 80/20 - 60/40 - 40/60 - 20/80	- 71.00 - 54.00 - 39.00 - 29.00 - 24.00	- 2415 - 2083 - 1552 - 1258 - 1076	- 5.60% - 6.20% - 6.70% - 4.10% - 6.90%	-	-	Biomedical applications [91].
	- 100/0 - 80/20 - 60/40 - 40/60 - 20/80	- 29.70 - 27.80 - 22.20 - 25.10 - 24.90	- 2031 - 1761 - 1580 - 1301 - 1631	-	-	-	Biomedical applications (surgical implants, sutures and drug delivery) [92].
	- 100/0 - 0/100 - 50/50 - 40/60 - 30/70	- 62.00 - 22.00 - 39.00 - 38.00 - 33.00	- 2700 - 900 - 1800 - 1700 - 1300	- 8.00% - 13.00% - 7.90% - 7.70% - 7.60%	- (29.00) J/m - (49.00) J/m - (28.00) J/m - (27.00) J/m - (27.00) J/m	-	[98]
	- 100/0 - 100/0 with 5% Lapol as a plasticizer - 100/0 with 7% Lapol - 75/25 - 75/25 with 5% Lapol - 75/25 with 7% Lapol	- 42.00 - 14.00 - 16.00 - 16.00 - 13.00 - 15.00	- 1400 - 1450 - 1200 - 1270 - 1150 - 1120	- 7.20% - 14.40% - 13.70% - 7.10% - 15.50% - 15.10%	-	-	Single use applications such as food packaging [108].
	- 50/50 - 50/50 with 2% PLLA-PEG-PLLA triblock - 50/50 with 5% PLLA-PEG-PLLA triblock - 50/50 with 2% PEG-PLLA diblock - 50/50 with 5% PEG-PLLA diblock - 50/50 with 2% PVAc - 50/50 with 5% PVAc	- 49.60 - 69.80 - 38.50 - 65.50 - 32.70 - 41.50 - 43.40	- 2737 - 2291 - 1921 - 2581 - 2155 - 1850 - 2087	- 4.40% - 5.10% - 5.10% - 4.40% - 5.90% - 4.80% - 4.90%	-	- 5.90 N.mm - 9.20 N.mm - 7.90 N.mm - 6.50 N.mm - 8.30 N.mm - 8.40 N.mm - 6.60 N.mm	[93]
PLA/PHA	- 100/0 - 90/10 - 80/20 - 70/30	- 55.00 - 50.00 - 37.00 - 35.00	-	-	- 0.052 J - 0.081 J - 0.137 J - 0.161 J	-	Biodegradable blends for applications that require improved impact toughness (impact toughness similar to that of PS and ABS) [94].
PLA/ePHA	- 90/10 - 80/20 - 70/30	- 53.00 - 48.00 - 37.00	-	-	- 0.089 J - 0.169 J - 0.260 J	-	
PLA/Nodax™	- 100/0 - 90/10 - 80/20 - 60/40 - 40/60	-	-	-	-	- 0.30 N.m - 1.90 N.m - 1.40 N.m - 0.30 N.m - 0.20 N.m	Ductile and tough plastics applications [95].
	- 100/0 - 90/10 - 85/15 - 80/20 - 75/25 - 81/14 with 5% *oligo*Nodax™-*b*-PLLA diblock copolymer (81/14/5 wt.%)	-	-	-	- (22.00) J/m - (27.00) J/m - (44.00) J/m - (43.00) J/m - (35.00) J/m - (44.00) J/m	-	Ductile and tough plastics applications [96].
PLA/PHB	- 100/0 - 25/75 - 50/50 - 75/25	- 26.00 - 2.50 - 8.00 - 32.50	-	- 16.00% - 6.00% - 11.00% - 17.50%	-	-	Applications that require high biodegradation rate [43].
	- 100/0 - 83/17 - 57/43 - 50/50 - 43/57 - 29/71 - 17/83 - 0/100	- 50.00 - 47.00 - 40.00 - 37.50 - 35.50 - 34.50 - 27.00 - 26.50 - 26.00		- 7.25% - 3.50% - 3.85% - 3.40% - 3.20% - 3.20% - 3.00% - 2.70% - 4.35%	-	-	Environmentally friendly packaging [110].
	- 100/0 - 75/25 - 63.75/21.25 with 15% ATBC - 60/20 with 5% CNCs and 15% ATBC - 60/20 with 5% CNCs-m and 15% ATBC	- 46.90 - 38.20 - 40.20 - 27.30 - 28.20	- 1240 - 1810 - 550 - 570 - 490	- 41.10% - 13.00% - 90.10% - 27.40% 147.70%	-	-	Biodegradable packaging [103].
PLA/aPHB	- 100/0 - 98/2 - 95/5 - 90/10 - 85/15 - 80/20	- 49.30 - NP ^a^ - 46.00 - 43.50 - 38.30 - 30.50	- 3500 - NP - 3380 - 3240 - 2910 - 2750	- 6.00% - NP - 6.00% - 7.00% - 9.00% - 21.00%	- 50.00 ^b^ KJ/m^2^ - 60.00 ^b^ KJ/m^2^ - 60.00 ^b^ KJ/m^2^ - 61.00 ^b^ KJ/m^2^ - 103.00 ^b^ KJ/m^2^ - 118.00 ^b^ KJ/m^2^	-	Packaging, especially for food [97].
PLA/PHBHH_x_	- 100/0 - 80/20 - 60/40 - 50/50 - 40/60 - 20/80 - 0/100	- 36.40 - 29.50 - 33.50 - 22.10 - 27.70 - 23.60 - 17.60	- 1390 - 1320 - 1240 - 910 - 1250 - 590 - 370	- 13.80% - 99.60% - 7.68% - 7.26% - 11.50% - 83.50% - 19.30%	-	-	Biomedical applications, such as artificial vascular graft [100].
	- 100/0 - 90/10 - 80/20 - 60/40 - 0/100	- 62.20 - 54.10 - 45.30 - 40.10 - 21.60	- 1603.00 - 1416.00 - 1265.00 - 1093.00 - 309.00	- 3.60% - 7.60% - 113.10% - 37.60% - 524.80%	-	- 3.20 MPa - 4.00 MPa - 68.70 MPa - 20.40 MPa - 160.60 MPa	Food packaging and flexible films [102].

^a^ NP: not provided. ^b^ Impact test was done as per ISO 8256 standard (method A). Note: Studies in which no exact values for the mechanical properties were given, the best estimations were provided. Abbreviations: PLLA, poly(L-lactic acid); PHBV, poly(3-hydroxybutyrate-*co*-3-hydroxyvalerate); PEG, polyethylene glycol; PVAc, poly (vinyl acetate); PLA, poly(lactic acid); PHA, poly(3-hydoroxyalkanoate); ePHA, epoxidized PHA; Nodax™, [poly (3-hydroxybutyrate-*co*-3-hydroxyhexanoate)]; PHB, poly[^®^-3-hydroxybutyrate]; ATBC, acetyl (tributyl citrate); CNCs, cellulose nanocrystals; CNCs-m, modified cellulose nanocrystals; a-PHB, atactic PHB; PHBHH_x_, poly(3-hydroxybutyrate-*co*-3-hydroxyhexanoate).

**Table 8:** The effect of various PLA/PCL blends at different concentrations on the mechanical properties along with their applications.

Blend	Concentration (wt.%)	Tensile Strength (MPa)	Young’s Modulus (MPa)	Percentage Elongation	Charpy Impact/ (Izod Impact)	Application and/or Reference
PLA/PCL	- 100/0 - 80/20 - 60/40 - 40/60 - 20/80 - 80/20 with 2% TPP as a coupling agent - 60/40 with 2% TPP - 40/60 with 2% TPP - 20/80 with 2% TPP	- 48.26 - 44.19 - 19.37 - 18.61 - 20.13 - 33.09 - 23.58 - 11.44 - 17.23	- 2275.26 - 584.67 - 751.52 - 164.09 - 111.69 - 1013.52 - 710.16 - 344.04 - 197.87	- 3.0% - 28.0% - 5.00% - 23.0% - 440% - 127% - 7.00% - 3.00% - 560%	-	[77]
	- 100/0 - 70/30 - 70/30 with 0.1 phr ^a^ Dicumyl peroxide - 70/30 with 0.2 phr Dicumyl peroxide - 70/30 with 0.3 phr Dicumyl peroxide	- 70 - 55 - 52 - 49 - 48	- 1500 - 1300 - 1200 - 1150 - 1100	- 11.0% - 20% - 35% - 160% - 149%	- (2.00) KJ/m^2^ - (3.80) KJ/m^2^ - (3.90) KJ/m^2^ - (4.00) KJ/m^2^ - (4.90) KJ/m^2^	High performance applications [115].
	- 100/0 - 95/5.0 ^b^ - 95/5.0 ^c^ - 95/5.0 ^d^	- 45.13 - 58.62 - 52.21 - 44.49	- 3729 - 3631 - 3422 - 3661	- 2.06% - 3.12% - 2.90% - 2.32%	-	[119]
	- 0/100 - 20/80 - 30/70 - 40/60 - 60/40 - 70/30 - 80/20 - 100/0	-	- 460 - 700 - 1220 - 1350 - 2030 - 2500 - 2940 - 3910	-	-	Biomedical applications [120].
	- 0/100 - 0/100 with 1% PDI as a compatibilizer - 90/10, linear PCL - 90/10, linear PCL with 1% PDI - 90/10, three-armed star shaped PCL - 90/10, three-armed star shaped PCL with 1% PDI - 90/10, four-armed star shaped PCL - 90/10, four-armed star shaped PCL with 1% PDI - 90/10, six-armed star shaped PCL - 90/10, six-armed star shaped PCL with 1% PDI	-	- 4600 - 4200 - 3400 - 3600 - 2100 - 2400 - 2250 - 2800 - 3450 - 3500	- 4.00% - 2.30% - 6.40% - 6.43% - 8.20% - 8.00% - 8.40% - 8.37% - 8.60% - 8.65%	- (8.00) KJ/m^2^ - (11.50) KJ/m^2^ - (20.00) KJ/m^2^ - (17.00) KJ/m^2^ - (20.50) KJ/m^2^ - (17.50) KJ/m^2^ - (23.00) KJ/m^2^ - (21.00) KJ/m^2^ - (24.50) KJ/m^2^ - (21.00) KJ/m^2^	Different daily and industrial applications such as, disposable products, biomedical products and food packaging [121].
	- 100/0 - 80/20 - 70/30 - 60/40	- 66.05 - 53.60 - 50.20 - 41.30	- 1311 - 1233 - 1223 - 884	- 8.21% - 476.70% - 514.60% - 664.70%	-	Applications requiring very high toughness properties [123].
PLLA/PCL	- 100/0 - 80/20 - 80/20 with 10% poly(L-lactide-*co*-ε-caprolactone)	- 60 - 30 - 40	- 1300 - 1100 - 1100	- 5.0% - 175.0% - 300%	-	[114]
	- 100/0 - 80/20 - 64/16 with 20% poly(e-caprolactone/L-lactide)	- 35 - 31.0 - 11.0	- 2530 - 2080 - 660	- 1.6% - 9.60% - >100%	- 41.4 KJ/m^2^ at (−)20 °C and 49.2 KJ/m^2^ at 23 °C. -NP ^e^ - 5.3 KJ/m^2^ at (−)20 °C and 10.1 KJ/m^2^ at 23 °C.	Biomedical applications [111]
	- 70/30 - 70/30 with 4% triblock PLLA -PCL-PLLA as compatibilizing agent	-	- 1400 - 1400	- 2.00% - 53.0%	- 1.1 KJ/m^2^ - 3.7 KJ/m^2^	Biomedical applications [113].
	- 100/0 - 80/20 - 60/40 - 50/50 - 80/20 with copolymer of ethylene oxide and propylene oxide surfactant - 60/40 with copolymer of ethylene oxide and propylene oxide surfactant - 50/50 with copolymer of ethylene oxide and propylene oxide surfactant	- 34.10 - 41.20 - 19.30 - 16.90 - 20.10 - 12.90 - 10.40	- 19.8 - 20.7 - 10.7 - 8.10 9.50 - 4.70 - 6.60	- 56.30% - 129.50% - 152.10% - 139.60% - 129.00% - 130.00% - 123.70%	-	Orthopedic and dental applications [118].
Triblock copolymer of PLA (85% L-lactide and 15% D-lactide), ε-CL and TMC	- 100/0 - 80/20	- 56.8 - 36.0	-	-	- (41.0) J/m - (293–520) J/m	Load bearing devices in biomedical applications [116].

^a^ phr: per hundred of rubber. ^b^ PCL’s molecular weight = 10,000 g/mol. ^c^ PCL’s molecular weight = 40,000 g/mol. ^d^ PCL’s molecular weight = 70,000–10,0000 g/mol. ^e^ NP: not provided. Note: Studies in which no exact values for the mechanical properties were given, the best estimations were provided. Abbreviations: PLA, poly(lactic acid); PCL, poly(ε-caprolactone); TPP, triphenyl phosphite; PDI, 1,4-phenylene diisocyanate; PLLA, poly(L-lactic acid); ε-CL, ε-caprolactone; TMC, trimethylene carbonate.

**Table 9:** The effect of various PLA/other degradable or partial degradable polymers blends at different concentrations on the mechanical properties along with their applications.

Blend	Concentration (wt.%)	Tensile Strength (MPa)	Yield Strength (MPa)	Young’s Modulus (MPa)	Percentage Elongation	Break Energy/ (Izod Break Energy)	Application and/or Reference
PLA/PPD	- 100/0 - 80/20 - 50/50 - 20/80	- 25.30 - 15.60 - 5.30 - 5.00	-	- 1400 - 1550 - 900 - 650	- 14.5% - 55.0% - 3.00% - 4.00%	NP ^a^	Medical applications [124].
PLA/PPC	- 100/0 - 85/15 - 70/30 - 60/40 - 50/50 - 40/60 - 30/70 - 15/85	- 59.0 - 45.0 - 42.0 - 28.0 - 26.0 - 25.0 - 21.0 - 14.0	- 59.0 - 49.0 - 46.0 - 41.0 - 37.0 - 36.0 - 32.0 - 24.0	- 3150 - 2450 - 2150 - 2050 - 1750 - 1400 - 1050 - 800	-	- 2.00 ^b^ J/cm^2^ - 5.00 ^b^ J/cm^2^ - 14.00 ^b^ J/cm^2^ - 55.00 ^b^ J/cm^2^ - 84.00 ^b^ J/cm^2^ - 75.00 ^b^ J/cm^2^ - 72.00 ^b^ J/cm^2^ - 69.00 ^b^ J/cm^2^	[125]
PLA/PBAT	- 100/0 - 95/5 - 90/10 - 85/15 - 80/20	- 63.00 - 58.00 - 55.00 - 51.00 - 47.00	-	- 3200 - 2900 - 2850 - 2700 - 2600	-	- (2.70) KJ/m^2^ - (2.75) KJ/m^2^ - (2.90) KJ/m^2^ - (3.50) KJ/m^2^ - (4.40) KJ/m^2^	Applications requiring increased toughness while maintaining degradability [127].
PLA/Bionolle (B1001) ^c^	- 100/0 - 95/5 - 90/10 - 80/20 - 70/30 - 60/40 - 50/50	- 36.00 - 32.00 - 36.00 - 24.00 - 28.00 - 26.00 - 24.00	-	- 2481 - 2471 - 2158 - 1766 - 1704 - 1468 - 1268	- 2.00% - 1.70% - 2.40% - 5.00% - 4.00% - 5.00% - 4.20%	-	Biomedical and food applications [128].
PLA/Bionolle (B3010) ^c^	- 95/5 - 90/10 - 80/20 - 70/30 - 60/40 - 50/50	- 27.00 - 31.00 - 26.00 - 24.00 - 22.00 - 19.00	-	- 2389 - 2292 - 1836 - 1620 - 1359 - 1071	- 1.50% - 1.80% - 2.20% - 2.40% - 8.20% - 3.30%	-	
PLLA/PTAT	- 100/0 - 75/25 - 50/50 - 25/75	- 28.12 - 24.62 - 7.11 - 11.11	-	-	- 19.33% - 97.00% - 34.00% - 285.33%	-	Medical applications, tissue engineering and drug delivery [126].
PLLA/PBSL	- 100/0 - 99/1 - 95/5 - 90/10 - 80/20 - 99/1 with 10% RKM as a plasticizer - 95/5 with 10% RKM - 90/10 with 10% RKM - 80/20 with 10% RKM - 99/1 with 20% RKM - 95/5 with 20% RKM - 90/10 with 20% RKM - 80/20 with 20% RKM	- 63.00 - 61.00 - 62.00 - 55.00 - 51.50 - 35.00 - 29.00 - 28.00 - 30.00 - 22.00 - 21.00 - 21.00 - 21.00	-	- 2900 - 2800 - 2650 - 2450 - 2350 - 1900 - 1150 - 1000 - 1250 - 600 - 650 - 700 - 700	- 2.00% - 3.00% - 55.00% - 160% - 120% - 220% - 245% - 240% - 235% - 195% - 200% - 195% - 170%	-	Packaging applications [132].
PLLA/PBSA	- 75/25	-	- 36.70	- 1160.90	- 153.60%	-	Biodegradable sealing envelope for food packaging [131].
PLLA/PBS	- 100/0 - 0/100 - 75/25	-	- 64.60 - 32.10 - 44.70	- 2214.70 - 326.30 - 1075.20	- 6.90% - 320.60% - 71.80%	-	[130]

^a^ NP: not provided. ^b^ The type of impact test was not provided. ^c^ Bionolle (B1001) and Bionolle (B3010) are different in the amount of Bionolle. Note: Studies in which no exact values for the mechanical properties were given, the best estimations were provided. Abbreviations: PLA, poly(lactic acid); PPD, poly-*p*-dioxanone; PPC, poly(propylene carbonate); PBAT, poly(butylene adipate-*co*-terephthalate); Bionolle, poly(ethylene/butylene succinate); PLLA, poly(L-lactic acid); PTAT, poly(tetramethylene adipate-*co*-terephthalate); PBSL, poly(butylene succinate-*co*-L-lactate); RKM, Rikemal PL710; PBSA, poly(butylene succinate-*co*-butylene adipate); PBS, poly(butylene succinate).

**Table 10:** Features of selected studies on PLA blends along with their applications.

Blend	Concentration (wt.%)	Features	Applications and/or Reference
PLA/PHB	- 80/20 and 60/40	-Improvement in the percentage elongation at break.	-Biomedical applications [133].
	- 75/25	-Higher elongation at break with the use of 5% Lapol	-Single-use applications such as fast-food packaging [108].
	-	-Improved biodegradation rate, flexibility and impact properties.	-Food packaging [134].
	- 75/25	-Improved barrier and mechanical properties.	-Food packaging [107].
	- 75/25	-Biodegradable blend.	-Biodegradable food packaging [105].
	- 85/15	-Good barrier to water vapor and improved oxygen barrier properties	-Active food packaging [135].
	-	-Enhanced mechanical and active properties	-Biodegradable active packaging for chilled salmon [142].
PLA/PHBV	- 75/25 and 50/50	-Improved permeability.	-Food packaging [139].
PLA/PBS	- 90/10, 80/20 and 70/30	-Exceptional combination of ductility, modulus and strength.	-Green packaging [136].
	- 90/10, 80/20 and 70/30	-Enhancement in PLA’s water vapor and oxygen permeability. -The levels of migration were maintained below the European legislative limits.	-Biodegradable food packaging [140].
	- 80/20	-Improved elongation at break.	-Food packaging [137].
	- 90/10	-Higher antibacterial activity. -Transparent sheets. -Mechanical properties allowed thermoforming for applications of food packaging.	-Antibacterial food packaging sheets [143].
PLA/PCL	- 90/10, 85/15, 80/20, 75/25, 70/30, 60/40 and 50/50	-Well balanced combination of toughness and stiffness.	-Packaging, biomedical and agricultural applications [138].
PLA/PBAT	- 70/30	-Migration levels were below the limit specified by Food contact materials EU NO. 10/2011; therefore, the blend is safe for food contact packaging applications.	-Food contact materials for containers and packaging [141].
PLA/PHBV/PBS	- 60/30/10 and 60/10/30	-Entirely biodegradable. -An enhancement in the PLA’s crystallization, flexibility and toughness was observed in the resulting ternary complex. -Optimum performance with excellent balanced thermal resistance and stiffness-toughness.	[144]

Abbreviations: PLA, poly(lactic acid); PHB, polyhydroxybutyrate; PHBV, poly(3-hydroxybutyrate-*co*-3-hydroxyvalerate); PBS, poly(butylenes succinate); PCL, poly(ε-caprolactone); PBAT, poly(butylene adipate terephthalate).

**Table 11:** The effect of various PLA nanocomposites at different concentrations on the mechanical properties along with their applications.

Composite/ Nano- Composite	Concent- Ration (wt.%)	Tensile Strength (MPa)	Young’s Modulus (MPa)	Elongation at Break	Charpy Break Energy/ (Notched Izod Break Energy)/ *Toughness*	Flexural Strength (MPa)	Flexural Modulus (MPa)	Applications and/or Reference
PLA/MMT	- 100/0 - 100/2 phr ^a^ - 100/4 phr - 90/2 phr with 10% LLDPE - 90/4 phr with 10% LLDPE	- 58.0 - 55.0 - 53.0 - 41.50 - 42.50	- 3000 - 3400 - 3500 - 2650 - 2850	-	-	- 109 - 84 - 83 - 75 - 66	- 3300 - 3500 - 3850 - 3000 - 3150	Structural applications [145].
PLA/talc	- 100/0 - 98/2 - 95.5/4.5 - 90.9/9.1 - 87.2/12.8 - 81.9/18.1 - 75.7/24.3	- 54.0 - 58.5 - 58.4 - 58.5 - 58.2 - 59 - 60	-	- 2.4% - 2.5% - 2.65% - 3.20% - 4.1% - 5.1% - 2.4%	-	- 66.0 - 90.5 - 91.0 - 93.0 - 94.0 - 98.0 - 103.5	- 3300 - 3450 - 4000 - 4300 - 4900 - 6100 - 6750	Packaging applications [149].
	- 100/0 - 95/5 - 90/10 - 80/20 - 70/30	- 47.0 - 47.0 - 48.0 - 46.0 - 48.0	- 2400 - 2560 - 3050 - 3650 - 4550	- 6.70% - 3.0% - 3.0% - 2.50% - 1.70%	-	-	-	Packaging applications [150].
PLA/kaolinite	- 100/0 - 95/5 - 90/10 - 80/20 - 70/30	- 47.0 - 48.0 - 42.0 - 42.0 - 46.0	- 2400 - 2550 - 2700 - 3100 - 3350	- 6.70% - 2.4% - 1.90% - 1.50% - 1.40%	-	-	-	Packaging applications [150].
PLA/CNT	- 100/0 ^b^ - 99/0.1 ^b^ - 99.5/0.5 ^b^ - 99/1 ^b^ - 98/2 ^b^	- 39.50 - 40.50 - 42.80 - 40.60 - 39.60	-	- 22.50% - 28.30% - 33.60% - 26.70% - 20.50%	- (15.50) KJ/m^2^ - (22.60) KJ/m^2^ - (27.70) KJ/m^2^ - (20.50 KJ/m^2^) - (8.80) KJ/m^2^	-	-	Industrial applications [151].
	- 100/0 - 99/1 - 97/3 - 95/5 - 90/10	- 60.5 ^c^ - 65.0 ^c^ - 68.0 ^c^ - 65.0 ^c^ - 63.0 ^c^ - 51.0 ^d^ - 58.0 ^d^ - 67.5 ^d^ - 64.5 ^d^ - 59.5 ^d^	-	- 5.00% ^c^ - 5.60% ^c^ - 5.70% ^c^ - 5.70% ^c^ - 5.90% ^c^ - 5.00% ^d^ - 5.00% ^d^ - 7.00% ^d^ - 6.00% ^d^ - 4.80% ^d^	-	-	-	Applications requiring good electrical and mechanical properties [152].
	- 100/0 - 80/0 with 20% PHBV - 80/0.5 with 20% PHBV - 80/1 with 20% PHBV	-	-	-	- (2.14) KJ/m^2^ - (4.10) KJ/m^2^ - (2.33) KJ/m^2^ - (2.46) KJ/m^2^	- 58.07 - 51.60 - 58.66 - 61.01	- 2940 - 3100 - 3300 - 3250	Electronic devices and military applications [153].
PLA/KF	- 100/0 - 89/10 ^e^ with 1% MWCNTs - 79/20 ^e^ with 1% MWCNTs - 69/30 ^e^ with 1% MWCNTs - 59/40 ^e^ with 1% MWCNTs - 100/0 ^f^ - 89/10 ^f^ with 1% MWCNTs - 79/20 ^f^ with 1% MWCNTs - 69/30 ^f^ with 1% MWCNTs - 59/40 ^f^ with 1% MWCNTs - 70/30 ^g^	- 49.70 - 61.60 - 62.80 - 78.50 - 47.70 - 46.80 - 61.60 - 70.40 - 91.50 - 53.60	-	-	- (17.50) J/m - (30.80) J/m - (36.80) J/m - (37.40) J/m - (43.80) J/m - (30.90) J/m - (30.30) J/m - (35.50) J/m - (35.40) J/m - (44.90) J/m	-	-	Antistatic applications [154].
	- 100/0 - 100/0 ^f^ - PLA/20 pph ^h^ - PLA/20 pph ^f^	- 63.20 - 65.30 - 39.50 - 32.70	- 1410 - 1634 - 1618 - 1742	- 6.60% - 5.30% - 3.50% - 2.50%	-	-	-	Packaging applications such as hot boiling water containers [157].
	- 90/10 - 70/30 - 50/50 - 30/70 - 90/10 with 1% GPS as a coupling agent - 70/30 with 1% GPS - 50/50 with 1% GPS - 30/70 with 1% GPS - 90/10 with 3% GPS - 70/30 with 3% GPS - 50/50 with 3% GPS - 30/70 with 3% GPS - 90/10 with 5% GPS - 70/30 with 5% GPS - 50/50 with 5% GPS - 30/70 with 5% GPS	-	-	-	-	- 22.0 - 40.0 - 49.0 - 50.0 - 38.0 - 43.0 - 60.0 - 63.0 - 30.0 - 49.0 - 64.0 - 62.0 - 37.0 - 48.0 - 63.0 - 62.0	- 2100 - 4000 - 4700 - 5900 - 4700 - 4000 - 5700 - 6800 - 4200 - 4500 - 5800 - 6800 - 4200 - 4650 - 5700 - 6800	Prototypes of automobile headliners [162].
PLA/nano clay or organoclay	- 100/0 - 100/0 ^f^ - PLA with 5 pph Cloisite 30B^®^ - PLA with 5 pph Cloisite 30B^® f^	- 63.20 - 65.30 - 51.20 - 51.60	- 1410 - 1634 - 1599 - 1893	- 6.60% - 5.30% - 5.20% - 3.50%	-	-	-	Packaging applications such as hot boiling water containers [157].
	- PLA (100/0) -PLA/PCL ^i^/Organoclay 9S-Ben W (90.48/4.76/4.76 wt.%) -PLA/PCL ^j^/Organoclay 9S-Ben W (90.48/4.76/4.76 wt.%) -PLA/PCL ^k^/Organoclay 9S-Ben W (90.48/4.76/4.76 wt.%)	- 45.13 - 47.26 - 53.91 - 39.94	- 3729 - 4371 - 4069 - 4237	- 2.06% - 2.24% - 3.18% - 2.00%	-	-	-	[119]
	- PLLA/PBS (100/0) - PLLA/PBS (0/100) - PLLA/PBS (75/25) - PLLA/PBS 75/25 with 2% Cloisite 25 A^®^ - PLLA/PBS 75/25 with 5% Cloisite 25 A^®^ -PLLA/PBS 75/25 with 10% Cloisite 25 A^®^ - PLLA/PBS 75/25 with 2% TFC - PLLA/PBS 75/25 with 5% TFC -PLLA/PBS 75/25 with 10% TFC	-	- 2214.70 - 326.30 - 1075.20 - 1364.60 - 1616.60 - 1940.10 - 1407.90 - 1624.60 - 1990.30	- 6.90% - 320.60% - 71.80% - 4.40% - 4.10% - 3.60% - 75.50% - 100.60% - 118.10%	-	-	-	[130]
	- PLLA/PBSA 75/25 with 2% Cloisite 25 A^®^ - PLLA/PBSA 75/25 with 5% Cloisite 25 A^®^ - PLLA/PBSA 75/25 with 10% Cloisite 25 A^®^ - PLLA/PBSA 75/25 with 2% TFC - PLLA/PBSA 75/25 with 5% TFC - PLLA/PBSA 75/25 with 10% TFC	-	- 1394.10 - 1585.00 - 1748.40 - 1445.60 - 1698.30 - 1780.70	- 11.30% - 10.60% - 5.25% - 69.50% - 43.10% - 45.70%	-	-	-	Biodegradable sealing envelope for food packaging [131].
3D-printed PLA wastes/SiO_2_	- 100/0 - 95/5 - 90/10 - 85/15	- 62.80 - 76.50 - 121.00 - 53.90	- 839.60 - 895.10 - 1020.70 - 793.20	- 11.10% - 12.60% - 15.30% - 11.40%	- *3.60 MPa* - *4.60 MPa* - *5.60 MPa* - *3.10 MPa*	-	-	Recycled PLA filaments for 3D printing [158].
PLA/MgO	- 100/0 - 99/1 - 98/2 - 97/3 - 96/4	- 29.10 - 34.00 - 37.50 - 26.60 - 26.20	- 1891 - 2418 - 2470 - 2101 - 1961	- 4.40% - 3.30% - 3.90% - 2.30% - 2.40%	-	-	-	Food packaging applications that are transparent and require superior antibacterial efficiency [159].
PLA/flax fibers	- 100/0 - PLA/modified non- cellulose oxidizedTiO_2_ grafted flax fibers -PLA/modified cellulose oxidized TiO_2_ grafted flax fibers	- NP ^l^ - 172.00 - 211.00	- 11,000 - 9000 - 105,000	- 3.40% - 3.80% - 4.50%	- (5.00) KJ/m^2^ - (16.10) KJ/m^2^ - (15.70) KJ/m^2^	-	-	[161]
PLA/flax fiber braided yarn plain woven fabric	- 100/0 - 82/18 - 100/74 - 100/65	- 47.00 - 65.00 - 73.00 - 80.00	- 820 - 1090 - 1190 - 1310	- 6.50% - 9.00% - 9.00% - 9.45%		-	-	Housing and automobile interiors such as seat back, door trim and telephone stand [164].
PLA/wood flour	- 100/0 - 100/10 phr - 100/20 phr - 100/30 phr - 100/26 phr with 0.52 phr epoxy silane as a coupling agent and EMAGMA/13 as a compatibilizer - 100/26 phr with 0.52 phr epoxy silane as a coupling agent and EMAGMA/26 as a compatibilizer - 100/26 phr with 0.52 phr epoxy silane as a coupling agent and EMAGMA/52 as a compatibilizer (100%/26 phr/0.52 phr)	- 54.90 - 37.40 - 34.00 - 27.60 - 31.30 - 27.40 - 21.00	-	- 2.50% - 2.60% - 1.80% - 1.30% - 6.90% - 11.70% - 24.40%	- (2.30) KJ/m^2^ - (3.00) KJ/m^2^ - (2.60) KJ/m^2^ - (2.40) KJ/m^2^ - (3.40) KJ/m^2^ - (3.80) KJ/m^2^ - (4.10) KJ/m^2^	-	-	Blow molding applications [165].
PLA/wood powder	- 100/0 - 60/10 with 30% PCL - 53.34/20 with 26.66% PCL - 46.66/30 with 23.34% PCL	- 62.00 - 37.00 - 35.00 - 33.00	- 1300 - 890 - 1000 - 1085	- 12.20% - 12.45% - 11.00% - 10.80%	- 30.00 J/mm - 60.00 J/mm - 57.00 J/mm - 43.00 J/mm	-	-	Disposable cups [166].
PLA/cellulose nanocrystals	- 80/0 with 20% PBS - 79.5/0.5 with 20% PBS - 79.25/0.75 with 20% PBS - 79/1 with 20% PBS - 78.5/1.5 with 20% PBS	- 75.6 - 74.6 - 85.1 - 92.6 - 64.6	- 3200 - 3975 - 6925 - 755 - 3275	- 17.50% - 16.35% - 15.25% - 12.90% - 12.45%	-	-	-	Green packaging [169].
PLA/Lignin	- 100/0 - 80/20 - 78/20 with 2% PEG 2000 as a plasticizer - 75/20 with 5% PEG 2000 - 79.5/20 with 0.5% TR451 as a plasticizer - 79/20 with 1% TR451	- 56.00 - 43.50 - 52.00 - 44.50 - 45.50 - 45.00	- 1800 - 2300 - 2150 - 1600 - 1700 - 2150	- 4.20% - 2.90% - 4.00% - 3.90% - 3.70% - 3.20%	-	-	-	3D printing applications [167].
PLA/MCC	- 100/0 - 50/50 - 95/0 with 5% TEC as a plasticizer - 47.5/47.5 with 5% TEC - 90/0 with 10% TEC - 45/45 with 10% TEC - 85/0 with 15% TEC - 42.5/42.5 with 15% TEC	- 59.00 - 42.00 - 49.00 - 36.00 - 46.00 - 28.00 - 26.00 - 13.00	- 1556 - 2517 - 1553 - 2495 - 1366 - 563 - 161 - 22	- 6.00% - 2.00% - 10.00% - 2.00% - 13.00% - 26.00% - 595.00% - 300.00%	-	-	-	Eco friendly food packaging applications [168].
PLA/HNTs	- 100/0 - 50/1 with 50% PCL - 50/3 with 50% PCL - 50/5 with 50% PCL - 50/7 with 50% PCL	- 17.25 - 11.45 - 12.87 - 15.52 - 16.62	- 246.56 - 184.10 - 213.53 - 267.65 - 281.19	- 7.18% - 12.30% - 8.53% - 9.37% - 6.78%	-	-	-	Bone replacements and regeneration applications [171].
PLA/PFs	- 100/0 - NP	- 54.00 - 62.00	- 1100 - 1450	-	- (139) J/m - (92) J/m	- 103 - 103	- 3500 - 5450	Complex geometries in which the uniform distribution of mechanical performance and fibers are vital [172].
PLA/short carbon fibers	- 100/0 ^m^ - NP ^m^	- 47.80 - 70.30	- 3350 - 9210	-	-	55.60 - 105.50	- 2090 - 6940	Applications demanding dimensional stability and higher stiffness [156].

^a^ phr: per hundred of rubber. ^b^ PLA/CNT-COOH nanocomposites with Ethylene-butyl acrylate-glycidyl methacrylate (E-BA-GMA) used as a compatibilizer. ^c^ Testing direction is vertical to the extrusion direction. ^d^ Testing direction is parralel to the extrusion direction. ^e^ Modified kenaf fiber. ^f^ After annealing. ^g^ Un-modified KF. ^h^ pph: part per hundred. ^i^ PCL’s molecular weight = 10,000 g/mol. ^j^ PCL’s molecular weight = 40,000 g/mol. ^k^ PCL’s molecular weight = 70,000–100,000 g/mol. ^l^ NP: not provided. ^m^ Tested samples were printed in the flat printing direction. Note: Studies in which no exact values for the mechanical properties were given, the best estimations were provided. Abbreviations: PLA, poly(lactic acid); MMT, montmorillonite; LLDPE, linear low-density polyethylene; CNT-COOH, carbon nanotubes with carboxyl groups; CNT, carbon nanotubes; PHBV, poly(3-hydroxybutyrate-*co*-3-hydroxyvalerate); KF, kenaf fiber; MWCNTs, modified multi-walled carbon nanotubes; GPS, 3-Glycidoxypropyl trimethoxy silane; PBS, poly(butylenes succinate); TFC, twice functionalized organoclay; PBSA, poly(butylene succinate-*co*-butylene adipate); SiO_2_, silica; MgO, nano-magnesium oxide; TiO_2_, Titanium dioxide; EMAGMA, ethylene-methyl acrylate-glycidyl methacrylate; PCL, poly(ε-caprolactone); PEG, polyethylene glycol; TR451, Struktol; MCC, microcrystalline cellulose; TEC, Triethylcitrate; HNTs, halloysite nanotubes; PFs, pineapple fibers.

**Table 12:** Typical mechanical properties for PHAs along with other commercial polymers [54,176,177,185].

Polymer ^a^	Tensile Modulus (GPa)	Tensile Strength (MPa)	Percentage Elongation at Break (%)
PHB	1.7–3.5	40	3.0–6.0
PHBV	0.7–2.9	30–38	20
PLA	1.2–2.7	28–50	7.0–9.0
PCL	0.4	16.0	120–800
TPS	0.5–1.0 ^b^	2.6	47.0
PET	2.2	56.0	70–100
LDPE	0.2	10–15	300–500
PP	1.7	35–40	150
PS	1.6–3.1	12–50	3.0–4.0
PVC	0.3–2.4	10–60	12–32

^a^ The values for mechanical properties will vary according to different factors such as, polymer crystallinity, molecular weight, orientation, as well as testing conditions. ^b^ At low water content (5.0–7.0 wt.%). Abbreviations: PHB, polyhydroxybutyrate; PHBV, poly(3-hydroxybutyrate-*co*-3-hydroxyvalerate); PLA, poly(lactic acid); PCL, poly(ε-caprolactone); TPS, thermoplastic starch; PET, poly(ethylene terephthalate); LDPE, low-density poly(ethylene); PP, poly(propylene); PS, poly(styrene); PVC, polyvinyl chloride.

**Table 13:** A summary of selected studies for the biodegradation of PHBVs-based/starch composites.

Percentage of Starch	Biodegradation Environment	Thickness (cm)	Days	Percentage of Weight Loss	Reference
30%	Compost	NP ^a^	20	100%	[226]
30%		0.05		100%	[245]
0%		0.05		60%	
50%	Marine	0.05	150	90–100%	[250]
30%				50–90%	
0%				10–20%	
50%	Soil	0.32	125	49%	[249]
30%				25%	
0%				7%	
50%	Activated sludge	0.08	30	100%	[225]
25%				85%	
0%				30%	

^a^ Not provided.

**Table 14:** Mechanical properties of different PHAs blends and nanocomposites at various concentration along with their applications.

Blend/Composite/Nanocomposite	Concentration (wt.%)	Tensile Strength (MPa)	Young’s Modulus (MPa)	Percentage Elongation	Charpy Impact Strength/(Notched Izod Break Energy)	Applications and/or Reference
PHB/PCL	- 100/0 - 75/25 - 50/50 - 25/75	- 22.20 - 21.40 - 19.80 - 17.30	- 1939 - 1643 - 1387 - 690	- 8.10% - 11.20% - 17.60% - >1000%	-	[195]
PHA ^a^/PCL	- 70/30 - 50/50 - 30/70	- 4.0 ^b^ - 5.0 ^b^ - 13.0 ^b^	-	- 4.00% ^b^ - 64.00% ^b^ - 63.00% ^b^	-	Medical applications and packaging [197].
P(3HO-3HD)/PCL	- 100/0 - 75/25 - 95/5	- 14.30 - 5.90 - 13.70	- 8.40 - 110 - 13.70	- 640.00% - 490.00% - 620.00%	-	Nerve re-generation [198]
PHB/PBS	- 100/0 - 80/20 - 80/20 with 0.5% DCP as a free-radical grafting initiator - 70/30 - 70/30 with 0.5% DCP - 50/50 - 50/50 with 0.5% DCP	-	-	- 1.00% - 2.00% - 4.00% - 2.00% - 11.00% - 4.00% - 15.00%	- (0.60) KJ/m^2^ - (1.50) KJ/m^2^ - (3.50) KJ/m^2^ - (3.00) KJ/m^2^ - (4.00) KJ/m^2^ - (3.00) KJ/m^2^ - (5.50) KJ/m^2^	Injection molding applications [193].
PHBV/PBS	- 80/20 - 80/20 with 0.2% DCP as a free-radical grafting initiator - 80/20 with 0.5% DCP - 80/20 with 1% DCP	-	-	- 8.00% - 200.00% - 400.00% - 350.00%	- (2.80) KJ/m^2^ - (3.00) KJ/m^2^ - (5.00) KJ/m^2^ - (5.50) KJ/m^2^	Injection molding applications [193].
PHBV/PLA/PBS	- 0/100/0 - 100/0/0 - 30/60/10 - 10/60/30 - 60/30/10 - 60/10/30	- 70.00 - 22.00 - 54.00 - 55.00 - 34.00 - 28.00	- 2750 - 1300 - 2300 - 2150 - 1750 - 1200	- 5.00% - 10.00% - 20.00% - 51.00% - 62.00% - 82.00%	- (17.5) J/m - (29.00) J/m - (33.00) J/m - (36.5) J/m - (30.00) J/m - (32.5) J/m	Structural materials [144].
PHB/PETG	- 100/0 - 90/10 - 80/20 - 70/30 - 60/40	- 58.00 - 49.00 - 42.00 - 33.00 - 33.00	-	-	- (24.00) J/m - (13.00) J/m - (22.00) J/m - (27.00) J/m - (15.00) J/m	Applications that require improved processability while miniating PHB’s biodegradability [192].
PHBV/P(3HB-*co*-3HV-*co*-3HHx)	- 100/0 - 90/10 - 75/25 - 50/50	- 34.00 - 40.00 - 30.00 - 25.00	- 4000 - 3950 - 2750 - 2000	- 2.00% - 1.800% - 2.60% - 3.30%	-	Organic recycling food packaging [201].
PHB-*g*AA/MWNTs-OH	- 100/0 - 99.5/0.5 - 99/1 - 97/3	- 16.00 - 23.50 - 33.50 - 26.50	-	- 8.0% - 7.0% - 6.0% - 4.0%	-	Applications that require higher performance [217].
PHB/starch	- 100/0 - 90/10 - 80/20 - 70/30 - 60/40 - 50/50	- 18.00 - 14.50 - 13.50 - 12.00 - 8.50 - 7.00	-	-	-	Applications requiring better biodegradation, thermal, mechanical properties as well as processibility [229].
	- 100/0 - 90/10 - 80/20 - 70/30 - 60/40 - 50/50 - 40/60 - 30/70	- 18.29 - 17.20 - 19.70 - 19.23 - 7.70 - 10.06 - 5.24 - 4.99	- 1708 - 1716 - 1085 - 949 - 856 - 694 - 686 - 578	- 3.32% - 9.80% - 6.00% - 9.40% - 8.50% - 5.27% - 3.45% - 4.30%	-	Low-cost coating material on cardboard or paper for food packaging [233].
	- 70/30 (starch contains 70% amylose) - 70/30 (starch contains 72% amylose)	- 12.50 - 7.30	-	- 3.90% - 2.80%	- 0.90 KJ/m^2^ - 0.70 KJ/m^2^	[235]
PHB-*g*AA/starch	- 100/0 - 90/10 - 80/20 - 70/30 - 60/40 - 50/50	- 16.00 - 17.00 - 16.00 - 15.50 - 15.00 - 14.90	-	-	-	Applications demanding better biodegradation, thermal, mechanical properties as well as processibility [229].
- Plasticized 70% amylose corn starch blended with thermoplastic partner (PCL or PBAT) followed by PHB addition. - PHB blended with thermoplastic partner (PCL or PBAT) followed by plasticized 70% amylose corn starch - Addition of PHB with thermoplastic partner (PCL or PBAT) with plasticized 70% amylose corn starch all in one step	-	- 15.00 - 18.00 - 21.00	- 900 - 1080 - 1020	- 47.00% - 32.00% - 114.00%	-	Flexible packaging [236].
PHB/ENR/MR/TMC	- 100/0/0/0 - 60/40/0/0 - 60/30/10/0 - 58/30/10/2 - 55/30/10/5 - 53/30/10/7	-	-	-	- (23.00) J/m - (25.00) J/m - (124.00) J/m - (93.00) J/m - (116.00) J/m - (87.00) J/m	Applications requiring high impact properties [259].
PHB/ENR/MR/COC	- 55/30/10/5	-	-	-	- (49.00) J/m	
PHB/MMT	- 100/0 - PHB/Cloisite^®^ Na+ - PHB/modified Cloisite^®^ 30B	- 29.60 - 24.90 - 27.00	- 3060 - 3200 - 3440	-	-	[208]
PHBH_x_/SiO_2_ fiber	- 100/0 ^c^ - 100/0 ^d^ - 99/1 ^c, e^ - 97/3 ^c, e^ - 95/5 ^c, e^ - 99/1 ^c, f^ - 97/3 ^c, f^ - 95/5 ^c, f^ - 99/1 ^d, e^ - 97/3 ^d, e^ - 95/5 ^d, e^ - 99/1 ^d, f^ - 97/3 ^d, f^ - 95/5 ^d, f^	- 23.00 - 24.50 - 24.50 - 22.50 - 24.60 - 23.00 - 24.50 - 23.50 - 24.40 - 25.00 - 24.50 - 25.00 - 24.50 - 24.50	- 1000 - 1300 - 1300 - 1400 - 1600 - 1300 - 1490 - 1400 - 1300 - 1400 - 1490 - 1400 - 1400 - 1490	-	-	Medical applications and tissue engineering [214].
PHBV/wheat starch	- 100/0 - 75/25 - 50/50	- 17.70 - 8.60 - 7.70	- 1525 - 2132 - 2498	- 25.00% - 5.10% - 1.00%	-	Complete biodegradable materials with reduced cost [225].
PHBV/maize starch	- 100/0 - 80/20 - 70/30 - 80/20 with 2% free radical former ^g^ - 70/30 with 2% free radical former ^g^	-	-	-	- 1.80 KJ/m^2^ - 1.20 KJ/m^2^ - 0.90 KJ/m^2^ - 2.10 KJ/m^2^ - 1.90 KJ/m^2^	Biodegradable disposable plastics with low cost and the required performance [226].
	- 100 - 80/20 - 70/30 - 60/40 - 50/50	- 18.00 - 6.00 - 3.50 - 4.00 - 2.85	- 1200 - 750 - 310 - 305 - 160	- 2.10% - 1.00% - 1.10% - 1.10% - 1.40%	-	Packaging [234].
PHBV/corn starch	- 75/25 with 5% Acetyl tributyl citrate as plasticizer	- 17.10	- 458.00	- 15.60%	-	Applications that require improved mechanical properties [228].
PHBV/starch-*g-*PGMA	- 75/25 with 5% Acetyl tributyl citrate as plasticizer	- 23.60	- 539.00	- 13.00%	-	
PHBV/granular cornstarch	- 100/0 with 10% Tiracetin as an additive - 70/30 with 10% Tiracetin - 50/50 with 10% Tiracetin - 70/30 with 10% Tiracetin and 9% PEO - 50/50 with 10% Tiracetin and 9% PEO - 50/50 with 10% Tiracetin and 5% PEO - 50/50 with 10% Tiracetin and 2% PEO	- 24.00 - 15.00 - 10.00 - 19.00 - 18.00 - 15.00 - 12.00	- 180 - 250 - 300 - 220 - 170 - 210 - 280	- 38.00% - 21.00% - 11.00% - 21.00% - 21.00% - 15.00% - 10.00%	-	Single use applications such as plastic knives and forks [222].
PHBV/Cloisite^®^ 30B	- 100/0 - 99/1 - 97/3 - 95/5	- 37.50 - 40.60 - 30.70 - 30.40	- 3500 - 5100 - 4600 - 7100	- 3.00% - 2.40% - 0.70% - 0.60%	- (20.00) J/m - (11.00) J/m - (11.00) J/m - (10.00) J/m	[209]
	- 100/0 - 99/1 - 98/2 - 97/3	- 31.00 - 32.00 - 35.00 - 33.00	- 481 - 555 - 730 - 795	- 8.50% - 7.60% - 7.70% - 5.60%	-	Applications that require enhanced processing behaviors, crystallinity, low cost and improved mechanical properties [264].
PHBV/PLA/Cloisite^®^ 30B	- 15/85/0 - 15/85/4 - 30/70 - 30/70/4	- 52.00 - 49.00 - 46.00 - 44.50	- 1600 - 2000 - 1750 - 2000	- 8.80% - 8.00% - 5.00% - 2.50%	-	Injection molding applications with high modulus, heat deflection resistance and superior gas barrier properties [194].
PHBV/Cloisite^®^ 15A	- 100/0 - 99/1 - 97.5/2.5 - 95/5	- 5.90 - 11.80 - 18.00 - 28.90	- 633.00 - 1043.00 - 1311.00 - 1677.00	- 3.30% - 2.70% - 1.80% - 1.40%	-	Applications demanding enhanced mechanical properties [254].
PHBV/OMMT	- 97/3 - 90/10	- 26.90 - 35.60 - 21.80	- 1373 - 1412 - 1375	- 4.10% - 3.90% - 2.10%	-	Applications that require enhanced crystallization and mechanical properties [207].
PHBV/LDH-SA	- 100/0 - 99/1 - 97/3 - 95/5 - 93/7	- 25.10 - 28.20 - 28.50 - 24.20 - 24.40	- 1120 - 1230 - 1330 - 1420 - 1240	- 4.03% - 3.50% - 3.25% - 2.50% - 2.70%	-	Medical applications [267].
PHBV/HNT	- 100/0 - 99/1 - 97/3 - 95/5	- 37.50 - 39.30 - 39.40 - 38.70	- 3500 - 4000 - 3600 - 5700	- 3.00% - 3.70% - 3.10% - 4.10%	- (20.00) J/m - (19.00) J/m - (20.00) J/m - (19.00) J/m	[209]
PHBV/CNW	- 100/0 - 98/2 with PEG as a compatibilizer - 95/5 with PEG	- 14.10 - 15.50 - 26.10	- 820 - 1100 - 1760	- 12.40% - 7.10% - 7.80%	-	Sustainable composite applications [213].
PHBV/CS	- 100/0 - 100/0 with 10% ATBC as a plasticizer and 5% CaCO_3_ - 85/5 with 10% ATBC and 5% CaCO_3_ - 85/7.5 with 10% ATBC and 5% CaCO_3_ - 85/10 with 10% ATBC and 5% CaCO_3_ - 85/12.5 with 10% ATBC and 5% CaCO_3_	- 34.80 - 23.00 - 20.80 - 19.70 - 18.40 - 17.30	- 2610 - 1300 - 1730 - 1930 - 2030 - 2050	- 2.60% - 6.20% - 4.00% - 2.90% - 2.50% - 2.30%	- 2.50 KJ/m^2^ - 5.80 KJ/m^2^ - 3.70 KJ/m^2^ - 3.70 KJ/m^2^ - 3.20 KJ/m^2^ - 3.80 KJ/m^2^	Food contact injection molding applications such as coffee capsules [273].
PHA/TFSB	- 100/0 - 99/1 - 98/2 - 97/3 - 96/4 - 95/5	- 1.66 - 1.51 - 1.41 - 1.36 - 1.22 - 1.08	-	- 85.30% - 78.80% - 73.60% - 67.10% - 62.50% - 57.10%	-	Biomedical applications, such as bioprotective films, wound healing and bone tissue engineering (e.g., bone screws, bone joints and tooth roots) [275].
MPHA/TFSB	- 100/0 - 99/1 - 98/2 - 97/3 - 96/4 - 95/5	- 1.60 - 1.88 - 2.13 - 2.43 - 2.36 - 2.24	-	- 86.10% - 76.30% - 67.80% - 59.30% - 52.50% - 39.60%	-	
PHA/GNPs	- 100/0 - 97.5/2.5 - 95/5 - 92.5/7.5 - 90/10 - 85/15	- 17.00 - 16.40 - 14.00 - 18.00 - 11.00 - 11.20	-	- 14.00% - 10.00% - 11.00% - 12.00% - 3.00% - 3.00%	-	Thermal and electrical applications [220].
PHA/GNPs and CNFs	- 97.5/2.5 - 95/5 - 92.5/7.5 - 90/10 - 85/15	- 16.40 - 16.50 - 16.00 - 17.10 - 17.00	-	- 12.00% - 9.00% - 6.00% - 5.50% - 2.00%	-	
Mixture of P3HB and P4HB /FD-TMCNCs	- 100/0 - 95/5 - 90/10	- 25.84 - 19.20 - 17.02	- 5.27 - 5.52 - 3.64	- 101.33% - 301.00% - 231.00%		Commercial applications demanding high ductility [216].
Mixture of P3HB and P4HB /TD-TMCNCs	- 90/10	- 15.58	- 0.09	- 247.67%		

^a^ Plasticized PHA containing 65% PHA, 30% PBS and 5% crosslinking agent. ^b^ Quasi-static tensile testing. ^c^ Molecular weight of PHBHx = 903,000 g/mol. ^d^ Molecular weight of PHBHx = 633,000 g/mol ^e^ SiO_2_ fiber. ^f^ SiO_2_ sphere. ^g^ bis[tert-butylperoxyisopropyl] benzene. Note: Studies in which no exact values for the mechanical properties were given, the best estimations were provided. Abbreviations: PHB, polyhydroxybutyrate; PCL, poly(ε-caprolactone); P(3HO-3HD), poly(3-hydroxyoctanoate-*co*-3-hydroxydecanoate); PBS, poly(butylenes succinate); DCP, dicumyl peroxide; PHBV, poly(3-hydroxybutyrate-*co*-3-hydroxyvalerate); PETG, poly(ethylene terephthalate-*co*-1,4-cyclohexenedimethanol terephthalate); P(3HB-*co*-3HV-*co*-3HHx), poly(3-hydroxybutyrate-*co*-3-hydroxyvalerate-*co*-3-hydroxy-hexanoate); PHB-*g*AA, acrylic acid grafted PHB; MWNTs, multiwalled carbon nanotubes; MWNTs-OH, multihydroxyl functionalized MWNTs; PBAT, poly(butylene adipate-*co*-terephthalate); ENR, epoxidized natural rubber; MR, maleated rubber; TMC, titanate modified clay; COC, commercially modified clay; MMT, montmorillonite; Cloisite^®^ Na+, NA-MMT; PHBH_x_, poly(3-hydroxybutyrate-*co*-3-hydroxyhexanoate); SiO_2_, silica; starch-*g*-PGMA, starch-*g*-poly (glycidyl methacrylate); PEO, polyethylene oxide; PLA, poly(lactic acid); OMMT, organically modified montmorillonite; LDH, layered double hydroxide; LDH-SA, Zn-Al NO3 LDH organically modified with stearic acid; HNT, halloysite; CNW, freeze-dried cellulose nano whiskers; PEG, polyethylene glycol; CS, coffee silverskin; ATBC, acetyl tributyl citrate; CaCO3, calcium carbonate; TFSB, treated fish scale powder; MPHA, modified PHA (produced by mixing PHA with 10 phr PHA-*g*-AA, then heating); GNPs, graphene nanoplatelets; CNFs, carbon nanofibers; P3HB, poly-3-hydroxybutryate; P4HB, poly-4-hydroxybutryate; CNCs, cellulose nanocrystals; FD-TMCNCs, freeze-dried CNCs with CH_3_ ends treated by tetraethyl orthosilicate (TEOS) and methyltrimethoxysilane (MTMS); TD-TMCNCs, thermally-dried CNCs with CH_3_ ends treated by TEOS and MTMS.

**Table 15:** Features and applications of some selected studies on PHAs blends and nanocomposites.

Blend/Nanocomposite	Concentration (wt.%)	Features	Applications and/or Reference
PHB/PLA	- 80/20 and 60/40	-Improvement in the percentage elongation at break. Percentage elongation at break for the PHB/PLA 60/40 wt.% was eight time that of the neat PHB.	-Biomedical applications [133].
PHB/PCL	- 30/70	-An increase in the crystallinity of PHB and the blend PHB-phase. -Complete degradation.	-Biotechnological applications [186].
	- 75/25, 50/50 and 25/75	-Increase in the blend’s flexibility and ductility. -Significant increase in the percentage elongation at break and the energy absorption in impact conditions.	[195]
	- 65/30 with 5% crosslinking agent	-Increase in the percentage elongation at break as well as the tensile strength in the quasi-static tensile test.	-Multi-scale instrumental analyses [197].
PHBV/PBS	- 90/10, 80/20 and70/30	-Significant enhancement in the elongation at break of the PHBV/PBS blends due to the better interfacial adhesion between the PHBV and PBS phases. -Improvement in tensile strength.	[193]
PHBV/PLA/PBS	- 60/30/10 and 60/10/30	-Entirely biodegradable. -An enhancement in the PLA’s crystallization, flexibility and toughness was observed in the resulting ternary complex. -Optimum performance with excellent balanced thermal resistance and stiffness-toughness.	[144]
PHB/LDPE	-	-Substantial improvement in the LDPE’s modulus of elasticity.	[191]
PHBV/PE	- 10/90 and 30/70	-The rate of degradation was proportional to the quantity of PHBV in contact with PE.	[188]
PHB/PETG	- 80/20, 60/40, 50/50, 40/60 and 20/80	-Substantial enhancement in the flexural modulus. -Improvement in the processability and modulus of elasticity without significant changes in the impact resistance while keeping the biodegradability of PHB intact.	[192]
PHBV/MWNTs	- 98/2	-Improvement in thermal stability.	-Applications that require higher thermal stability, hardness and improved electrical conductivity [204].
	- 99/1, 97/3, 95/5 and 93/7	-Enhancement in the crystallinity and crystallite sizes of PHBV.	[203]
PHBV/CNTs	- 99/1, 97/3, 95/5 and 90/10	-Improvement in water and oxygen transmission, barrier properties, conductivity and mechanical properties.	-Medicine, aerospace engineering, home appliances, public transportations as well as beverage and food packaging [215].
PHBV/CNFs			
P3HB-*co*-4HB/CNFs	- 99/1 treated with *n*-octanol, silane coupling agent (KH-550) and nitric acid	-Both, the crystallinity and the glass transition temperature increased.	-Biomedical and electronic applications [218].
mcl-PHAs/CNFs	-	-Improvement in the crystallinity, thermomechanical properties and physical morphology.	-Smart biomaterials, such as: biosensors, organic electroconductive materials and stimuli-responsive drug delivery devices [219].
PHBV/plasticized (with Glycerol) wheat starch	-	-Low cost. -Sufficient adhesion between layers. -Moisture barrier properties. -Satisfactory water resistance. -Improved mechanical properties.	-Compostable multilayers film for disposable articles and food packaging [237].
PHBV/extruded starch	- 95/5, 90/10 and 80/20	-The expansion of the foam has been reduced.	-Loose fill packaging applications [240].
PHBV/TPS	-	-Cost effective. -PHA works as a water-resistant outer coating.	-Food packaging, insect dies, controlled drug release and pesticides [239].
PHB/organo-modified fluoromica	- 98/2	-Significant enhancement in mechanical and thermal properties as well as the biodegradation rate.	[205]
PHB/MMT	- 98.8/1.2, 97.7 and 96.4/3.6		
Nodax^®^™/clay 20A and clay 25A	- 99/1, 97/3, 95/5, 93/7, 90/10 and 85/15	-Improved mechanical properties and a slight enhancement in thermal stability.	-Applications that Require improved mechanical properties [265].
PHB/PMLDH	- 98/2 and 95/5	-Significant enhancement in storage modulus. -An increase in crystallization rate. -A reduction in activation energy.	[255]
mcl-PHAs/hydrolyzed tunicin cellulose whiskers	-	-Substantial enhancement in mechanical properties.	[268]

Abbreviations: PHB, polyhydroxybutyrate; PLA, poly(latic acid); PCL, poly(ε-caprolactone); PHBV, poly(3-hydroxybutyrate-*co*-3-hydroxyvalerate); PBS, poly(butylenes succinate); LDPE, low-density polyethylene; PE, poly(ethylene); PETG, poly(ethylene terephthalate-*co*-1,4-cyclohexenedimethanolterephthalate); MWNTs, multiwalled carbon nanotubes; CNTs, carbon nanotubes; CNFs, carbon nanofibers; P3HB-*co*-4HB, poly-3-hydroxybutyrate-*co*-4-hydroxybutyrate; mcl-PHAs, medium chain length poly3-hydroxyalkanoate; TPS, thermoplastic starch; MMT, montmorillonite; Nodax^®^™, [poly(3-hydroxybutyrate-*co*-3-hydroxyhexanoate)]; clay 20A, Cloisite 20A; clay 25A, Cloisite 25A; PMLDH, modified layered double hydroxide.
